# Regioselective cobalt(II)-catalyzed [2 + 3] cycloaddition reaction of fluoroalkylated alkynes with 2-formylphenylboronic acids: easy access to 2-fluoroalkylated indenols

**DOI:** 10.3762/bjoc.16.184

**Published:** 2020-09-04

**Authors:** Tatsuya Kumon, Miroku Shimada, Jianyan Wu, Shigeyuki Yamada, Tsutomu Konno

**Affiliations:** 1 Faculty of Molecular Chemistry and Engineering, Kyoto Institute of Technology, Matsugasaki, Sakyo-ku, Kyoto 606-8585, Japan

**Keywords:** [2 + 3] cycloaddition, cobalt catalyst, fluorine-containing, indenols, regioselective

## Abstract

[2 + 3] cycloaddition reactions of fluorinated alkynes with 2-formylphenylboronic acids under the influence of Co(acac)_2_·2H_2_O in two-component solvents of acetonitrile/2-propanol at reflux temperature for 18 h took place smoothly, affording the corresponding fluoroalkylated indenol derivatives in good yields. This reaction shows excellent regioselectivity, giving 2-fluoroalkylated indenols, together with a very small amount of 3-fluoroalkylated indanones as side products.

## Introduction

**Figure 1:**
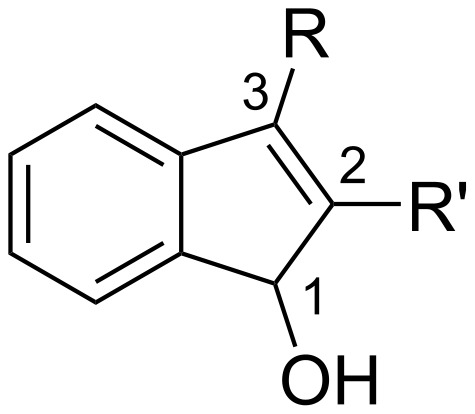
Indenol skeleton.

**Scheme 1:**
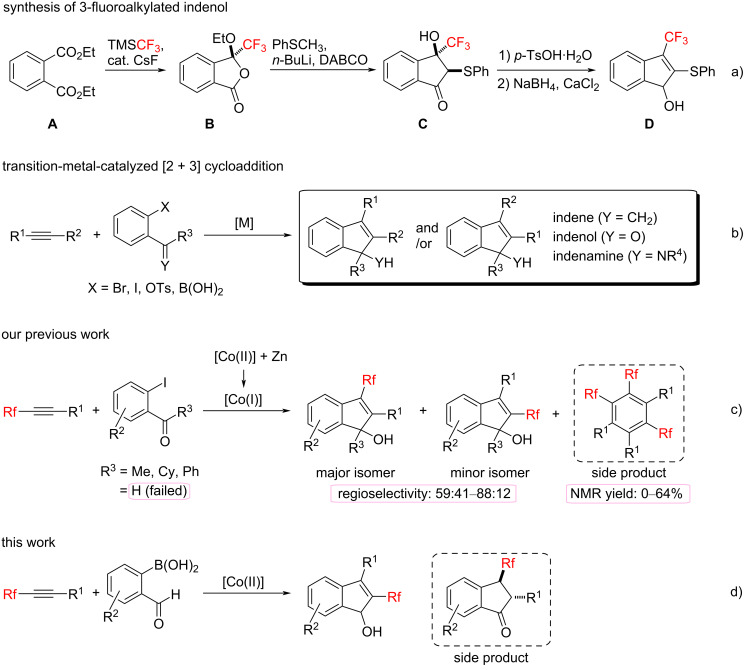
Synthesis of 2,3-disubstituted indene derivatives.

2,3-Disubstituted indenol derivatives are important compounds possessing high potential due to the insecticidal, myorelaxation, and antiproliferative properties (Figure 1) [1–12]. Thus, enormous attention has been paid to 2- or 3-fluoroalkylated indenol derivatives in the field of medicinal and agrochemical drug design since a fluorine atom can very often bring about an increasing effect on the pharmacological activity owing to the unique nature of the fluorine atom(s) [13–18]. However, reports on a synthetic protocol for 2- or 3-fluoroalkylated indenols are very limited [19–22]. Recently, Yamazaki et al. have reported the reaction of the CF_3_-containing phthalide **B** prepared from the 1,2-diester **A** with the Ruppert–Prakash reagent (TMSCF_3_) in the presence of a catalytic amount of CsF (Scheme 1a). According to this paper, phthalide **B** reacted smoothly with phenylthiomethyllithium to give the corresponding CF_3_-containing indanone **C**. Then, dehydration of **C** using *p*-TsOH⋅H_2_O, followed by a 1,2-reduction led to 3-fluoroalkylated indenol **D**. All of these reactions produced the desired products in good yield, however, this protocol is limited to the introduction of a trifluoromethyl group at the 3-position of the indenol. To the best of our knowledge, there are no reports on the practical synthesis of disubstituted 2-fluoroalkylated indenols so far.

Transition-metal-catalyzed carbocyclization reactions of alkynes with benzene derivatives having a leaving group X (X = Br, I, OTs, B(OH)_2_) have been widely considered as one of the most efficient and convenient protocols for the construction of various 2,3-disubstituted indene derivatives, such as indenols and indenamines (Scheme 1b) [23–25]. There have been numerous studies on the reaction with nonfluorinated alkynes under the influence of various transition metals. Despite their effective advantages, on the other hand, the reports on cycloaddition reactions with fluorine-containing alkynes for the construction of fluoroalkylated indene derivatives are rare [21,26].

Recently, our group has reported the first practical synthesis of fluoroalkylated indenol derivatives using various fluoroalkylated alkynes with 2-iodoaryl ketones via cobalt-catalyzed carbocyclizations (Scheme 1c) [21]. Although our previous work was practical to produce interesting fluoroalkylated indenol derivatives, some drawbacks still remain unsolved. Initially, the reaction showed a low regioselectivity, leading to a mixture of 3-fluoroalkylated and 2-fluoroalkylated indenols, which was difficult to separate. Secondly, a trimer of fluoroalkylated alkynes was obtained as a side product since the cobalt(I) species produced by the cobalt(II)/Zn system worked as a suitable catalyst, leading to the corresponding trimer of the fluoroalkylated alkyne, as reported by our group [27]. Finally, only 2-iodoaryl ketones (R^3^ = Me, Cy, Ph) were applicable in this catalytic reaction, whereas the cycloaddition using 2-iodobenzaldehyde (R^3^ = H) did not work at all. Therefore, the development of practical protocols for [2 + 3] cycloaddition reactions with a broader substrate scope for the synthesis of fluoroalkylated indenols is still required. Herein we present a synthesis of 2-fluoroalkylated indenols via [2 + 3] cycloadditions of various fluorinated alkynes with 2-formylphenylboronic acids in the presence of cobalt(II) species as a catalyst to suppress the trimerization products (Scheme 1d).

## Results and Discussion

Initially, we carried out the screening of the reaction conditions for the cobalt-catalyzed [2 + 3] cycloaddition using fluoroalkylated alkyne **1a** and 2-formylphenylboronic acid (**2A**) [28]. The results are summarized in Table 1.

**Table 1:** Screening for the reaction conditions of the cobalt-catalyzed [2 + 3] cycloaddition using the fluoroalkylated alkyne **1a** and 2-formylphenylboronic acid (**2A**).

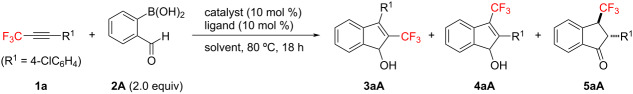						

entry	solvent	catalyst	ligand	yield^a^/%	ratio^a^	yield^a^/%
				**3aA** + **4aA**	**3aA**/**4aA**	**5aA**

1	CH_3_CN	Co(acac)_2_·2H_2_O	dppe	76	66:34	12
2	DCE	Co(acac)_2_·2H_2_O	dppe	8	49:51	0
3	iPrOH	Co(acac)_2_·2H_2_O	dppe	6	>99:1	3
4	1,4-dioxane	Co(acac)_2_·2H_2_O	dppe	14	64:36	1
5	CH_3_CN/iPrOH 3:1, v/v	Co(acac)_2_·2H_2_O	dppe	48	>99:1	48
6	CH_3_CN/iPrOH 3:1, v/v	CoCl_2_	dppe	81	66:34	16
7	CH_3_CN/iPrOH 3:1, v/v	Co(OAc)_2_·4H_2_O	dppe	24	>99:1	24
8	CH_3_CN/iPrOH 3:1, v/v	Co(acac)_3_	dppe	15	70:30	11
9	CH_3_CN/iPrOH 3:1, v/v	Co(OH)_2_	dppe	8	39:61	3
10	CH_3_CN/iPrOH 3:1, v/v	Co(acac)_2_·2H_2_O	dppp	59	98:2	3
11	CH_3_CN/iPrOH 3:1, v/v	Co(acac)_2_·2H_2_O	dppb	trace	>99:1	2
12^b^	CH_3_CN/iPrOH 3:1, v/v	Co(acac)_2_·2H_2_O	dppp	75	>99:1	6
13^c^	CH_3_CN/iPrOH 3:1, v/v	Co(acac)_2_·2H_2_O	dppp	49	73:27	–

^a^Determined by ^19^F NMR spectroscopy. ^b^Carried out at the reflux temperature. ^c^2-Acetylphenylboronic acid was used.

The cycloaddition of the fluoroalkylated alkyne **1a** with 2.0 equiv of 2-formylphenylboronic acid (**2A**) in the presence of 10 mol % each of Co(acac)_2_·2H_2_O and 1,2- bis(diphenylphosphino)ethane (dppe) in CH_3_CN at 80 °C for 18 h proceeded to afford the corresponding cyclic products **3aA** and **4aA** in 76% yield as a regioisomeric 66:34 mixture (Table 1, entry 1). Intriguingly, a small amount of the undesired *trans*-3-fluoroalkylated indanone **5aA** was obtained as a side product, whereas the *cis*-3-fluoroalkylated and *cis/trans*-2-fluoroalkylated indanones were not observed. As shown in Table 1, entries 2–4, changing the solvent from CH_3_CN to DCE, iPrOH, or 1,4-dioxane caused an appreciable decrease in the yield. It should be noted that the use of a mixed solvent of CH_3_CN and iPrOH significantly improved the isomeric ratio of **3aA** and **4aA** (>99:1), although 48% of the indanone **5aA** was also obtained (Table 1, entry 5). The cycloaddition reaction in the presence of CoCl_2_ instead of Co(acac)_2_·2H_2_O produced the desired **3aA** and **4aA** in 81% yield, with a lower selectivity of 66:34 (Table 1, entry 6), while the other cobalt catalysts, such as Co(OAc)_2_·4H_2_O, Co(acac)_3_, and Co(OH)_2_ resulted in the sluggish formation of the fluoroalkylated indenols or indanone (Table 1, entries 7–9). When 1,3-bis(diphenylphosphino)propane (dppp) was used as a phosphine ligand, the desired indenol **3aA** was obtained in a moderate yield and with high regioselectivity of 98:2, together with a very small amount of the fluoroalkylated indanone **5aA** (Table 1, entry 10). On the other hand, 1,4-bis(diphenylphosphino)butane (dppb) was not a suitable ligand for this reaction, leading only to trace amount of the indanone **5aA** (Table 1, entry 11). Finally, the desired cyclization product was produced in a good yield at the reflux temperature while maintaining the selectivity (>99:1, Table 1, entry 12). Unfortunately, 2-acetylphenylboronic acid instead of 2-formylphenylboronic acid (**2A**) showed a lower reactivity and a poor regioselectivity (73:27, Table 1, entry 13) [29].

**Scheme 2:**
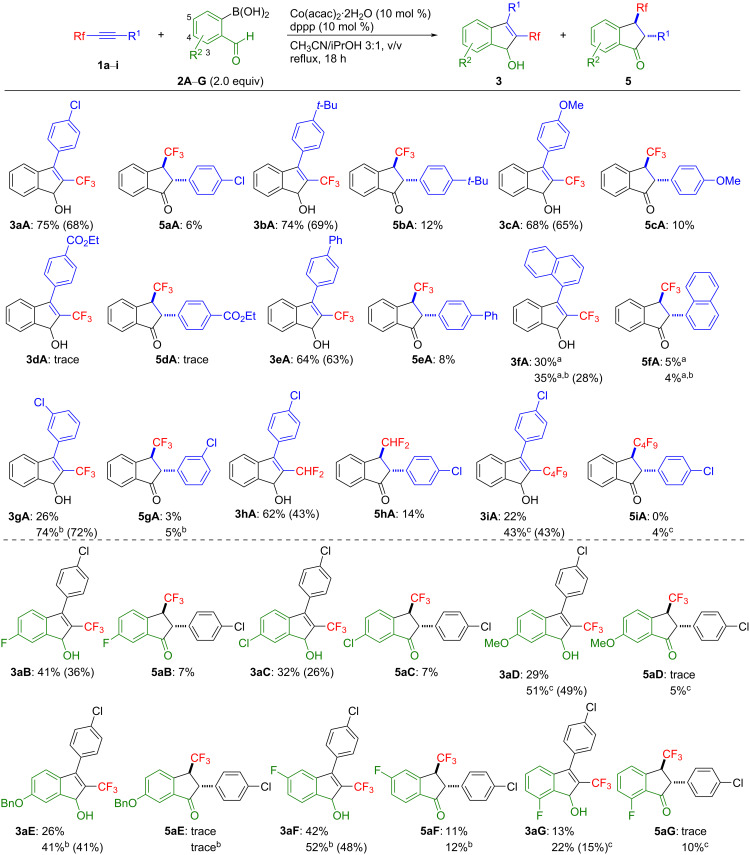
Cobalt-catalyzed [2 + 3] cycloaddition reaction of the fluorinated alkynes **1** with various 2-formylphenylboronic acids **2**. The yields were determined by ^19^F NMR spectroscopy. The values in parentheses are the isolated yield of **3**. ^a^Atropisomers of **3fA** and **5fA** were detected. ^b^3.0 equiv of **2** were used. ^c^The reaction was carried out using 3.0 equiv of **2** in the presence of 20 mol % each of Co(acac)_2_·2H_2_O and dppp at 110 °C in a sealed tube.

With the optimized reaction conditions (Table 1, entry 12), we explored the substrate scope for the [2 + 3] cycloaddition of various fluoroalkylated alkynes **1** and 2-formylphenylboronic acids **2**. The results are summarized in Scheme 2.

The substrates **1**, having an electron-donating substituent on the benzene ring of the fluoroalkylated alkyne, such as *t*-Bu or MeO, reacted efficiently, leading to the desired fluoroalkylated indenols **3bA** and **3cA** in 74% and 68% yield, respectively. However, the substrate containing the electron-withdrawing group CO_2_Et on the benzene ring of **1** showed a lower reactivity in this reaction (see **3dA**). The cycloaddition reaction using a fluoroalkylated alkyne having a 4-biphenyl group as R^1^ took place smoothly, giving the corresponding indenol **3eA** in a good yield. Though bulkier groups as R^1^, such as 1-naphthyl or 3-chlorophenyl, reduced the reactivity (see **3fA** and **3gA**), an excess loading of the boronic acid improved the yield appreciably. For the difluoromethylated alkyne **1h**, the desired indenol **3hA** was obtained in 62% yield under optimal conditions. However, the nonafluorobutylated alkyne **1i** showed a lower reactivity, giving the corresponding indenol **3iA** in 22% yield. Therefore, when the reaction was carried out using 3.0 equiv of the boronic acid **2A** and 20 mol % each of Co(acac)_2_·2H_2_O and dppp at 110 °C, the desired 2-fluoroalkylated indenol **3iA** was obtained in 43% yield.

Subsequently, we investigated the [2 + 3] cycloaddition reaction of the fluoroalkylated alkyne **1a** (R^1^ = 4-ClC_6_H_4_) with variously substituted 2-formylphenylboronic acids **2**. Electron-deficient formylphenylboronic acids possessing a fluorine or chlorine atom on the benzene ring gave the corresponding indenols **3aB** and **3aC** in 41% and 32% yield, respectively, which could not be improved even when an excess loading of the reagents, prolonging the reaction time, and a higher reaction temperature were applied. However, the exposure of the fluoroalkylated alkynes **1a** to electron-rich substrates, e.g., **2D** and **2E**, lead to the indenols in approximately 30% yield, and the use of an excessive amount of boronic acid, Co(acac)_2_·2H_2_O, or dppp improved the yields of the desired indenols, forming **3aD** in 51% and **3aE** in 41% yield. Changing the substituent position on the aromatic ring of the phenylboronic acid from the 4- to the 5-position did not affect the result, giving the indenol **3aF** in 52% yield. However, a boronic acid with a fluorine atom at the 3-position did not lead to a satisfactory result, with the cyclic product **3aG** being formed in only 22% yield.

**Scheme 3:**
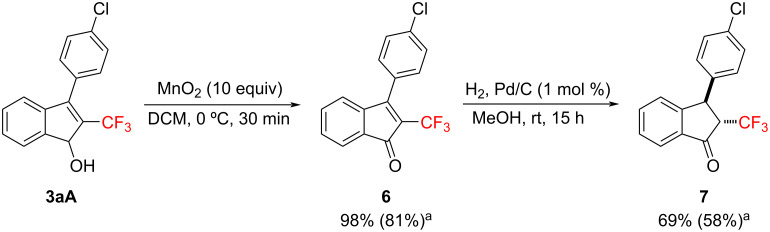
Synthesis of the fluoroalkylated indenone **6** and the indanone **7** from the indenol **3aA**. The yields were determined by ^19^F NMR spectroscopy. The values in parentheses are the isolated yields.

Substituted indenones and indanones widely exist in nature, and these skeletons are important classes of organic compounds due to diverse biological and pharmacological activities [30–32]. Therefore, we also accomplished the synthesis of 2-fluoroalkylated indenone and indanone by simple reactions (Scheme 3). An allylic oxidation was carried out with 10 equiv of manganese dioxide in dichloromethane as the solvent at 0 °C for 30 minutes, leading to the corresponding 2-fluoroalkylated indenone **6** in 98% yield. Subsequently, a hydrogenolysis with 1 mol % of Pd/C under a hydrogen atmosphere in methanol at room temperature for 15 h produced the desired 2-fluoroalkylated indanone **7** as the *trans*-isomer in 69% yield [33–35].

**Scheme 4:**
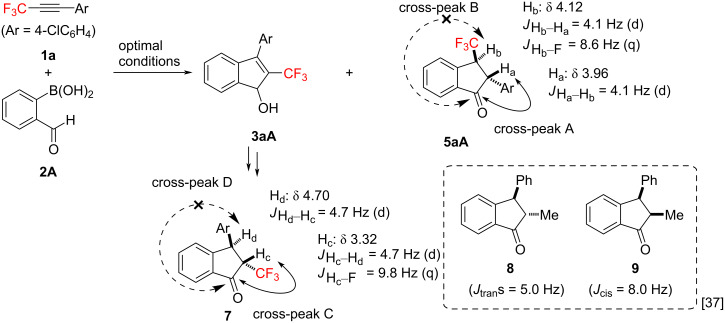
Stereochemical assignment of **5aA** and **7** based on NMR techniques. The cross-peaks were observed through HMBC measurements.

The stereochemical assignment of **5aA** and **7** was carried out based on NMR techniques, as shown in Scheme 4 [36]. A strong correlation between the carbonyl carbon atom and H_a_ was obtained, whereas the cross-peak between the carbonyl carbon atom and H_b_ was not observed, strongly indicating that the indanone **5aA** possessed a CF_3_ group at the 3-position. On the other hand, the cross-peak between the carbonyl carbon atom and H_c_ of the indanone **7** was obtained, but there was no cross-peak between the carbonyl carbon atom and H_d_, meaning that the indanone **7** has a CF_3_ group at the 2-position. This result indicated that the indenol **3aA**, which is the precursor of the 2-fluoroalkylated indanone **7**, also possess a trifluoromethyl group at the 2-position. Each H_a_–H_b_ coupling of **5aA** and each H_c_–H_d_ coupling of **7** appeared as a doublet, with *J* = 4.0 Hz and 4.6 Hz, respectively. These NMR results suggested that the fluoroalkylated indanones **5aA** and **7** were *trans*-isomers, and previous results showed that the *J* value of the nonfluoroalkylated *trans*-2,3-disubstituted indanone **8** is 5.0 Hz, whereas the *J* value of the *cis*-isomer **9** is 8.0 Hz [37].

**Scheme 5:**
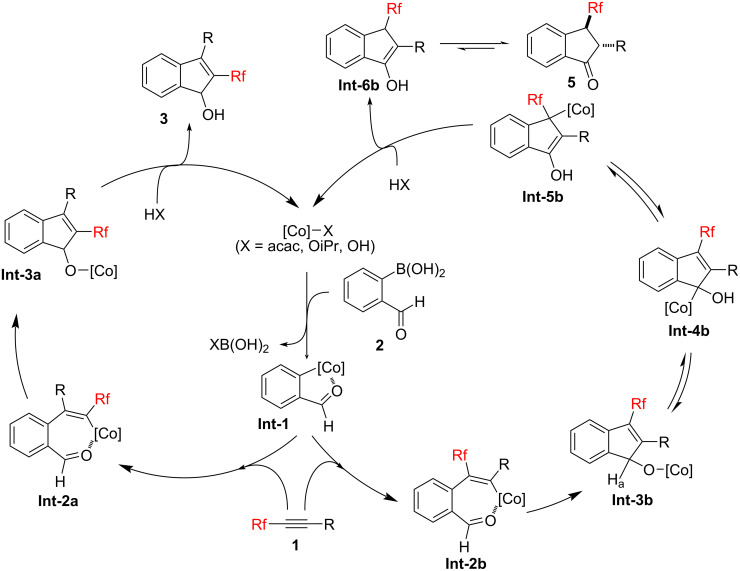
Proposed reaction mechanism.

The proposed catalytic cycles for the cobalt-catalyzed [2 + 3] cycloaddition process leading to 2-fluoroalkylated indenols and 3-fluoroalkylated indanones are shown in Scheme 5 [28,38]. Thus, the reaction presumably proceeds as follows: (1) transmetalation of the cobalt catalyst with 2-formylphenylboronic acids (**2**) gives the arylcobalt species **Int-1**, (2) insertion of the alkyne **1** into the [Co]–Ar bond (see **Int-2a**) [39], (3) migration insertion into the formyl moiety to afford the corresponding cobalt alkoxide **Int-3a**, (4) protonation of **Int-3a** with HX (X = acac, OiPr, or OH), giving rise to the desired 2-fluoroalkylated indenol **3**.

The formation of the 3-fluoroalkylated indanones as a side product may be explained based on the previous literature [20,40]. The reaction of **Int-1** with the alkynes **1** also gives **Int-2b** as a regioisomeric intermediate of **Int-2a**, leading to the 3-fluoroalkylated cobalt alkoxide **Int-3b**. Subsequently, the proton shift of the cobalt alkoxide **Int-3b** provides the allylcobalt species **Int-4b** because the acidity of the proton H_a_ of **Int-3b** is the same as, or higher than the hydroxy group of **Int-4b** due to the electron-withdrawing effect of the fluoroalkyl group. It should be noted that this process is probably accelerated by 2-propanol or H_2_O, which is explained by the results that the most 3-fluoroalkylated indenol **4aA** was converted into the indanone **5aA** in the reaction, using 2-propanol as the solvent and a cobalt hydrate as a catalyst, such as Co(acac)_2_·2H_2_O and Co(OAc)_2_·4H_2_O, respectively (Table 1, entries 3, 5, and 7).

The compound **Int-4b** produced the allylcobalt species **Int-5b** with a stabilized C–[Co] bond due to the electron-withdrawing ability of the fluoroalkyl group. The **Int-5b** species undergoes protonation at the carbon bonded to the fluoroalkyl group, giving the enol **Int-6b**. Finally, the enol **Int-6b** produces the 3-fluoroalkylated indanone **5** via keto–enol tautomerism.

## Conclusion

In conclusion, we developed a practical and efficient synthetic protocol for 2-fluoroalkylated indenol derivatives via a cobalt-catalyzed [2 + 3] cycloaddition reaction using fluorinated alkynes and 2-formylphenylboronic acids. It was revealed that the reaction using dppp as a ligand showed a high regioselectivity, leading to 2-fluoroalkylated indenols in good yield. Moreover, the side product was a small amount of 3-fluoroalkylated indanones, which was easily separated from the 2-fluoroalkylated indenols due to the lower polarity of indanones compared to indenols. Finally, 2-fluoroalkylated indenol was simply converted into the corresponding 2-fluoroalkylated indenone and indanone in good to excellent yield.

## Supporting Information

File 1 Experimental procedures, characterization data (^1^H, ^13^C, ^19^F NMR, IR, and HRMS), copies of ^1^H, ^13^C, and ^19^F NMR spectra.
