# Supplementary material for: Regioselective cobalt(II)-catalyzed [2 + 3] cycloaddition reaction of fluoroalkylated alkynes with 2-formylphenylboronic acids: easy access to 2-fluoroalkylated indenols
Source: Beilstein J Org Chem. 2020 Sep 4;16:2193–200. doi: 10.3762/bjoc.16.184 (PMC7476587; doi:10.3762/bjoc.16.184)
Supplement: File 1 — Experimental procedures, characterization data (1H, 13C, 19F NMR, IR, and HRMS), copies of 1H, 13C, and 19F NMR spectra. [file Beilstein_J_Org_Chem-16-2193-s001.pdf]

BEILSTEIN JOURNAL OF ORGANIC CHEMISTRY

Supporting Information

for

Regioselective cobalt(II)-catalyzed [2 + 3] cycloaddition reaction of fluoroalkylated alkynes with 2-formylphenylboronic acids: easy access to 2-fluoroalkylated indenols

Tatsuya Kumon, Miroku Shimada, Jianyan Wu, Shigeyuki Yamada and Tsutomu Konno

Beilstein J. Org. Chem. **2020,** *16,* 2193–2200. doi:10.3762/bjoc.16.184

Experimental procedures, characterization data (^{1}H, ^{13}C, ^{19}F NMR, IR, and HRMS), copies of ^{1}H, ^{13}C, and ^{19}F NMR spectra

License and Terms: This is a supporting information file under the terms of the Creative Commons Attribution License (https://creativecommons.org/licenses/by/4.0). Please note that the reuse, redistribution and reproduction in particular requires that the authors and source are credited.
The license is subject to the *Beilstein Journal of Organic Chemistry* terms and conditions: (https://www.beilstein-journals.org/bjoc)

Table of contents S1

1. General informations S3

2. Cobalt catalyzed [2 + 3] cycloaddition and charactalization of products S4

2-1. Typical procedure S4

2-2. Charactalization of 2- and 3-fluoroalkylated indenols S4

3. Synthesis of 2-fluoroalkylated indanone S9

3-1. Typical procedure S9

3-2. Charactalization of 2-fluoroalkyltaed indenone and indanone S9

4. Copies of ^{1}H, ^{13}C, ^{19}F NMR, and HMBC spectra for new compounds S11

^{1}H NMR spectrum of 3-(4-Chlorophenyl)-2-trifluoromethyl-1*H*-inden-1-ol (**3aA**) S11

^{13}C NMR spectrum of 3-(4-Chlorophenyl)-2-trifluoromethyl-1*H*-inden-1-ol (**3aA**) S11

^{19}F NMR spectrum of 3-(4-Chlorophenyl)-2-trifluoromethyl-1*H*-inden-1-ol (**3aA**) S12

^{1}H NMR spectrum of 3-[4-(1,1-Dimethylethyl)phenyl]-2-trifluoromethyl-1*H*-inden-1-ol (**3bA**) S13

^{13}C NMR spectrum of 3-[4-(1,1-Dimethylethyl)phenyl]-2-trifluoromethyl-1*H*-inden-1-ol (**3bA**) S13

^{19}F NMR spectrum of 3-[4-(1,1-Dimethylethyl)phenyl]-2-trifluoromethyl-1*H*-inden-1-ol (**3bA**) S14

^{1}H NMR spectrum of 3-(4-Methoxyphenyl)-2-trifluoromethyl-1*H*-inden-1-ol (**3cA**) S15

^{13}C NMR spectrum of 3-(4-Methoxyphenyl)-2-trifluoromethyl-1*H*-inden-1-ol (**3cA**) S15

^{19}F NMR spectrum of 3-(4-Methoxyphenyl)-2-trifluoromethyl-1*H*-inden-1-ol (**3cA**) S16

^{1}H NMR spectrum of 3-(4-Biphenyl)-2-trifluoromethyl-1*H*-inden-1-ol (**3eA**) S17

^{13}C NMR spectrum of 3-(4-Biphenyl)-2-trifluoromethyl-1*H*-inden-1-ol (**3eA**) S17

^{19}F NMR spectrum of 3-(4-Biphenyl)-2-trifluoromethyl-1*H*-inden-1-ol (**3eA**) S18

^{1}H NMR spectrum of 3-(1-Naphthyl)-2-trifluoromethyl-1*H*-inden-1-ol (**3fA**) S19

^{13}C NMR spectrum of 3-(1-Naphthyl)-2-trifluoromethyl-1*H*-inden-1-ol (**3fA**) S19

^{19}F NMR spectrum of 3-(1-Naphthyl)-2-trifluoromethyl-1*H*-inden-1-ol (**3fA**) S20

^{1}H NMR spectrum of 3-(3-Chlorophenyl)-2-trifluoromethyl-1*H*-inden-1-ol (**3gA**) S21

^{13}C NMR spectrum of 3-(3-Chlorophenyl)-2-trifluoromethyl-1*H*-inden-1-ol (**3gA**) S21

^{19}F NMR spectrum of 3-(3-Chlorophenyl)-2-trifluoromethyl-1*H*-inden-1-ol (**3gA**) S22

^{1}H NMR spectrum of 3-(4-Chlorophenyl)-2-difluoromethyl-1*H*-inden-1-ol (**3hA**) S23

^{13}C NMR spectrum of 3-(4-Chlorophenyl)-2-difluoromethyl-1*H*-inden-1-ol (**3hA**) S23

^{19}F NMR spectrum of 3-(4-Chlorophenyl)-2-difluoromethyl-1*H*-inden-1-ol (**3hA**) S24

^{1}H NMR spectrum of 3-(4-Chlorophenyl)-2-nonafluorobutyl-1*H*-inden-1-ol (**3iA**) S25

^{13}C NMR spectrum of 3-(4-Chlorophenyl)-2-nonafluorobutyl-1*H*-inden-1-ol (**3iA**) S25

^{19}F NMR spectrum of 3-(4-Chlorophenyl)-2-nonafluorobutyl-1*H*-inden-1-ol (**3iA**) S26

^{1}H NMR spectrum of 3-(4-Chlorophenyl)-6-fluoro-2-trifluoromethyl-1*H*-inden-1-ol (**3aB**) S27

^{13}C NMR spectrum of 3-(4-Chlorophenyl)-6-fluoro-2-trifluoromethyl-1*H*-inden-1-ol (**3aB**) S27

^{19}F NMR spectrum of 3-(4-Chlorophenyl)-6-fluoro-2-trifluoromethyl-1*H*-inden-1-ol (**3aB**) S28

^{1}H NMR spectrum of 6-Chloro-3-(4-Chlorophenyl)-2-trifluoromethyl-1*H*-inden-1-ol (**3aC**) S29

^{13}C NMR spectrum of 6-Chloro-3-(4-Chlorophenyl)-2-trifluoromethyl-1*H*-inden-1-ol (**3aC**) S29

^{19}F NMR spectrum of 6-Chloro-3-(4-Chlorophenyl)-2-trifluoromethyl-1*H*-inden-1-ol (**3aC**) S30

^{1}H NMR spectrum of 3-(4-Chlorophenyl)-6-methoxy-2-trifluoromethyl-1*H*-inden-1-ol (**3aD**) S31

^{13}C NMR spectrum of 3-(4-Chlorophenyl)-6-methoxy-2-trifluoromethyl-1*H*-inden-1-ol (**3aD**) S31

^{19}F NMR spectrum of 3-(4-Chlorophenyl)-6-methoxy-2-trifluoromethyl-1*H*-inden-1-ol (**3aD**) S32

^{1}H NMR spectrum of 6-benzyloxy-3-(4-Chlorophenyl)-2-trifluoromethyl-1*H*-inden-1-ol (**3aE**) S33

^{13}C NMR spectrum of 6-benzyloxy-3-(4-Chlorophenyl)-2-trifluoromethyl-1*H*-inden-1-ol (**3aE**) S33

^{19}F NMR spectrum of 6-benzyloxy-3-(4-Chlorophenyl)-2-trifluoromethyl-1*H*-inden-1-ol (**3aE**) S34

^{1}H NMR spectrum of 3-(4-Chlorophenyl)-5-fluoro-2-trifluoromethyl-1*H*-inden-1-ol (**3aF**) S35

^{13}C NMR spectrum of 3-(4-Chlorophenyl)-5-fluoro-2-trifluoromethyl-1*H*-inden-1-ol (**3aF**) S35

^{19}F NMR spectrum of 3-(4-Chlorophenyl)-5-fluoro-2-trifluoromethyl-1*H*-inden-1-ol (**3aF**) S36

^{1}H NMR spectrum of 3-(4-Chlorophenyl)-7-fluoro-2-trifluoromethyl-1*H*-inden-1-ol (**3aG**) S37

^{13}C NMR spectrum of 3-(4-Chlorophenyl)-7-fluoro-2-trifluoromethyl-1*H*-inden-1-ol (**3aG**) S37

^{19}F NMR spectrum of 3-(4-Chlorophenyl)-7-fluoro-2-trifluoromethyl-1*H*-inden-1-ol (**3aG**) S38

^{1}H NMR spectrum of 2-(4-Chlorophenyl)-3-trifluoromethyl-2,3-dihydro-1*H*-indan-1-one (**5aA**) S39

^{13}C NMR spectrum of 2-(4-Chlorophenyl)-3-trifluoromethyl-2,3-dihydro-1*H*-indan-1-one (**5aA**) S39

^{19}F NMR spectrum of 2-(4-Chlorophenyl)-3-trifluoromethyl-2,3-dihydro-1*H*-indan-1-one (**5aA**) S40

^{1}H NMR spectrum of 3-(4-Chlorophenyl)-2-trifluoromethyl-inden-1-one (**6**) S41

^{13}C NMR spectrum of 3-(4-Chlorophenyl)-2-trifluoromethyl-inden-1-one (**6**) S41

^{19}F NMR spectrum of 3-(4-Chlorophenyl)-2-trifluoromethyl-inden-1-one (**6**) S42

^{1}H NMR spectrum of 3-(4-Chlorophenyl)-2-trifluoromethyl-2,3-dihydro-1*H*-indan-1-one (**7**) S43

^{13}C NMR spectrum of 3-(4-Chlorophenyl)-2-trifluoromethyl-2,3-dihydro-1*H*-indan-1-one (**7**) S43

^{19}F NMR spectrum of 3-(4-Chlorophenyl)-2-trifluoromethyl-2,3-dihydro-1*H*-indan-1-one (**7**) S44

HMBC spectrum of 2-(4-Chlorophenyl)-3-trifluoromethyl-2,3-dihydro-1*H*-indan-1-one (**5aA**) S45

HMBC spectrum of 3-(4-Chlorophenyl)-2-trifluoromethyl-2,3-dihydro-1*H*-indan-1-one (**7**) S45

5. References S46

1. General informations

^1H and ^{13}C NMR spectra were obtained using an AVANCE III 400 NMR spectrometer (^1H: 400 MHz and ^{13}C: 100 MHz) in chloroform-*d* ($CDCl_3$) (Bruker, Germany), and the chemical shifts are reported in parts per million (ppm) based on the residual proton signal of the NMR solvent. ^{19}F NMR (376MHz) spectra were obtained using AVANCE III 400 NMR spectrometer in $CDCl_3$ with $CFCl_3$ (δ_F = 0 ppm) as an internal standard (Bruker, Germany). The Bruker AVANCE III 400 NMR spectrometer was used for determining the yield of the products with trifluoromethylbenzene ($CF_3C_6H_5$) or hexafluorobenzene (C_6F_6) as internal references. Infrared spectra (IR) were taken on a JASCO FT/IR 4100 type A spectrometer as a film on a NaCl film or KBr plate; all spectra are reported in wavenumbers (cm^{-1}). High-resolution mass spectra were recorded on a JMS-700MS spectrometer (JEOL, Japan) using the fast-atom bombardment (FAB) method.

All reactions were carried out using dried glassware with a magnetic stir bar and routinely monitored by ^{19}F NMR spectroscopy or thin-layer chromatography (TLC). All chemicals were of reagent grade and, if necessary, purified in the usual manner prior to use. Fluoroalkylated alkynes used in this research were prepared according to the literatures [1, 2]. Column chromatography was carried out on silica gel (Wako gel® 60N, 38-100 μm) and TLC analysis was performed on silica gel TLC plates (Merck, Silica gel $60F_{254}$).

2. Cobalt-catalyzed [2 + 3] cycloaddition and characterization of products

2-1. *Typical procedure.*

In a 30 mL two-necked round bottomed-flask, equipped with a magnetic stir bar, were placed fluoroalkylated alkyne **1a** (0.0818 g, 0.40 mmol), 2-formylphenylboronic acid (**2A**) (0.1200 g, 0.80 mmol), 1,3-bis(diphenylphosphino)propane (0.0165 g, 40 μmol), and $Co(acac)_2 \cdot 2H_2O$ (0.0119 g, 41 μmol) in acetonitrile (1.2 mL)/Isopropyl alcohol (0.4 mL), and the resulting mixture was stirred at reflux temperature in an oil bath. After 18 h, the reaction mixture was cooled to room temperature. Subsequently, the reaction mixture was subjected to flash column chromatography using silica gel as stationary phase and acetone as mobile phase. After removal of the solvent from the eluent under reduced pressure, the residue was purified by silica gel column chromatography (Hexane/AcOEt = 5:1) to give the corresponding 3-(4-chlorophenyl)-2-trifluoromethyl-1*H*-inden-1-ol (**3aA**) (0.085 g, 0.27 mmol).

2-2. *Characterizations of fluoroalkylated indenols*

2-2.1. **3-(4-Chlorophenyl)-2-trifluoromethyl-1*H*-inden-1-ol (3aA)**

Yield: 68%; white solid; M.p. 104.5–106.5 °C; eluent of the column chromatography: Hexane/EtOAc = 5/1; ^{1}H NMR ($CDCl_3$): δ 2.07 (br s, 1H, O*H*), 5.55 (s, 1H, C*H*), 7.12 (d, J = 7.4 Hz, 1H, Ar*H*), 7.32–7.38 (m, 3H, Ar*H*), 7.41 (td, J = 7.4, 0.97 Hz, 1H, Ar*H*), 7.47 (d, J = 8.6 Hz, 2H, Ar*H*), 7.64 (d, J = 7.4 Hz, 1H, Ar*H*); ^{13}C NMR ($CDCl_3$): δ 76.1 (m, *C*–OH), 122.6 (Ar), 123.3 (q, J = 269.5 Hz, *C*F_3), 124.3 (Ar), 129.0 (Ar), 129.1 (Ar), 129.3 (Ar), 129.9 (m, Ar), 130.4 (Ar), 131.8 (q, J = 30.9 Hz, *C*–CF_3), 135.3 (Ar), 141.1 (Ar), 144.1 (Ar), 148.0 (q, J = 4.5 Hz, CF_3–C=*C*); ^{19}F NMR ($CDCl_3$, $CFCl_3$): δ –56.56 (s, 3F); IR (KBr) 3277, 1634, 1493, 1460, 1401, 1357, 1303, 1287, 1253, 1189, 1139, 1094, 1045, 1014, 840 , 822, 779 , 742 cm^{-1}; HRMS (FAB): calcd for [M^+] $C_{16}H_{10}ClF_3O$: 310.0372, Found: 310.0382.

2-2.2. **3-[4-(*t*-Butyl)phenyl]-2-trifluoromethyl-1*H*-inden-1-ol (3bA)**

Yield: 69%; yellow solid; M.p. 45.4–47.3 °C; eluent of the column chromatography: Hexane/EtOAc = 5/1; ^{1}H NMR ($CDCl_3$): δ 1.38 (s, 9H, C–(CH_3)$_3$), 2.10 (d, J = 7.6 Hz, 1H, O*H*), 5.55 (d, J = 7.6 Hz, 1H, C*H*), 7.22 (d, J = 7.3 Hz, 1H, Ar*H*), 7.31-7.41 (m, 4H, Ar*H*), 7.49 (d, J = 8.4 Hz, 2H, Ar*H*), 7.63 (d, J = 7.3 Hz, 1H, Ar*H*); ^{13}C NMR ($CDCl_3$): δ 31.4 ((*C*H_3)$_3$), 34.9 (*C*–(CH_3)$_3$), 76.2 (m, *C*–OH), 123.0 (Ar), 123.5 (q, J = 267.3 Hz, *C*F_3), 124.1 (Ar), 125.5 (Ar), 128.2 (m, Ar), 128.8 (Ar), 128.9 (Ar), 129.1 (Ar), 130.8 (q, J = 31.0 Hz, *C*–CF_3), 141.6 (Ar), 144.2 (Ar), 149.3 (q, J = 4.5 Hz, CF_3–C=*C*), 152.3 (Ar); ^{19}F NMR ($CDCl_3$, $CFCl_3$): δ –56.33 (s, 3F); IR (KBr) 3309, 2965, 1634, 1462, 1362, 1257, 1192, 1145, 1112, 1079, 1035, 842, 824, 783, 748, 736, 705 cm^{-1}; HRMS (FAB): calcd for [M^+] $C_{20}H_{19}F_3O$: 332.1388, Found: 332.1397.

2-2.3. **3-(4-Methoxyphenyl)-2-trifluoromethyl-1*H*-inden-1-ol (3cA)**

Yield: 65%; yellow solid; M.p. 100.5–102.4 °C; eluent of the column chromatography: Hexane/EtOAc = 5/1; ^{1}H NMR ($CDCl_3$): δ 2.02 (br s, 1H, O*H*), 3.87 (s, 3H, CH_3), 5.54 (s, 1H, C*H*), 7.01 (d, *J* = 8.8 Hz, 2H, Ar*H*), 7.20 (d, *J* = 7.3 Hz, 1H, Ar*H*), 7.31–7.42 (m, 4H, Ar*H*), 7.63 (d, *J* = 7.3 Hz, 1H, Ar*H*); ^{13}C NMR ($CDCl_3$): δ 55.4 (OCH_3), 76.1 (m, *C*–OH), 114.1 (Ar), 122.8 (Ar), 123.6 (q, *J* = 270.7 Hz, CF_3), 124.1 (Ar), 124.2 (Ar), 128.8 (Ar), 129.2 (Ar), 129.9 (m, Ar), 130.6 (q, *J* = 31.0 Hz, *C*–CF_3), 141.6 (Ar), 144.3 (Ar), 149.0 (q, *J* = 4.4 Hz, CF_3–C=*C*), 160.3 (Ar); ^{19}F NMR ($CDCl_3$, $CFCl_3$): δ –56.32 (s, 3F); IR (KBr) 3340, 2840, 1631, 1612, 1463, 1441, 1309, 1300, 1282, 842, 824, 798, 775, 717 cm^{-1}; HRMS (FAB): calcd for [M^{+}] $C_{17}H_{13}F_3O_2$: 306.0868, Found: 306.0875.

2-2.4. **3-(4-Biphenyl)-2-trifluoromethyl-1*H*-inden-1-ol (3eA)**

Yield: 63%; white solid; M.p. 172.0–174.5 °C; eluent of the column chromatography: Hexane/EtOAc = 5/1; ^{1}H NMR ($CDCl_3$): δ 2.07 (br s, 1H, O*H*), 5.59 (s, 1H, C*H*), 7.24 (d, *J* = 7.4 Hz, 1H, Ar*H*), 7.33–7.53 (m, 7H, Ar*H*), 7.64–7.69 (m, 3H, Ar*H*), 7.72 (d, *J* = 8.4 Hz, 2H); ^{13}C NMR ($CDCl_3$): δ 76.2 (m, *C*–OH), 122.9 (Ar), 123.5 (q, *J* = 271.0 Hz, CF_3), 124.2 (Ar), 127.27 (Ar), 127.29 (Ar), 127.9 (Ar), 128.98 (Ar), 129.04 (Ar), 129.3 (Ar), 130.8 (Ar), 131.3 (q, *J* = 30.9 Hz, *C*–CF_3), 140.5 (Ar), 141.5 (Ar), 142.0 (Ar), 144.2 (Ar), 149.0 (q, *J* = 4.6 Hz, CF_3–C=*C*), the signal of one carbon was overlapped with other signals; ^{19}F NMR ($CDCl_3$, $CFCl_3$): δ –56.37 (s, 3F); IR (KBr): 3539, 1630, 1460, 1381, 1360, 1254, 1237, 1195, 1158, 1143, 1104, 1080, 1034, 844, 787 cm^{-1}; HRMS (FAB): calcd for [M^{+}] $C_{22}H_{15}F_3O$: 352.1075, Found: 352.1083.

2-2.5. **3-(1-Naphthyl)-2-trifluoromethyl-1*H*-inden-1-ol (3fA)**

Yield: 28%; yellow solid; M.p. 111.5–113.8 °C; eluent of the column chromatography: Hexane/EtOAc = 5/1; (atropisomer 1 and 2): ^{1}H NMR ($CDCl_3$): δ 2.18 (br s, 1H, O*H*), 2.29 (br s, 1H, O*H*), 5.69 (s, 1H, C*H*), 5.75 (s, 1H, C*H*), 6.76 (t, *J* = 8.0 Hz, 2H, Ar*H*), 7.18–7.26 (m, 2H, Ar*H*), 7.35–7.80 (m, 14H, Ar*H*), 7.90–7.98 (m, 4H, Ar*H*); ^{13}C NMR ($CDCl_3$): δ 76.15 (m, *C*–OH), 76.21 (m, *C*–OH), 123.1 (Ar), 123.32 (Ar), 123.33 (q, *J* = 270.8 Hz, CF_3), 123.4 (q, *J* = 270.0 Hz, CF_3), 124.0 (Ar), 124.1 (Ar), 125.3 (Ar), 125.5 (Ar), 125.7 (Ar), 126.01 (Ar), 126.02 (Ar), 126.38 (Ar), 126.41 (Ar), 126.5 (Ar), 126.6 (Ar), 128.56 (Ar), 128.60 (Ar), 128.96 (Ar), 128.99 (Ar), 129.25 (Ar), 129.31 (Ar), 129.35 (Ar), 129.39 (Ar), 129.7 (Ar), 130.0 (Ar), 130.9 (Ar), 131.2 (Ar), 133.3 (q, *J* = 31.0 Hz, *C*–CF_3), 133.4 (Ar), 133.6 (Ar), 133.7 (q, *J* = 31.2 Hz, *C*–CF_3), 142.08 (Ar), 142.14 (Ar), 143.7 (Ar), 144.0 (Ar), 148.4 (q, *J* = 4.7 Hz, CF_3–C=*C*), 148.8 (q, *J* = 4.7 Hz, CF_3–C=*C*), the signal of one carbon was overlapped with other signals; ^{19}F NMR ($CDCl_3$, $CFCl_3$): δ –58.23 (s, 3F), –57.59 (s, 3F); IR (KBr): 3326, 1643, 1460, 1403, 1361, 1270, 1250, 1220, 1193, 1170, 1147, 1121, 1074, 1050, 1031,

834, 799, 786, 774, 742, 717 cm^{-1}; HRMS (FAB): calcd for [M$^+$] $C_{20}H_{13}F_3O$: 326.0918, Found: 326.0923.

2-2.6. **3-(3-Chlorophenyl)-2-trifluoromethyl-1*H*-inden-1-ol (3gA)**

Yield: 72%; yellow oil; eluent of the column chromatography: Hexane/EtOAc = 5/1; ^{1}H NMR ($CDCl_3$): δ 2.08 (d, J = 7.2 Hz, 1H, O*H*), 5.56 (d, J = 7.2 Hz, 1H, C*H*), 7.12 (d, J = 7.5 Hz, 1H, Ar*H*), 7.25–7.50 (m, 6H, Ar*H*), 7.64 (d, J = 7.4 Hz, 1H, Ar*H*); ^{13}C NMR ($CDCl_3$): δ 75.9 (*C*–OH), 122.5 (Ar), 123.1 (q, J = 271.1 Hz, *C*F_3), 124.2 (Ar), 126.7 (Ar), 128.3 (Ar), 129.1 (Ar), 129.3 (Ar), 129.9 (Ar), 132.0 (q, J = 31.3 Hz, *C*–CF_3), 133.7 (Ar), 134.5 (Ar), 140.9 (Ar), 144.0 (Ar), 147.6 (q, J = 4.3 Hz, CF_3–C=*C*), the signal of one carbon was overlapped with other signals; ^{19}F NMR ($CDCl_3$, $CFCl_3$): δ –56.63 (s, 3F); IR (neat): 3338, 3072, 2888, 2681, 1638, 1588, 1480, 1195, 1080, 786, 712 cm^{-1}; HRMS (FAB): calcd for [M$^+$] $C_{16}H_{10}ClF_3O$: 310.0372, Found: 310.0378.

2-2.7. **3-(4-Chlorophenyl)-2-difluoromethyl-1*H*-inden-1-ol (3hA)**

Yield: 43%; yellow solid; M.p. 91.2–92.2 °C; eluent of the column chromatography: Hexane/EtOAc = 5/1; ^{1}H NMR ($CDCl_3$): δ 2.28 (d, J = 6.6 Hz, 1H, O*H*), 5.62 (d, J = 6.6 Hz, 1H, C*H*), 6.46 (t, J = 54.4 Hz, 1H, CF_2*H*), 7.21 (d, J = 7.3 Hz, 1H, Ar*H*), 7.31–7.42 (m, 4H, Ar*H*), 7.50 (d, J = 8.5 Hz, 2H, Ar*H*), 7.64 (d, J = 7.3 Hz, 1H, Ar*H*); ^{13}C NMR ($CDCl_3$): δ 75.3 (*C*–OH), 112.9 (t, J = 231.5 Hz, *C*HF_2), 121.9 (Ar), 124.4 (Ar), 128.6 (Ar), 129.0 (Ar), 129.3 (Ar), 130.08 (Ar), 130.12 (Ar), 135.5 (Ar), 135.7 (t, J = 22.7 Hz, *C*–CF_2H), 140.8 (Ar), 144.7 (Ar), 147.1 (t, J = 10.4 Hz, CF_2H–C=*C*); ^{19}F NMR ($CDCl_3$, $CFCl_3$): δ –111.51 (dd, J = 316.5, 54.4 Hz, 1F), –110.50 (dd, J = 316.5, 54.4 Hz, 1F); IR (KBr): 3311, 2341, 1916, 1625, 1491, 1375, 1170, 1113, 1081, 1048, 1014, 941, 930, 835, 824, 793, 768, 744, 726, 715 cm^{-1}; HRMS (FAB): calcd for [M$^+$] $C_{16}H_{11}ClF_2O$: 292.0466, Found: 292.0467.

2-2.8. **3-(4-Chlorophenyl)-2-nonafluorobutyl-1*H*-inden-1-ol (3iA)**

Yield: 43%; yellow solid; M.p. 95.2–96.7 °C; eluent of the column chromatography: Hexane/EtOAc = 5/1; ^{1}H NMR ($CDCl_3$): δ 2.15 (br s, 1H, O*H*), 5.59 (s, 1H, C*H*), 6.97 (d, J = 7.5 Hz 1H, Ar), 7.22–7.30 (m, 2H, Ar*H*), 7.33 (td, J = 7.6, 0.85 Hz, 1H, Ar*H*), 7.38–7.46 (m, 3H, Ar*H*), 7.64 (dm, J = 7.8 Hz, 1H, Ar*H*); ^{13}C NMR ($CDCl_3$): δ 76.8 (*C*–OH), 108.0–123.0 (m, 3C, CF_2–*C*$_3F_7$), 116.4 (tt, J = 257.2, 33.9 Hz, *C*F_2–C_3F_7), 122.7 (Ar), 124.1 (Ar), 128.7 (Ar), 129.3 (Ar), 129.4 (Ar), 129.8 (br s, Ar), 130.4 (t, J = 22.3 Hz, 1C), 130.8 (Ar), 135.0 (Ar), 141.7 (Ar), 144.4 (Ar), 151.6 (t, J = 5.7 Hz, C_4F_9H–C=*C*); ^{19}F NMR ($CDCl_3$, $CFCl_3$): δ –126.38 to –126.25 (m, 2F), –122.00 to –121.78 (m, 2F), –104.07 (quin., J = 13.3 Hz, 2F), –81.43 (t, J = 9.7 Hz, 3F); IR (KBr): 3436, 1625, 1491, 1461,

1415, 1354, 1334, 1294, 1231, 1199, 1131, 1091, 1065, 1015, 870, 842, 831, 795, 768, 744, 732 cm^{-1}; HRMS (FAB): calcd for [M$^+$] $C_{19}H_{10}ClF_9O$: 460.0276, Found: 460.0266.

2-2.9. **3-(4-Chlorophenyl)-6-fluoro-2-trifluoromethyl-1*H*-inden-1-ol (3aB)**

Yield: 36%; yellow solid; M.p. 108.2–109.7 °C; eluent of the column chromatography: Hexane/EtOAc = 5/1; ^{1}H NMR ($CDCl_3$): δ 2.20 (d, J = 6.6 Hz, 1H, O*H*), 5.52 (d, J = 6.6 Hz, 1H, C*H*), 6.99–7.09 (m, 2H, Ar*H*), 7.31–7.37 (m, 3H, Ar*H*), 7.47 (d, J = 8.6 Hz, 2H, Ar*H*); ^{13}C NMR ($CDCl_3$): δ 75.6 (*C*–OH), 112.6 (d, J = 24.2 Hz, Ar), 116.1 (d, J = 23.0 Hz, Ar), 123.1 (q, J = 270.9 Hz, *C*F$_3$), 123.9 (d, J = 8.8 Hz, Ar), 129.1 (Ar), 129.8 (Ar), 130.1 (Ar), 131.5 (m, *C*–CF$_3$), 135.5 (Ar), 136.9 (d, J = 2.2 Hz, Ar), 146.5 (d, J = 8.5 Hz, CF$_3$–C=*C*), 147.4 (d, J = 3.8 Hz, Ar), 168.8 (d, J = 250.1 Hz, Ar); ^{19}F NMR ($CDCl_3$, $CFCl_3$): δ –111.89 to –111.81 (m, 1F), –56.64 (s, 3F); IR (KBr): 3337, 1634, 1614, 1604, 1591, 1492, 1455, 1434, 1402, 1310, 1266, 1013, 906, 882, 864, 835, 762, 739, 716 cm^{-1}; HRMS (FAB): calcd for [M$^+$] $C_{16}H_9ClF_4O$: 328.0278, Found: 328.0278.

2-2.10. **6-Chloro-3-(4-chlorophenyl)-2-trifluoromethyl-1*H*-inden-1-ol (3aC)**

Yield: 26%; yellow solid; M.p. 136.0–138.0 °C; eluent of the column chromatography: Hexane/EtOAc = 5/1; ^{1}H NMR ($CDCl_3$): δ 2.36 (d, J = 7.5 Hz, 1H, O*H*), 5.53 (d, J = 7.5 Hz, 1H, C*H*), 7.04 (d, J = 8.1 Hz, 1H, Ar*H*), 7.29–7.36 (m, 3H, Ar*H*), 7.47 (d, J = 8.6 Hz, 2H, Ar*H*), 7.61 (d, J = 1.8 Hz, 1H, Ar*H*); ^{13}C NMR ($CDCl_3$): δ 75.7 (m, *C*–OH), 123.0 (q, J = 271.1 Hz, *C*F$_3$), 123.5 (Ar), 125.0 (Ar), 129.1 (Ar), 129.5 (Ar), 129.8 (m, Ar), 129.9 (Ar), 131.9 (q, J = 31.5 Hz, *C*–CF$_3$), 135.5 (Ar), 135.6 (Ar), 139.5 (Ar), 145.7 (Ar), 147.3 (q, J = 4.4 Hz, CF$_3$–C=*C*); ^{19}F NMR ($CDCl_3$, $CFCl_3$): δ –56.69 (s, 3F); IR (KBr): 3224, 1637, 1580, 1492, 1401, 1358, 1292, 1250, 1193, 1149, 1120, 1088, 1064, 1043, 1013, 933, 901, 870, 824, 779, 704 cm^{-1}; HRMS (FAB): calcd for [M$^+$] $C_{16}H_9Cl_2F_3O$: 343.9983, Found: 343.9982.

2-2.11. **3-(4-Chlorophenyl)-6-methoxy-2-trifluoromethyl-1*H*-inden-1-ol (3aD)**

Yield: 49%; yellow solid; M.p. 133.0–134.0 °C; eluent of the column chromatography: Hexane/EtOAc = 4/1; ^{1}H NMR ($CDCl_3$): δ 2.20 (d, J = 7.2 Hz, 1H, O*H*), 3.85 (s, 3H, C*H$_3$*), 5.48 (d, J = 7.2 Hz, 1H, C*H*), 6.83 (dd, J = 8.4, 2.3 Hz, 1H, Ar*H*), 7.01 (d, J = 8.4 Hz, 1H, Ar*H*), 7.20 (d, J = 2.3 Hz, 1H, Ar*H*), 7.34 (d, J = 8.5 Hz, 2H, Ar*H*), 7.45 (d, J = 8.5 Hz, 2H, Ar*H*); ^{13}C NMR ($CDCl_3$): δ 55.8 (*C*H$_3$), 75.8 (*C*–OH), 110.8 (Ar), 114.4 (Ar), 123.4 (q, J = 270.6 Hz, *C*F$_3$), 123.5 (Ar), 128.9 (Ar), 129.5 (q, J = 31.2 Hz, *C*–CF$_3$), 129.8 (Ar), 130.6 (Ar), 133.6 (Ar), 135.2 (Ar), 146.3 (Ar), 148.0 (q, J = 4.5 Hz, CF$_3$–C=*C*), 161.2 (Ar); ^{19}F NMR ($CDCl_3$, $CFCl_3$): δ –56.22 (s, 3F); IR (KBr): 3406, 3064, 2946, 2846, 2681, 2319, 1623, 1490, 1360, 1273, 1144, 1041, 813, 764 cm^{-1}; HRMS (FAB): calcd for [M$^+$] $C_{17}H_{12}ClF_3O_2$: 340.0478, Found: 340.0475.

2-2.12. **6-Benzyloxy-3-(4-chlorophenyl)-2-trifluoromethyl-1*H*-inden-1-ol (3aE)**

Yield: 41%; white solid; M.p. 105.2–106.0 °C; eluent of the column chromatography: Hexane/EtOAc = 5/1; ^{1}H NMR ($CDCl_3$): δ 2.08 (br s, 1H, O*H*), 5.13 (s, 2H, CH_2), 5.49 (s, 1H, C*H*), 6.91 (dd, J = 8.4, 2.4 Hz, 1H, Ar*H*), 7.02 (d, J = 8.4 Hz, 1H, Ar*H*), 7.24–7.48 (m, 10H, Ar*H*); ^{13}C NMR ($CDCl_3$): δ 70.4 (O–*C*H_2), 75.7 (*C*–OH), 111.7 (Ar), 115.3 (Ar), 123.4 (q, J = 270.8 Hz, *C*F_3), 127.4 (Ar), 127.5 (Ar), 128.3 (Ar), 128.8 (Ar), 128.9 (Ar), 129.6 (q, J = 31.7 Hz, *C*–CF_3), 129.8 (Ar), 130.6 (Ar), 133.8 (Ar), 135.1 (Ar), 136.5 (Ar), 146.3 (Ar), 147.9 (q, J = 4.5 Hz, CF_3–C=*C*), 160.2 (Ar); ^{19}F NMR ($CDCl_3$, $CFCl_3$): δ –56.27 (s, 3F); IR (KBr): 3359, 3034, 2872, 1905, 1609, 1361, 1236, 1158, 1077, 824, 737 cm^{-1}; HRMS (FAB): calcd for [M^{+}] $C_{23}H_{16}ClF_3O_2$: 416.0791, Found: 416.0802.

2-2.13. **3-(4-Chlorophenyl)-5-fluoro-2-trifluoromethyl-1*H*-inden-1-ol (3aF)**

Yield: 48%; white solid; M.p. 110.0–110.8 °C; eluent of the column chromatography: Hexane/EtOAc = 5/1; ^{1}H NMR ($CDCl_3$): δ 2.10 (br s, 1H, O*H*), 5.53 (s, 1H, C*H*), 6.81 (dd, J = 8.4, 2.3 Hz, 1H, Ar*H*), 7.08 (td, J = 8.4, 2.3 Hz, 1H, Ar*H*), 7.33 (d, J = 8.4 Hz, 2H, Ar*H*), 7.48 (d, J = 8.4 Hz, 2H, Ar*H*), 7.58 (dd, J = 8.4, 4.9 Hz, 1H, Ar*H*); ^{13}C NMR ($CDCl_3$): δ 75.3 (*C*–OH), 110.2 (d, J = 24.6 Hz, Ar), 115.7 (d, J = 23.0 Hz, Ar), 122.9 (q, J = 271.2 Hz, *C*F_3), 125.6 (d, J = 9.1 Hz, Ar), 129.1 (Ar), 129.7 (Ar), 133.5 (q, J = 31.3 Hz, *C*–CF_3), 135.6 (Ar), 139.5 (Ar), 143.2 (d, J = 8.6 Hz, Ar), 146.9–147.1 (m, 2C, CF_3–C=*C*, Ar), 163.8 (d, J = 247.2 Hz, Ar); ^{19}F NMR ($CDCl_3$, $CFCl_3$): δ –112.26 to –112.19 (m, 1F), –56.88 (s, 3F); IR (KBr): 3336, 3073, 2918, 1921, 1745, 1628, 1592, 1360, 1200, 1126, 820 cm^{-1}; HRMS (FAB): calcd for [M^{+}] $C_{16}H_9ClF_4O$: 328.0278, Found: 328.0275.

2-2.14. **3-(4-Chlorophenyl)-7-fluoro-2-trifluoromethyl-1*H*-inden-1-ol (3aG)**

Yield: 15%; yellow solid; M.p. 96.2–97.0 °C; eluent of the column chromatography: Hexane/EtOAc = 5/1; ^{1}H NMR ($CDCl_3$): δ 2.38 (d, J = 4.9 Hz, 1H, O*H*), 5.81 (d, J = 4.9 Hz, 1H, C*H*), 6.91 (d, J = 7.5 Hz, 1H, Ar*H*), 7.07 (t, J = 8.6 Hz, 1H, Ar*H*), 7.29–7.37 (m, 3H, Ar*H*), 7.47 (d, J = 8.6 Hz, 2H, Ar*H*); ^{13}C NMR ($CDCl_3$): δ 74.1 (*C*–OH), 116.7 (d, J = 20.6 Hz, Ar), 118.7 (d, J = 3.0 Hz, Ar), 122.8 (q, J = 271.3 Hz, *C*F_3), 128.8 (d, J = 16.0 Hz, Ar), 129.0 (Ar), 129.8 (d, J = 1.1 Hz, Ar), 129.9 (Ar), 131.6 (d, J = 7.3 Hz, Ar), 132.2 (qd, J = 31.7, 1.3 Hz, *C*–CF_3), 133.5 (Ar), 144.2 (d, J = 5.6 Hz, Ar), 147.5–147.8 (m, 1C, CF_3–C=*C*), 159.3 (d, J = 251.8 Hz, Ar); ^{19}F NMR ($CDCl_3$, $CFCl_3$): δ –119.95 to –119.88 (m, 1F), –56.88 (s, 3F); IR (KBr): 3330, 2928, 1938, 1920, 1624, 1598, 1476, 1360, 1251, 1193, 1091, 800 cm^{-1}; HRMS (FAB): calcd for [M^{+}] $C_{16}H_9ClF_4O$: 328.0278, Found: 328.0279.

2-2.15. ***trans*-2-(4-Chlorophenyl)-3-trifluoromethyl-2,3-dihydro-1*H*-indan-1-one (5aA)**

Yield: 32% (Reaction conditions: Entry 5 in Table 1); orange oil; eluent of the column chromatography: Hexane/EtOAc = 10/1; ^{1}H NMR ($CDCl_3$): δ 3.96 (d, J = 4.1 Hz, 1H, Ar–C*H*), 4.12 (qd, J = 8.6, 4.1 Hz, 1H, C*H*–CF_3), 7.09 (d, J = 8.5 Hz, 2H, Ar*H*), 7.32 (d, J = 8.5 Hz, 2H, Ar*H*), 7.61 (td, J = 7.0, 1.5, 1H, Ar*H*), 7.73–7.81 (m, 2H, Ar*H*); 7.90 (d, J = 7.7 Hz, 1H, Ar*H*); ^{13}C NMR ($CDCl_3$): δ 52.0 (q, J = 28.7 Hz, *C*–CF_3), 53.8 (m, *C*H–Ar), 125.2 (Ar) 126.3 (q, J = 278.9 Hz, *C*F_3), 127.0 (Ar), 129.47 (Ar), 129.49 (Ar), 130.3 (Ar), 134.0 (Ar), 136.07 (Ar), 136.10 (Ar), 136.7 (Ar), 146.1 (m, Ar), 201.7 (*C*=O); ^{19}F NMR ($CDCl_3$, $CFCl_3$): δ –69.87 (d, J = 8.6 Hz, 3F); IR (neat): 1730, 1606, 1590, 1494, 1465, 1362, 1294, 1262, 1254, 1198, 1159, 1118, 1094, 1015, 822, 761, 714 cm^{-1}; HRMS (FAB): calcd for $[M+H]^+$ $C_{16}H_{11}ClF_3O$: 311.0451, Found: 311.0461.

3. Synthesis of 2-fluoroalkylated indanone

3-1. *Typical procedure*

In a 30 mL two-necked round bottomed-flask, equipped with a magnetic stir bar, were placed 2-fluoroalkylated indenol **3aA** (0.169 g, 0.54 mmol) and MnO_2 (0.458 g, 5.3 mmol) in dichloromethane (20 mL), and the resulting mixture was stirred at 0 °C in an ice bath. After 0.5 h, the reaction mixture was percolated by cellaite with ethyl acetate. After removal of the solvent from the eluent under reduced pressure, the residue was purified by silica gel column chromatography (hexane/EtOAc = 5:1) to give the corresponding 3-(4-chlorophenyl)-2-trifluoromethylinden-1-one (**6**, 0.136 g, 0.44 mmol).

To a stirred solution of the **6** (0.136 g, 0.44 mmol) above under H_2 in MeOH (9.3 mL) was added 1 mol % of Pd/C (0.00500 g Pd/C, 4.7 μmol based on [Pd]) at room temperature, then the mixture was stirred at room temperature. After 14 h, the reaction mixture was subjected to flash column chromatography using silica gel as stationary phase and acetone as mobile phase. After removal of the solvent from the eluent under reduced pressure, the residue was purified by silica gel column chromatography (hexane/EtOAc = 10:1) to give the corresponding 3-(4-chlorophenyl)-2-trifluoromethyl-2,3-dihydro-1*H*-indan-1-one (**7**) (0.0793 g, 0.26 mmol).

3-2. *Characterization of 2-fluoroalkylated indenone and indanone*

3-2.1. **3-(4-Chlorophenyl)-2-trifluoromethylinden-1-one (6)**

Yield: 81%; yellow solid; eluent of the column chromatography: Hexane/EtOAc = 5/1; ^{1}H NMR ($CDCl_3$): δ 7.07–7.12 (m, 1H, Ar*H*), 7.40–7.47 (m, 4H, Ar*H*), 7.53 (d, J = 8.6 Hz, 2H, Ar*H*), 7.61–7.67 (m, 1H, Ar*H*); ^{13}C NMR ($CDCl_3$): δ 121.7 (q, J = 271.4 Hz, *C*F_3), 121.9 (q, J = 32.4 Hz, *C*–CF_3), 123.6 (Ar), 123.9 (Ar), 128.8 (Ar), 129.2 (Ar), 129.4 (m, Ar), 129.9 (m, Ar), 131.6 (Ar), 134.1 (Ar), 137.0 (Ar), 142.7 (Ar), 162.4 (q, J = 3.9 Hz, CF_3–C=*C*), 190.4 (*C*=O); ^{19}F NMR ($CDCl_3$, $CFCl_3$): δ

–57.96 (s, 3F); IR (KBr): 1720, 1618, 1591, 1488, 1456, 1401, 1360, 1230, 1196, 1177, 1143, 1119, 1092, 1011, 850, 833, 822, 768, 735, 706 cm^{-1}; HRMS (FAB): calcd for $[M+H]^+$ $C_{16}H_9ClF_3O$: 309.0294, Found: 309.0287.

3-2.2. ***trans*-3-(4-Chlorophenyl)-2-trifluoromethyl-2,3-dihydro-1*H*-indan-1-one (7)**

Yield: 58%; yellow oil; eluent of the column chromatography: Hexane/EtOAc = 10/1; ^{1}H NMR ($CDCl_3$): δ 3.32 (qd, J = 9.8, 4.7 Hz 1H, C*H*–CH–CF_3), 4.70 (d, J = 4.7 Hz, 1H, Ar–C*H*), 7.08 (d, J = 8.5 Hz, 2H, Ar*H*), 7.25 (dm, J = 7.6 Hz, 1H, Ar*H*); 7.33 (d, J = 8.5 Hz, 2H, Ar*H*), 7.50 (t, J = 7.6 Hz, 1H, Ar*H*), 7.66 (tm, J = 7.6 Hz, 1H, ArH), 7.88 (d, J = 7.6 Hz, 1H, Ar*H*); ^{13}C NMR ($CDCl_3$): δ 45.9 (m, *C*H–Ar), 59.6 (q, J = 26.2 Hz, *C*–CF_3), 124.6 (Ar), 124.8 (q, J = 279.5 Hz, *C*F_3), 126.9 (Ar), 129.1 (Ar), 129.46 (Ar), 129.54 (Ar), 133.9 (Ar), 135.7 (Ar), 136.5 (Ar), 140.0 (Ar), 155.0 (Ar), 195.9 (m, *C*=O); ^{19}F NMR ($CDCl_3$, $CFCl_3$): δ –67.15 (d, J = 9.8 Hz, 3F); IR (neat) 1731, 1493, 2973, 1360, 1322, 1293, 1257, 1216, 1200, 1161, 1114, 1014, 762, 753(m) cm^{-1}; HRMS (FAB): calcd for $[M+H]^+$ $C_{16}H_{11}ClF_3O$: 311.0451, Found: 311.0440.

4. Copies of ^{1}H, ^{13}C, ^{19}F NMR, and HMBC spectra for new compounds

^{1}H NMR spectrum of **3-(4-Chlorophenyl)-2-trifluoromethyl-1*H*-inden-1-ol (3aA)**

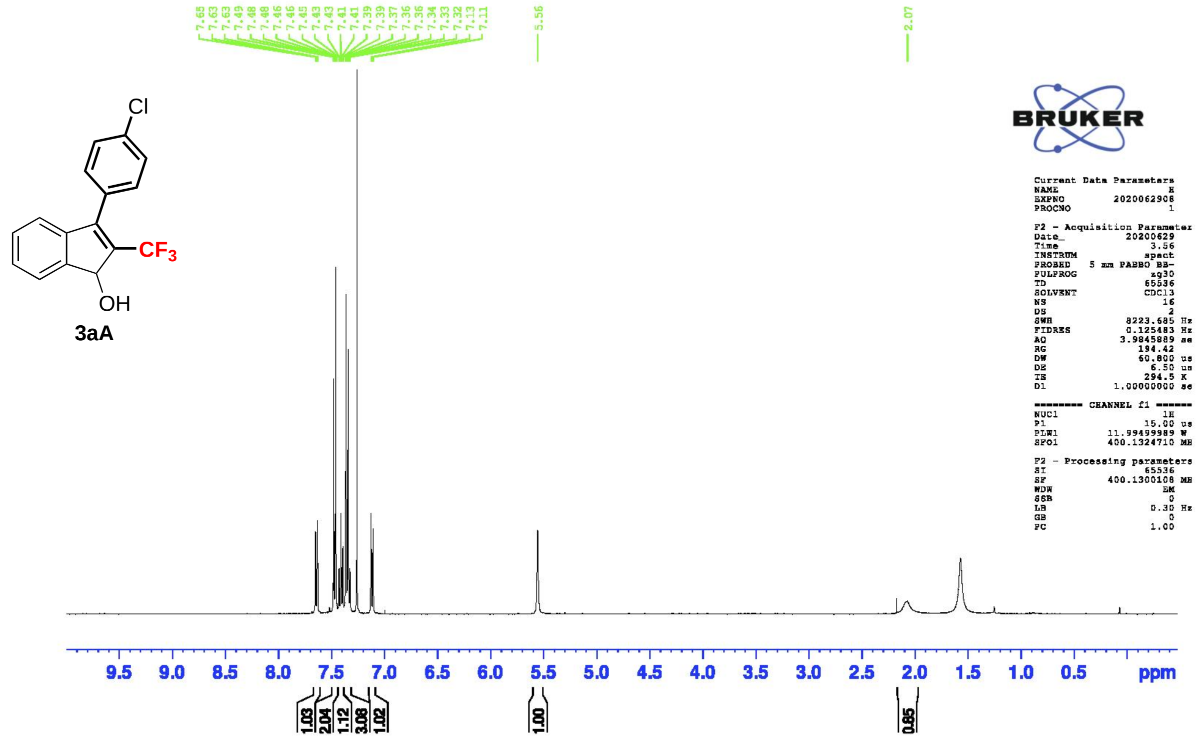

^{13}C NMR spectrum of **3-(4-Chlorophenyl)-2-trifluoromethyl-1*H*-inden-1-ol (3aA)**

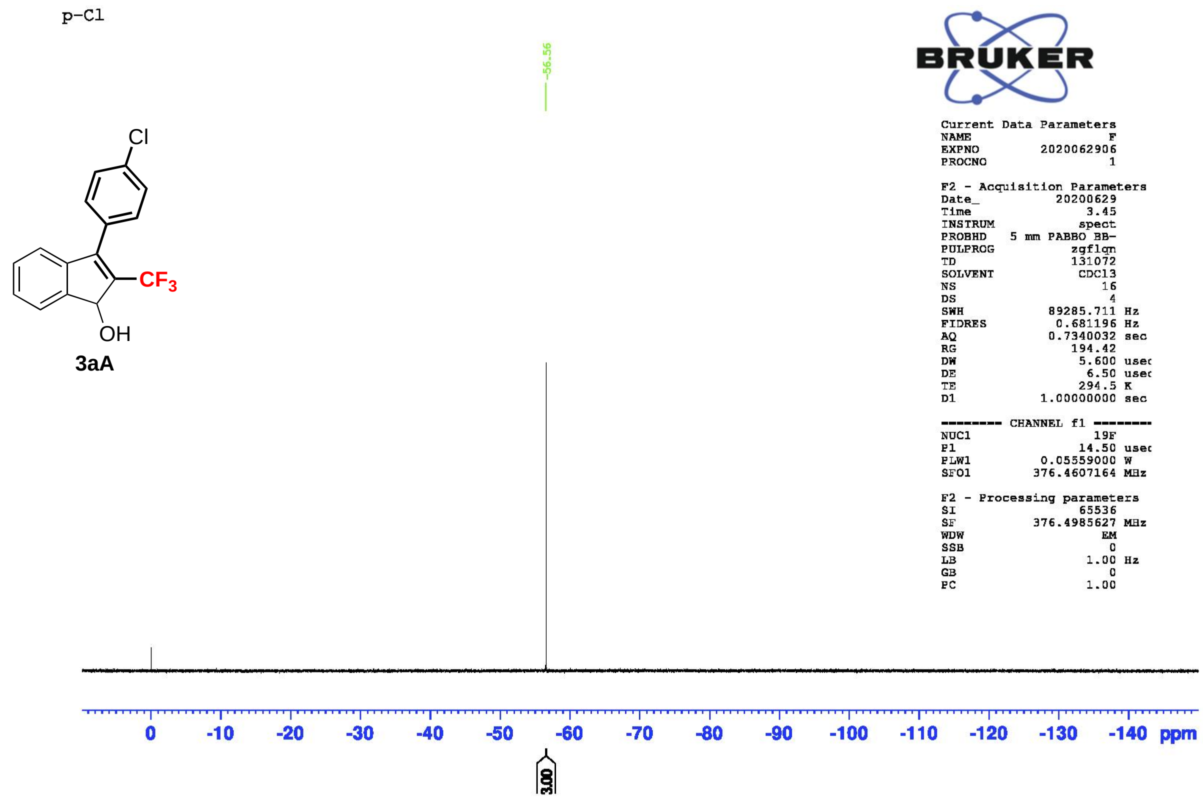

^{19}F NMR spectrum of **3-(4-Chlorophenyl)-2-trifluoromethyl-1*H*-inden-1-ol (3aA)**

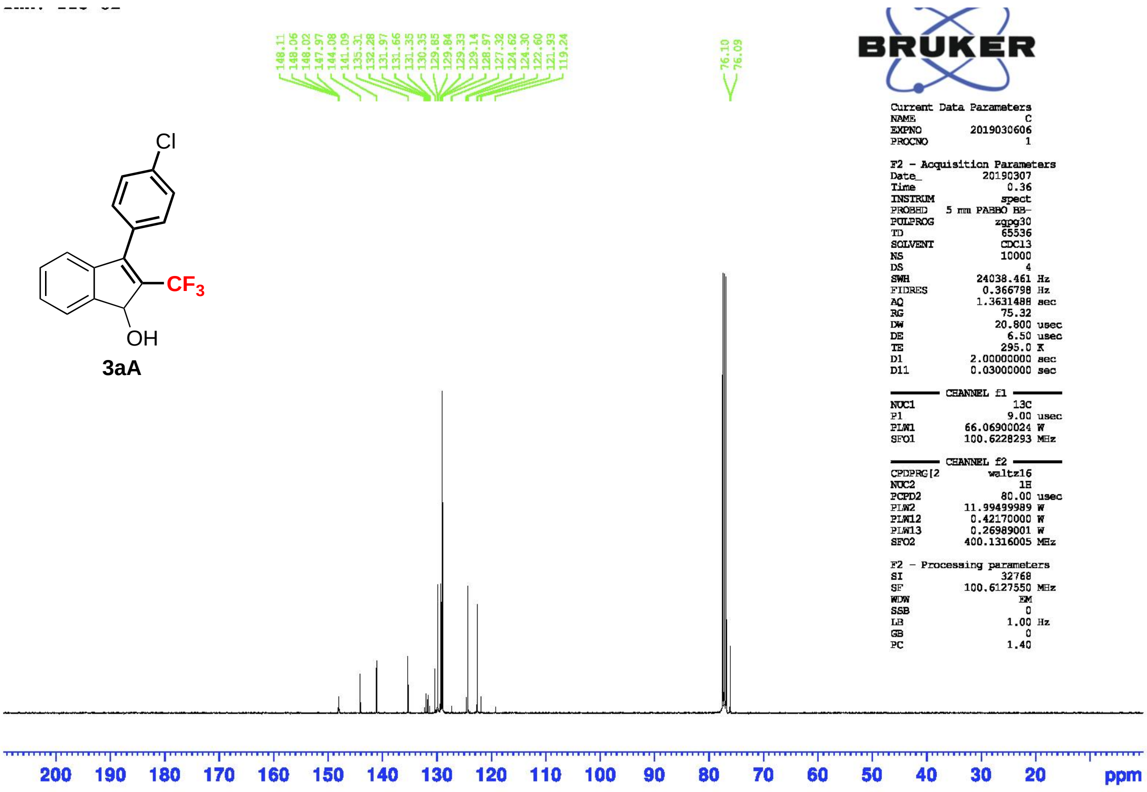

^{1}H NMR spectrum of **3-[4-(*t*-Butyl)phenyl]-2-trifluoromethyl-1*H*-inden-1-ol (3bA)**

rxn. 132 isolate

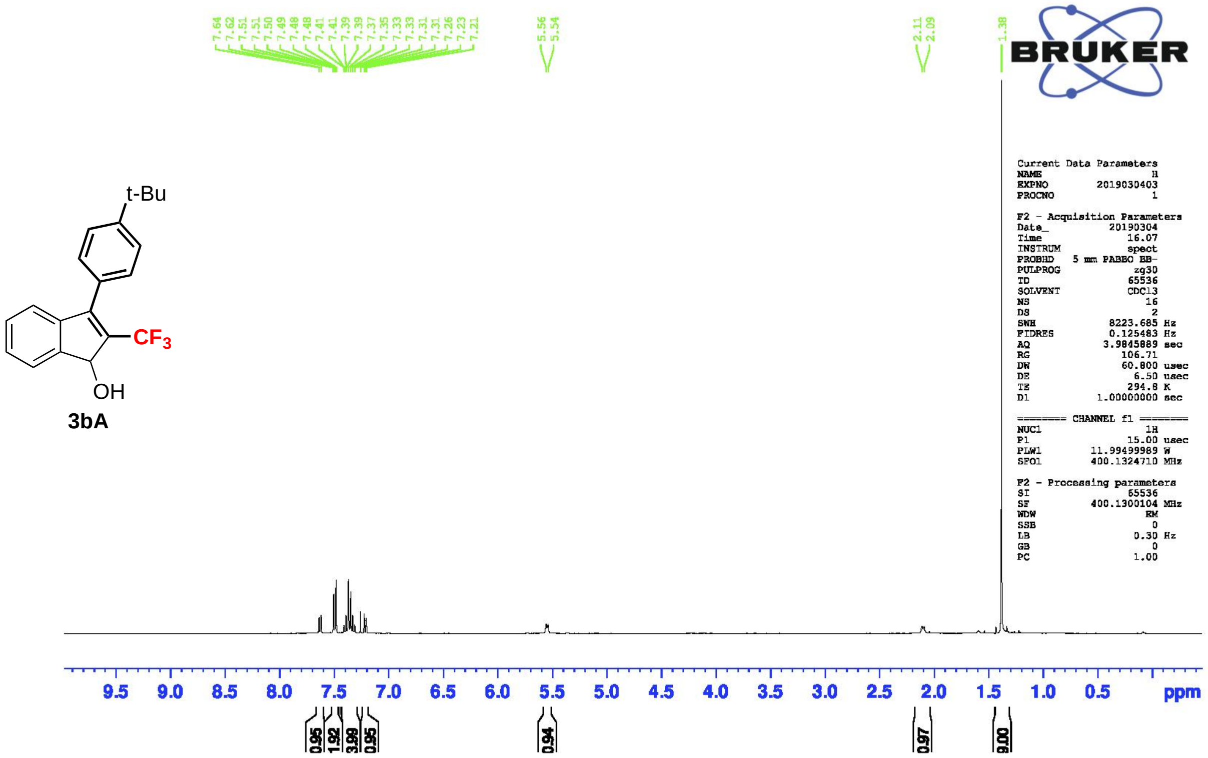

3bA

^{13}C NMR spectrum of **3-[4-(*t*-Butyl)phenyl]-2-trifluoromethyl-1*H*-inden-1-ol (3bA)**

rxn. 132 t-Bu

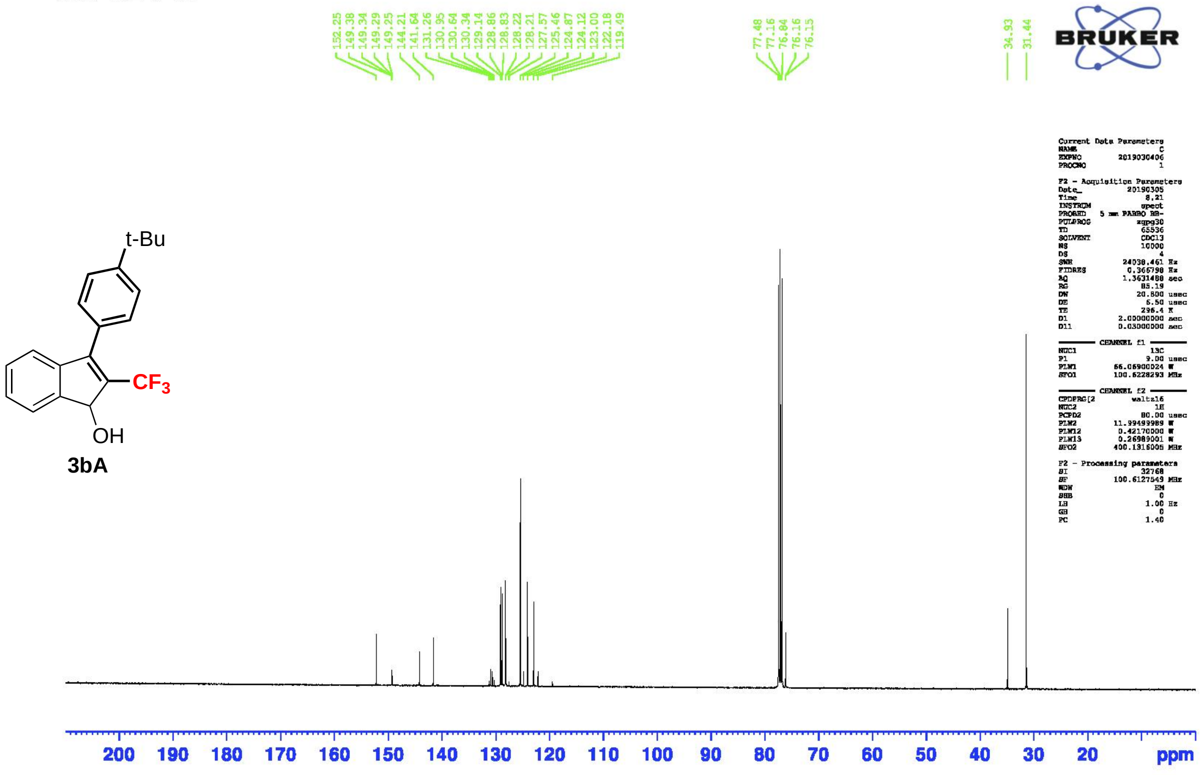

3bA

^{19}F NMR spectrum of **3-[4-(*t*-Butyl)phenyl]-2-trifluoromethyl-1*H*-inden-1-ol (3bA)**

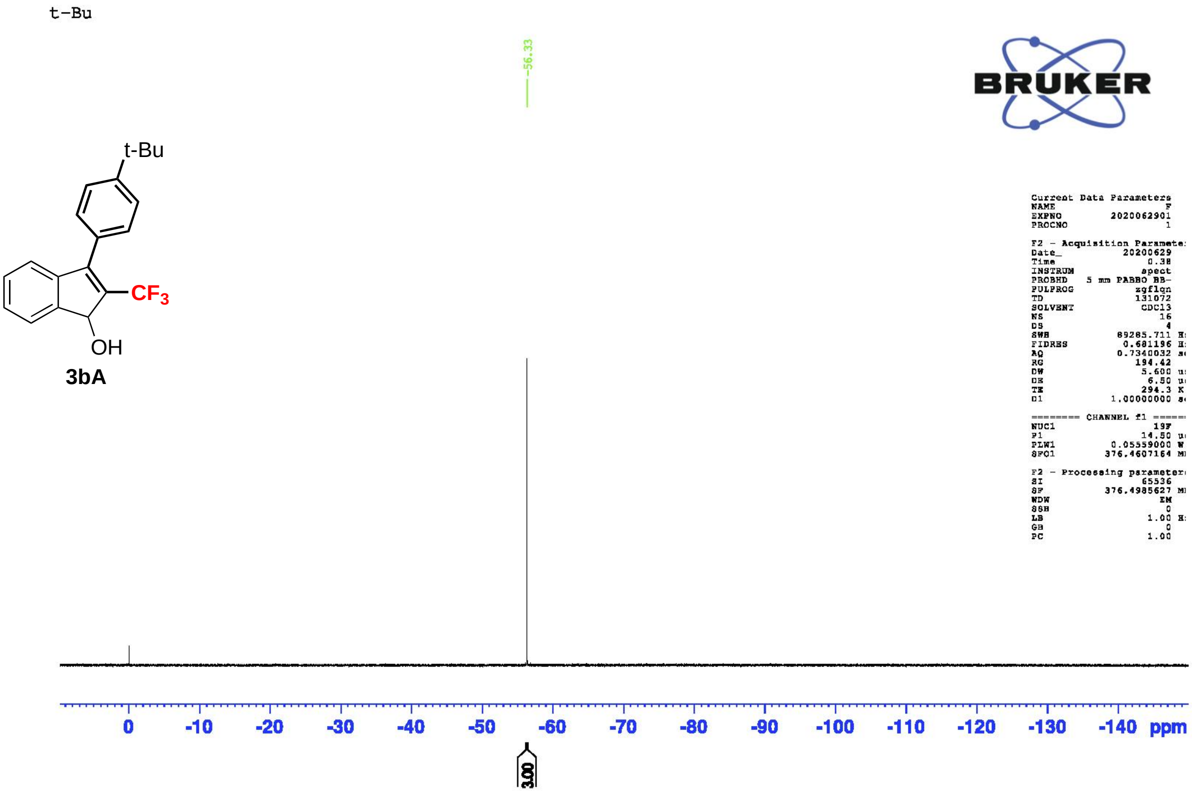

^{1}H NMR spectrum of **3-(4-Methoxyphenyl)-2-trifluoromethyl-1*H*-inden-1-ol (3cA)**

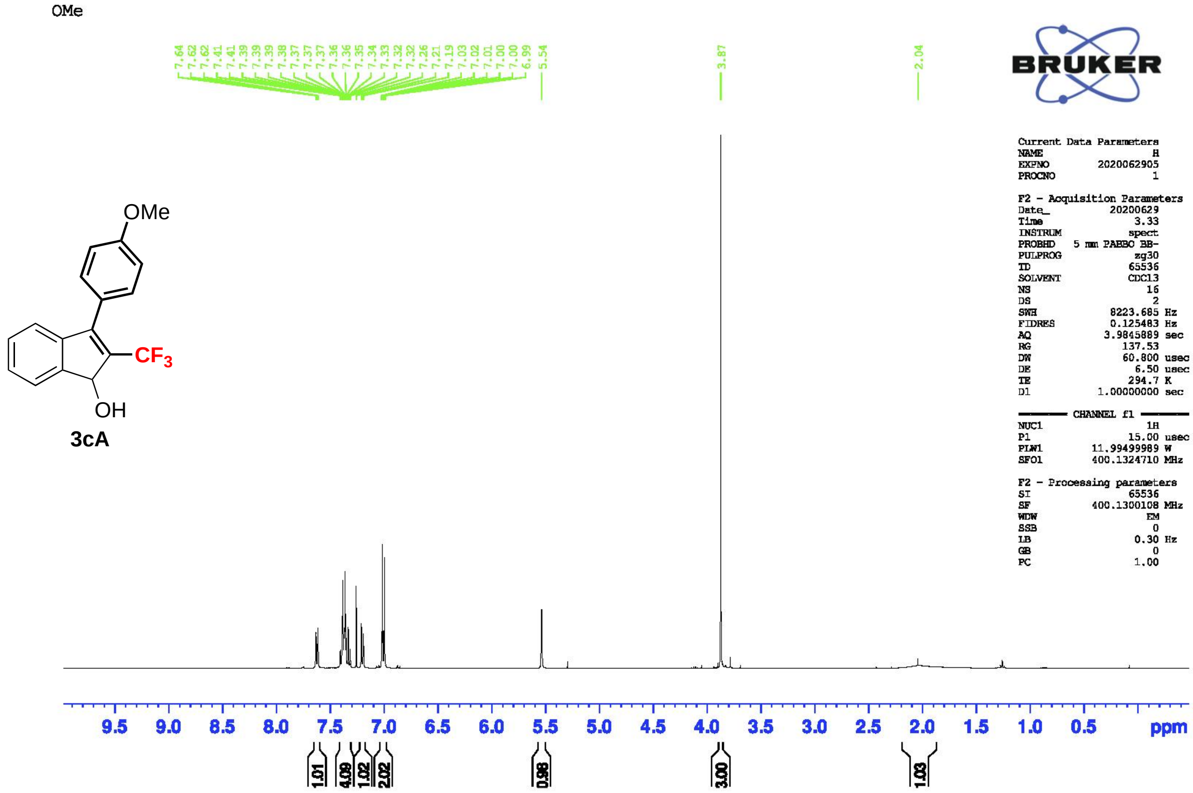

^{13}C NMR spectrum of **3-(4-Methoxyphenyl)-2-trifluoromethyl-1*H*-inden-1-ol (3cA)**

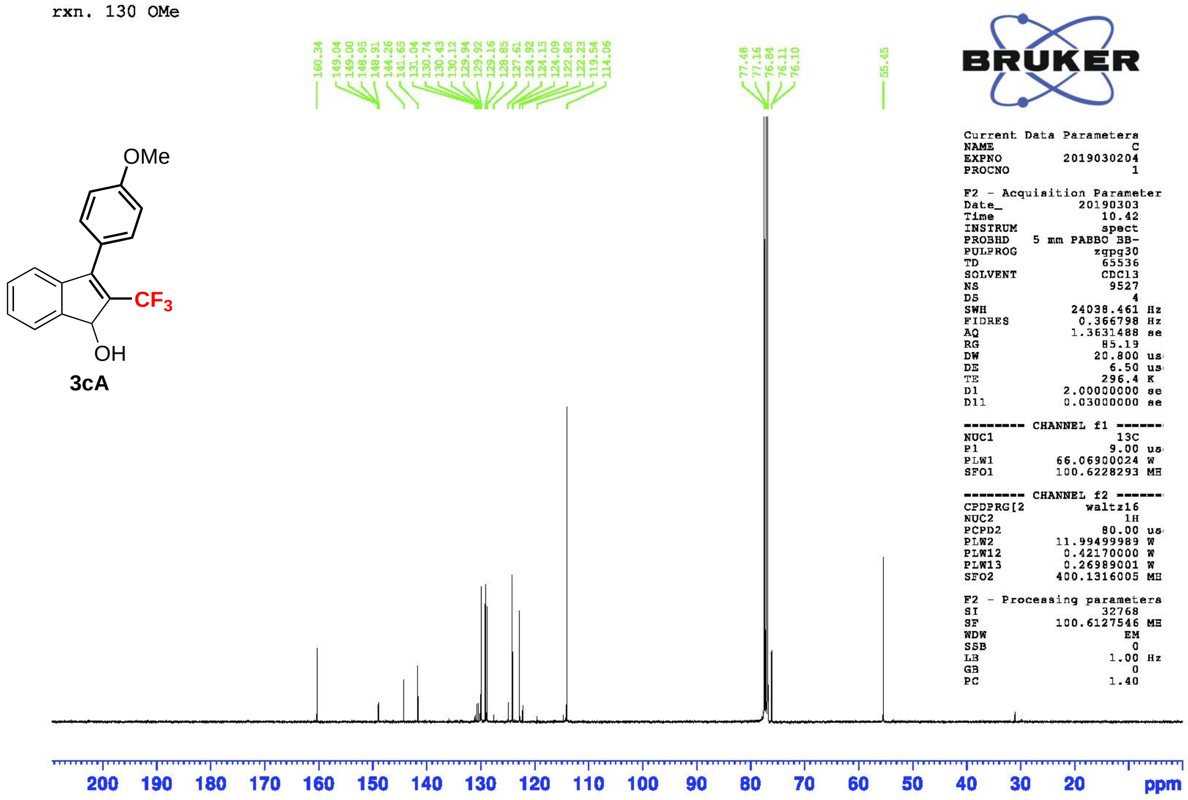

^{19}F NMR spectrum of **3-(4-Methoxyphenyl)-2-trifluoromethyl-1*H*-inden-1-ol (3cA)**

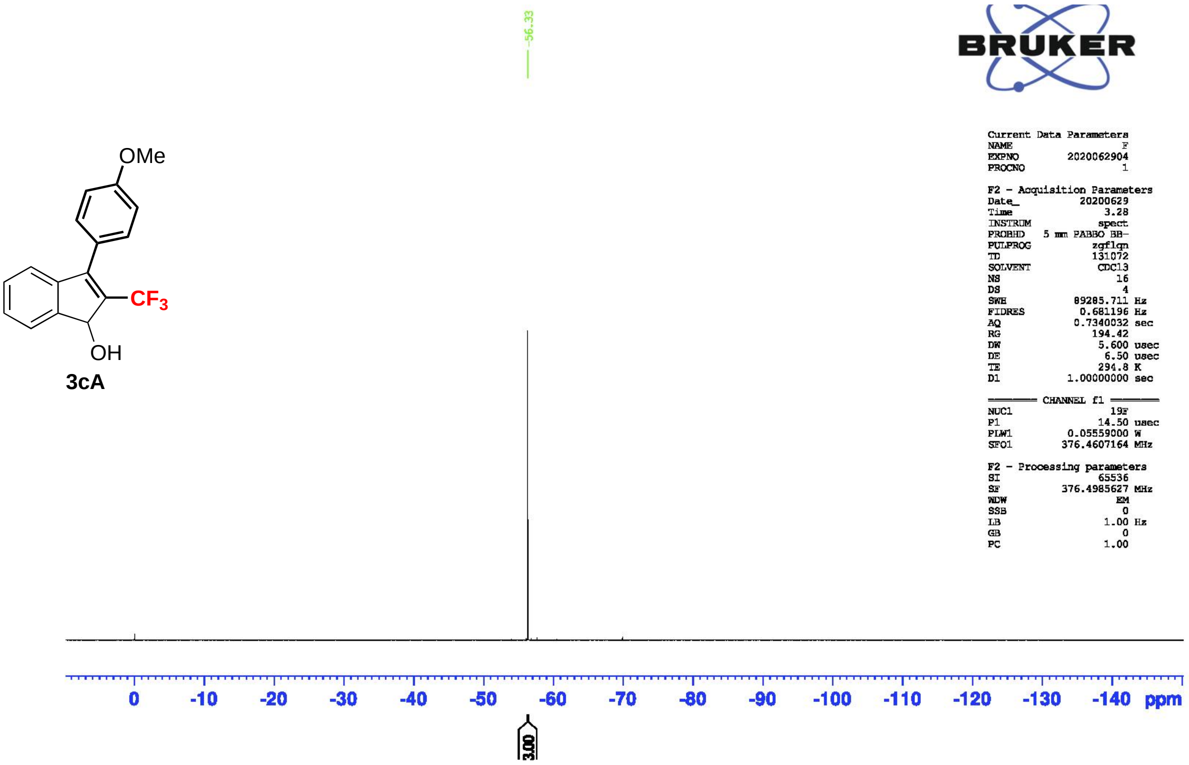

^{1}H NMR spectrum of **3-(4-Biphenyl)-2-trifluoromethyl-1*H*-inden-1-ol (3eA)**

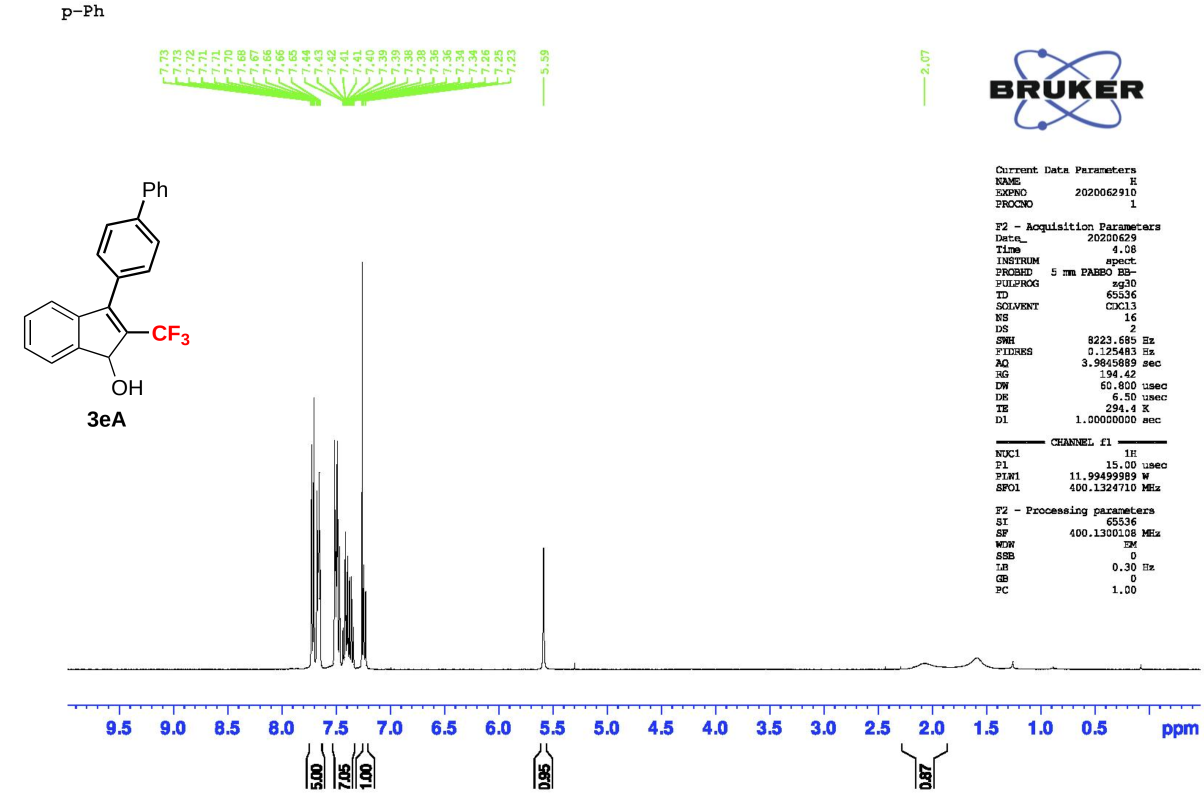

^{13}C NMR spectrum of **3-(4-Biphenyl)-2-trifluoromethyl-1*H*-inden-1-ol (3eA)**

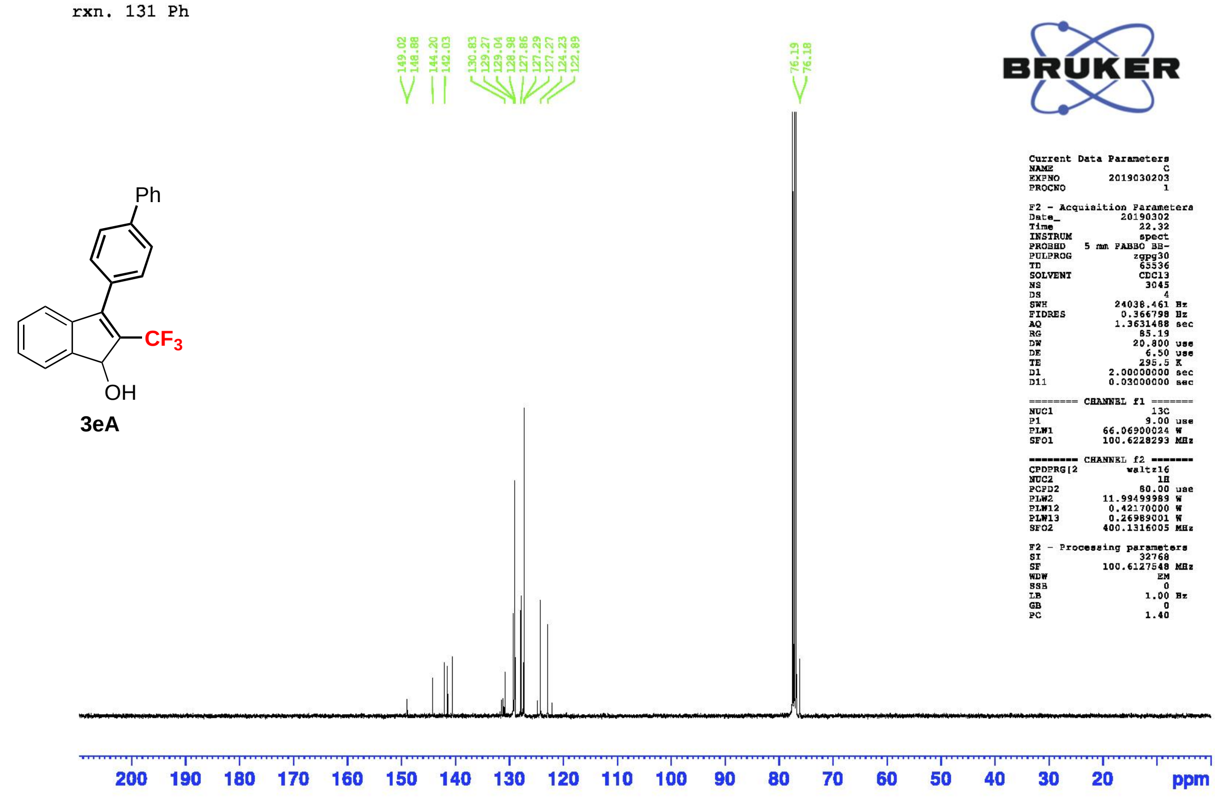

^{19}F NMR spectrum of **3-(4-Biphenyl)-2-trifluoromethyl-1*H*-inden-1-ol (3eA)**

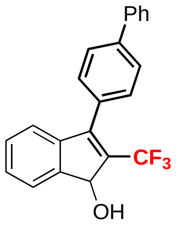

3eA

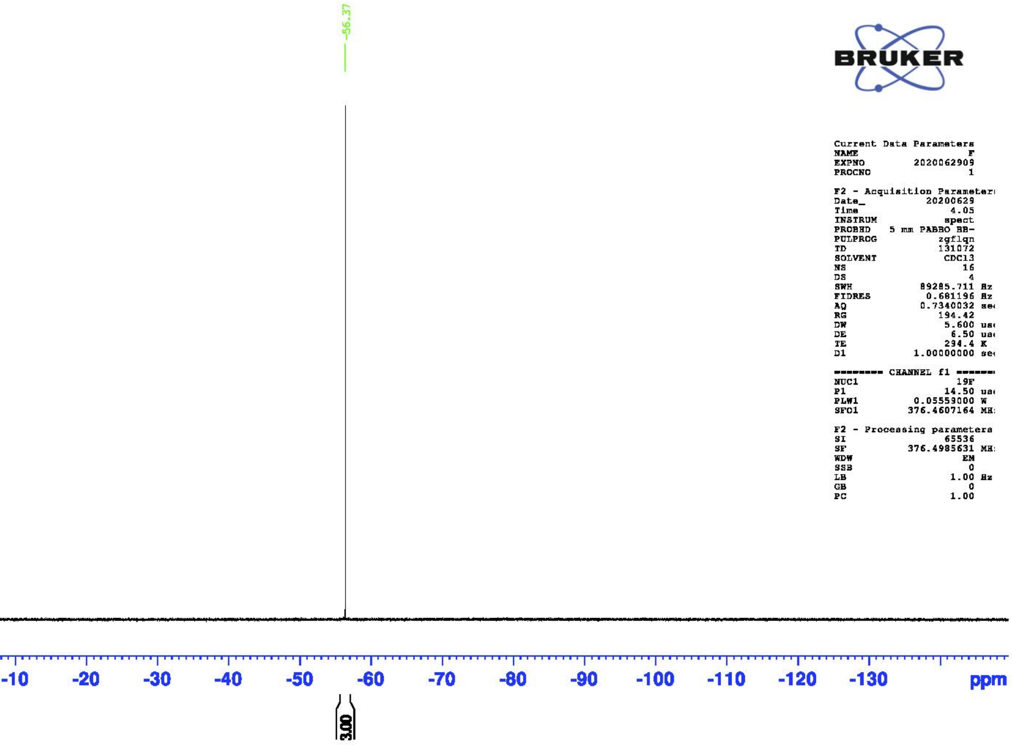

^{1}H NMR spectrum of **3-(1-Naphthyl)-2-trifluoromethyl-1*H*-inden-1-ol (3fA)**

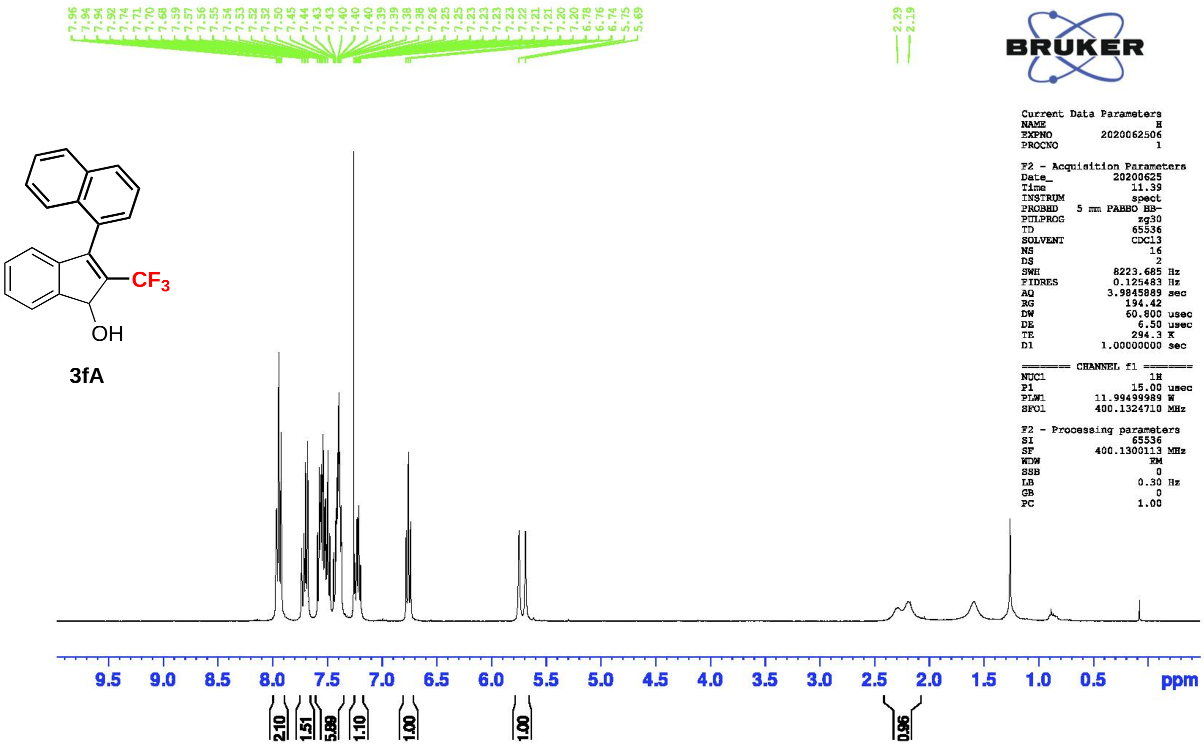

^{13}C NMR spectrum of **3-(1-Naphthyl)-2-trifluoromethyl-1*H*-inden-1-ol (3fA)**

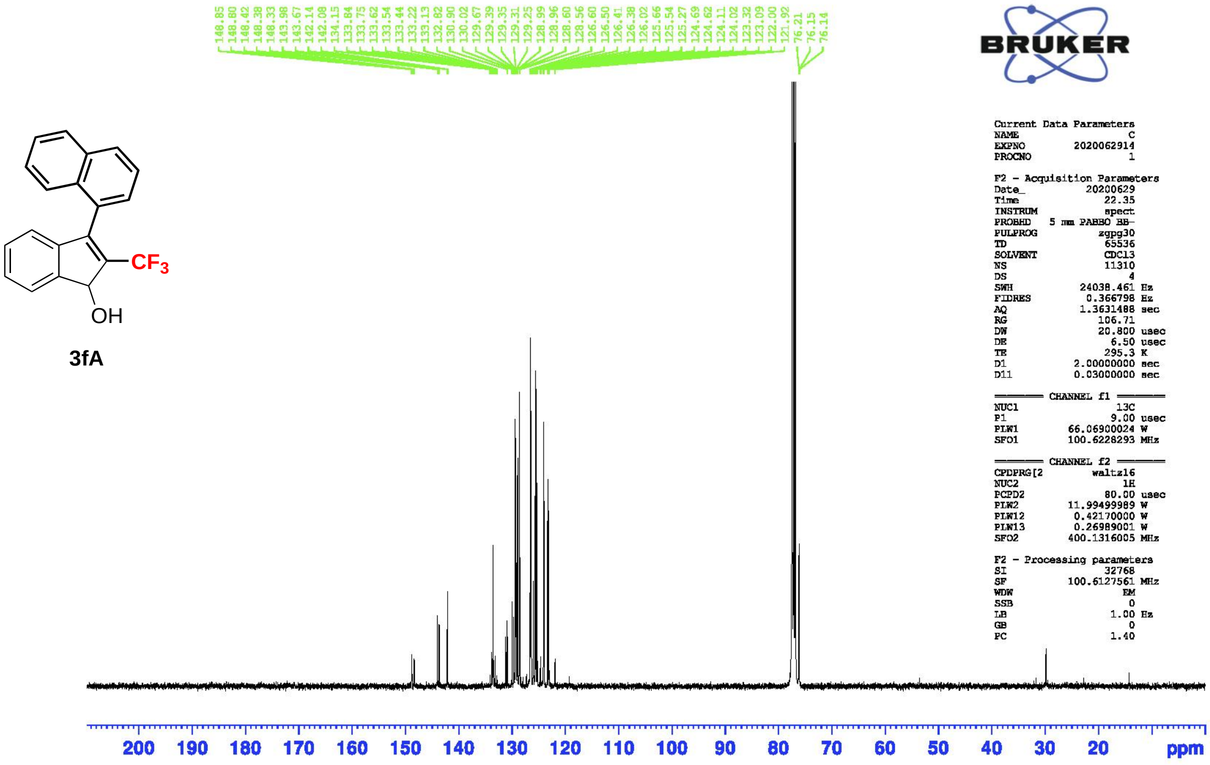

^{19}F NMR spectrum of **3-(1-Naphthyl)-2-trifluoromethyl-1*H*-inden-1-ol (3fA)**

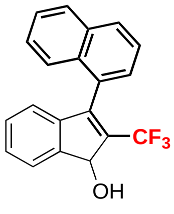

3fA

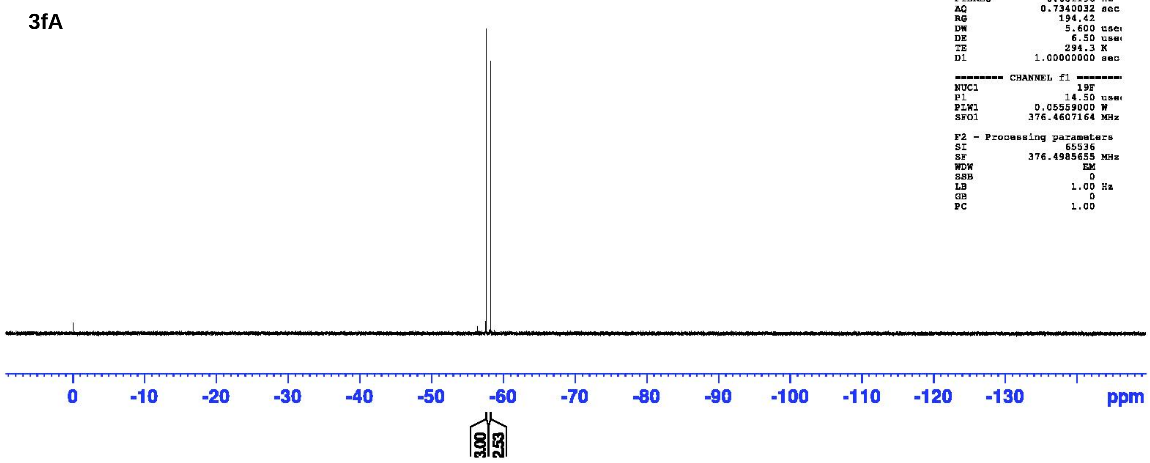

^{1}H NMR spectrum of **3-(3-Chlorophenyl)-2-trifluoromethyl-1*H*-inden-1-ol (3gA)**

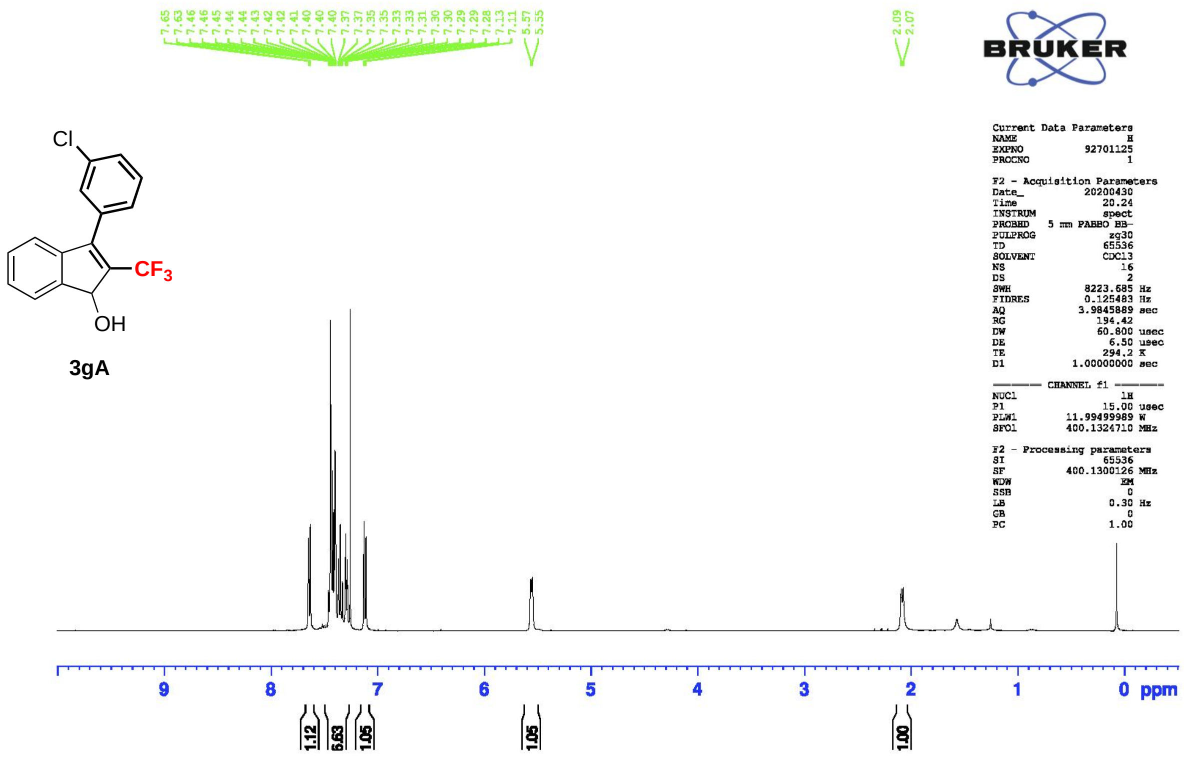

^{13}C NMR spectrum of **3-(3-Chlorophenyl)-2-trifluoromethyl-1*H*-inden-1-ol (3gA)**

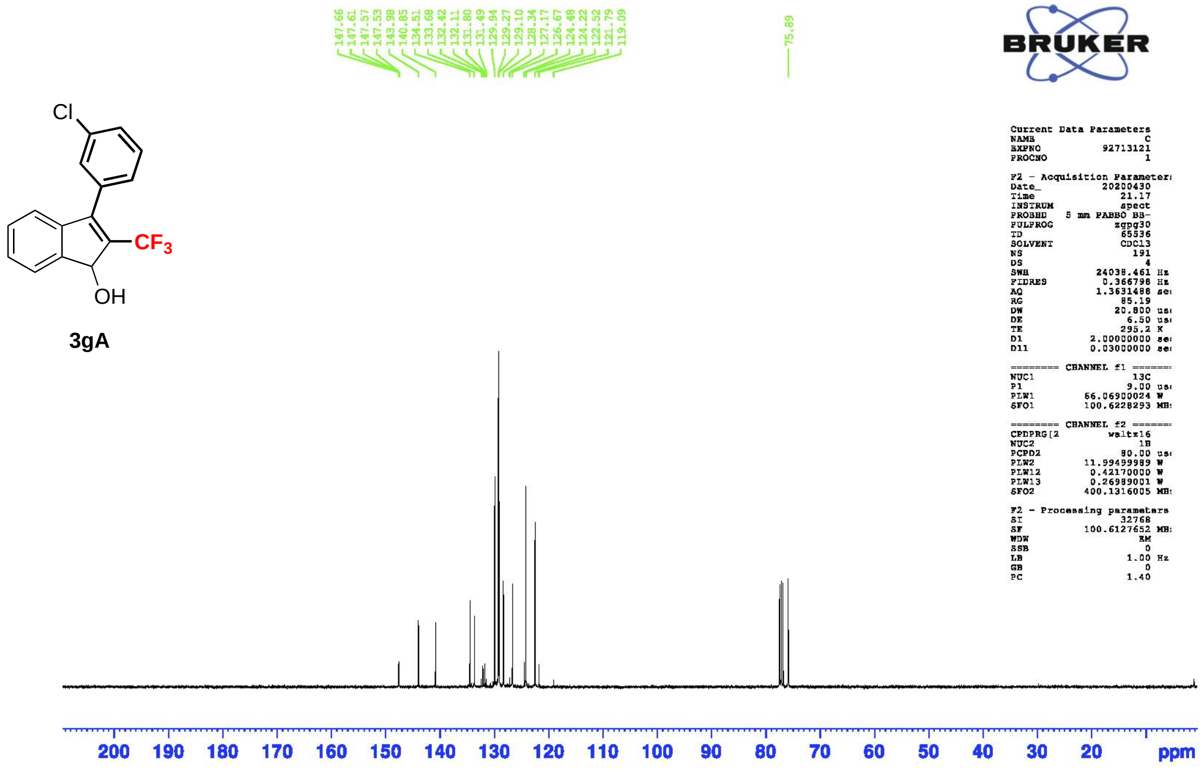

^{19}F NMR spectrum of **3-(3-Chlorophenyl)-2-trifluoromethyl-1*H*-inden-1-ol (3gA)**

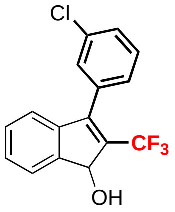

3gA

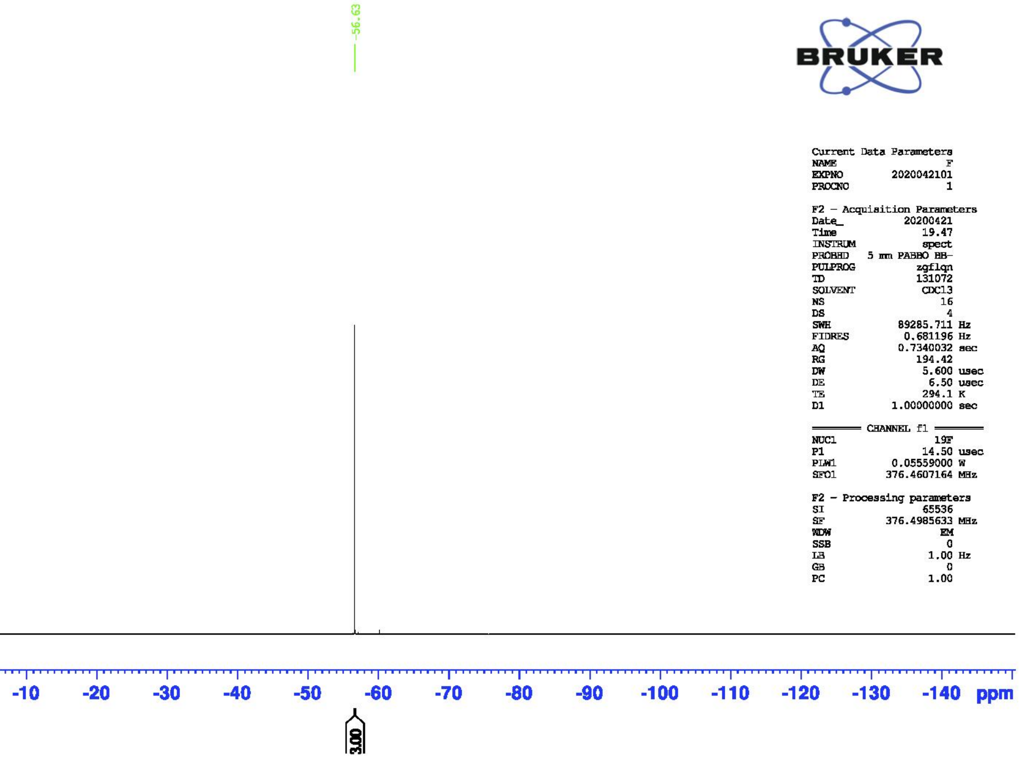

^{1}H NMR spectrum of **3-(4-Chlorophenyl)-2-difluoromethyl-1*H*-inden-1-ol (3hA)**

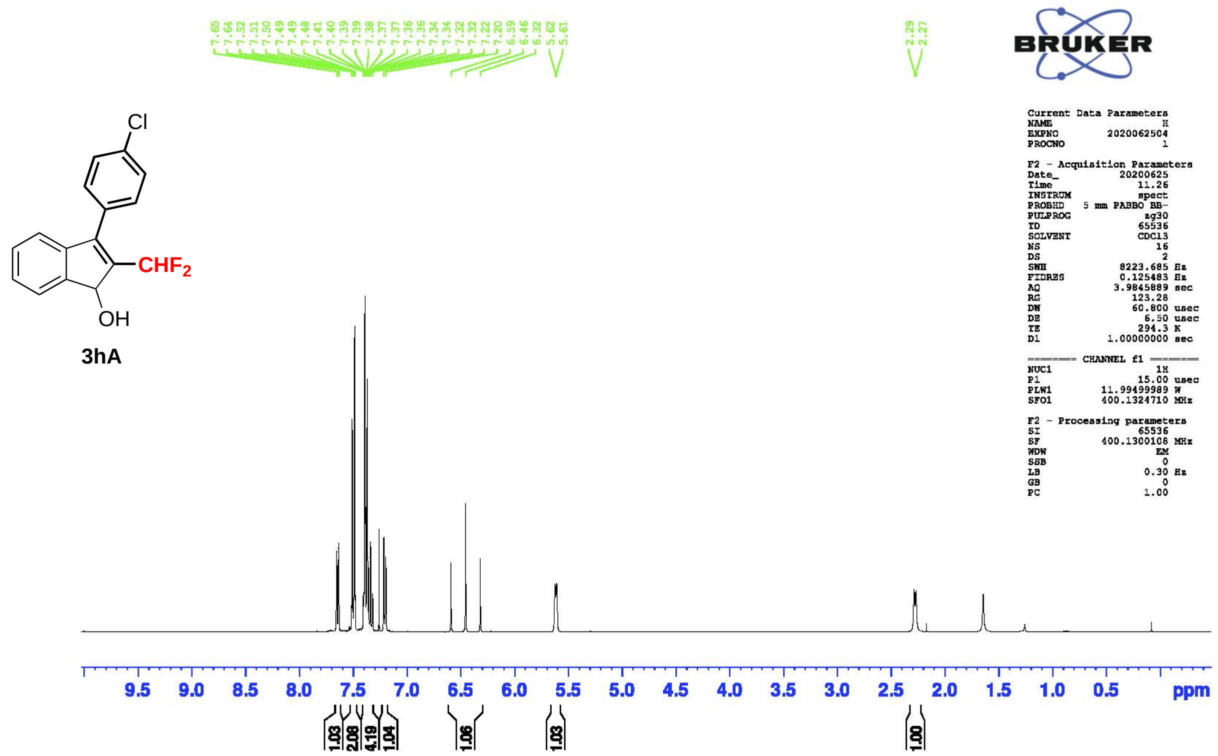

^{13}C NMR spectrum of **3-(4-Chlorophenyl)-2-difluoromethyl-1*H*-inden-1-ol (3hA)**

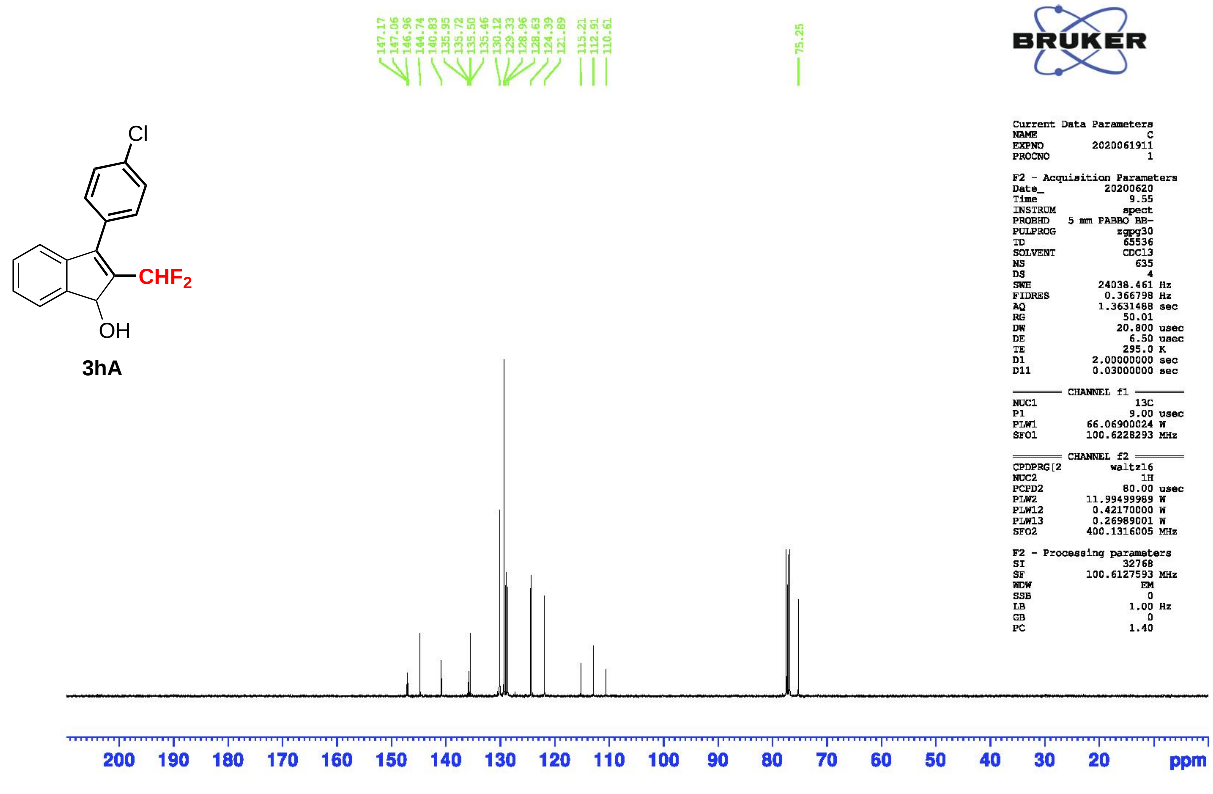

^{19}F NMR spectrum of **3-(4-Chlorophenyl)-2-difluoromethyl-1*H*-inden-1-ol (3hA)**

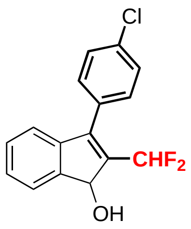

3hA

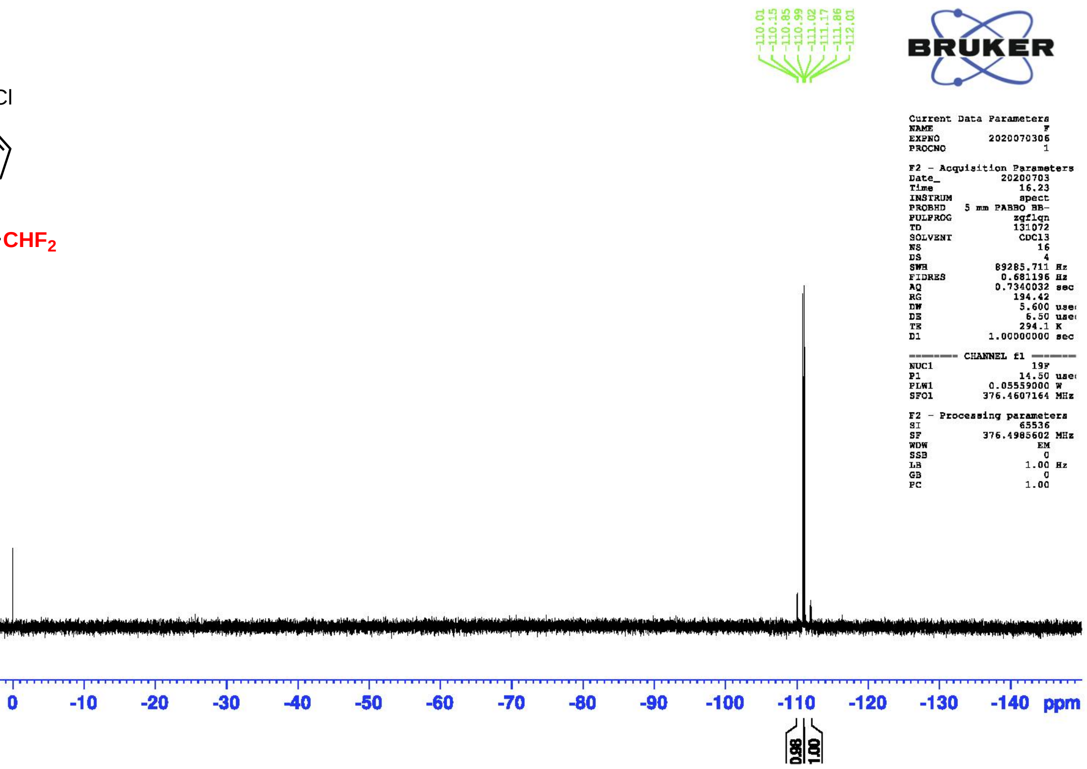

^{1}H NMR spectrum of **3-(4-Chlorophenyl)-2-nonafluorobutyl-1*H*-inden-1-ol (3iA)**

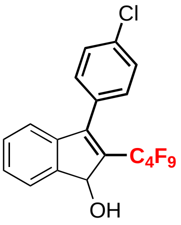

3iA

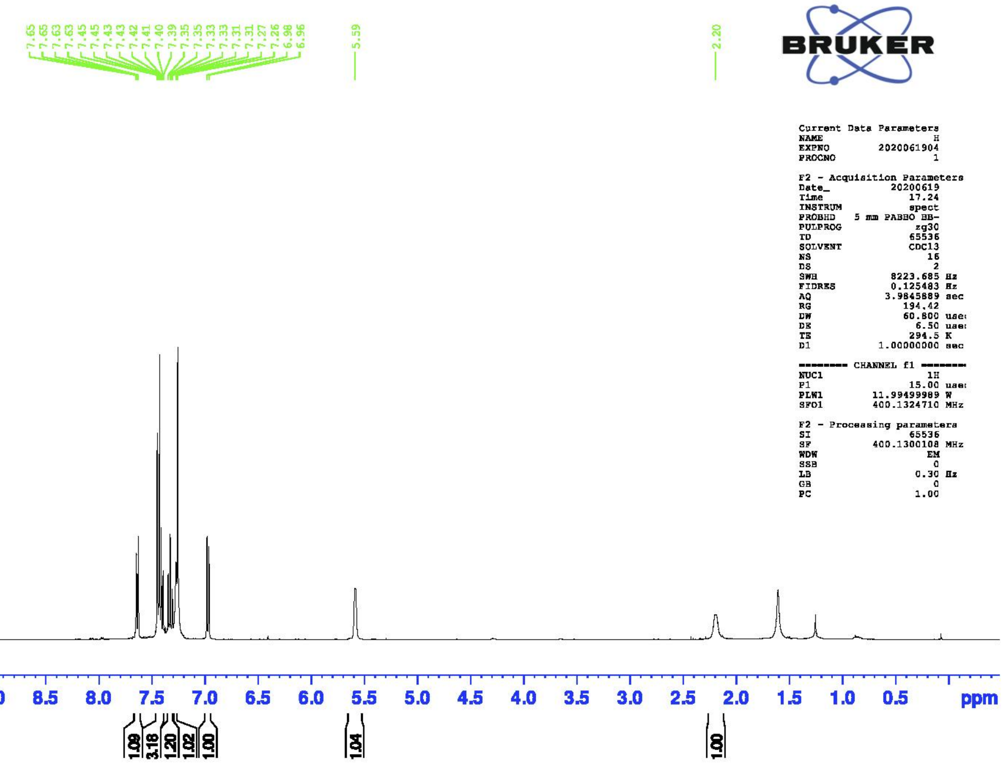

^{13}C NMR spectrum of **3-(4-Chlorophenyl)-2-nonafluorobutyl-1*H*-inden-1-ol (3iA)**

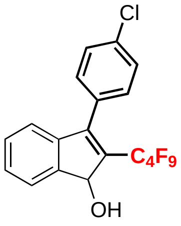

3iA

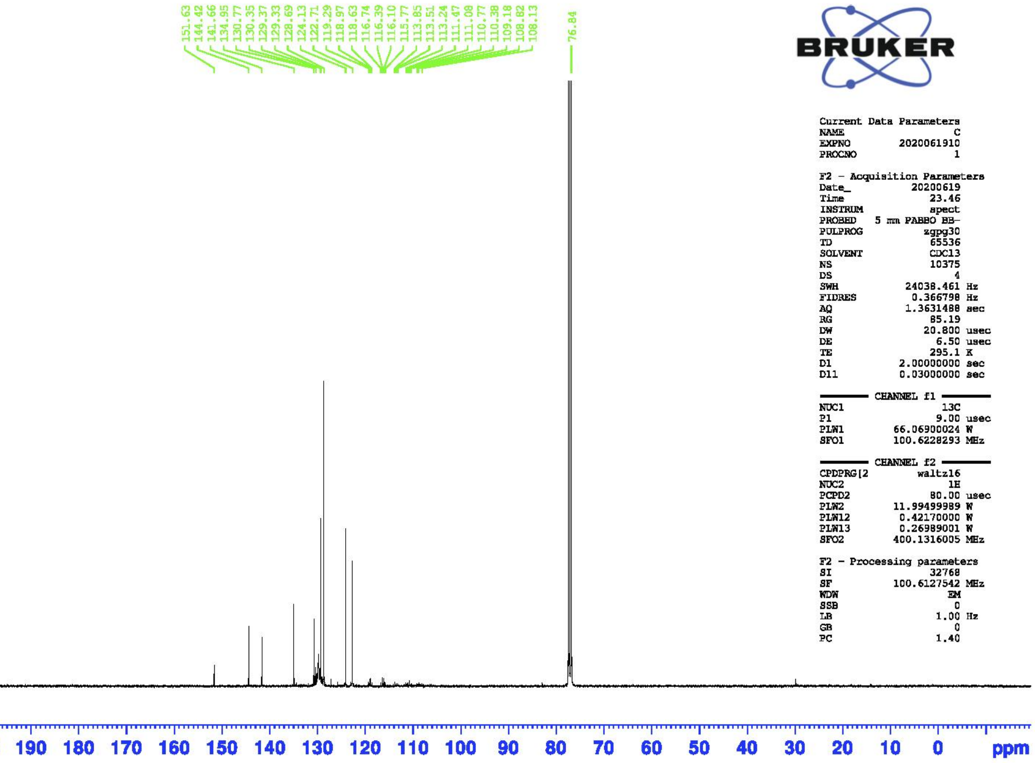

^{19}F NMR spectrum of **3-(4-Chlorophenyl)-2-nonafluorobutyl-1*H*-inden-1-ol (3iA)**

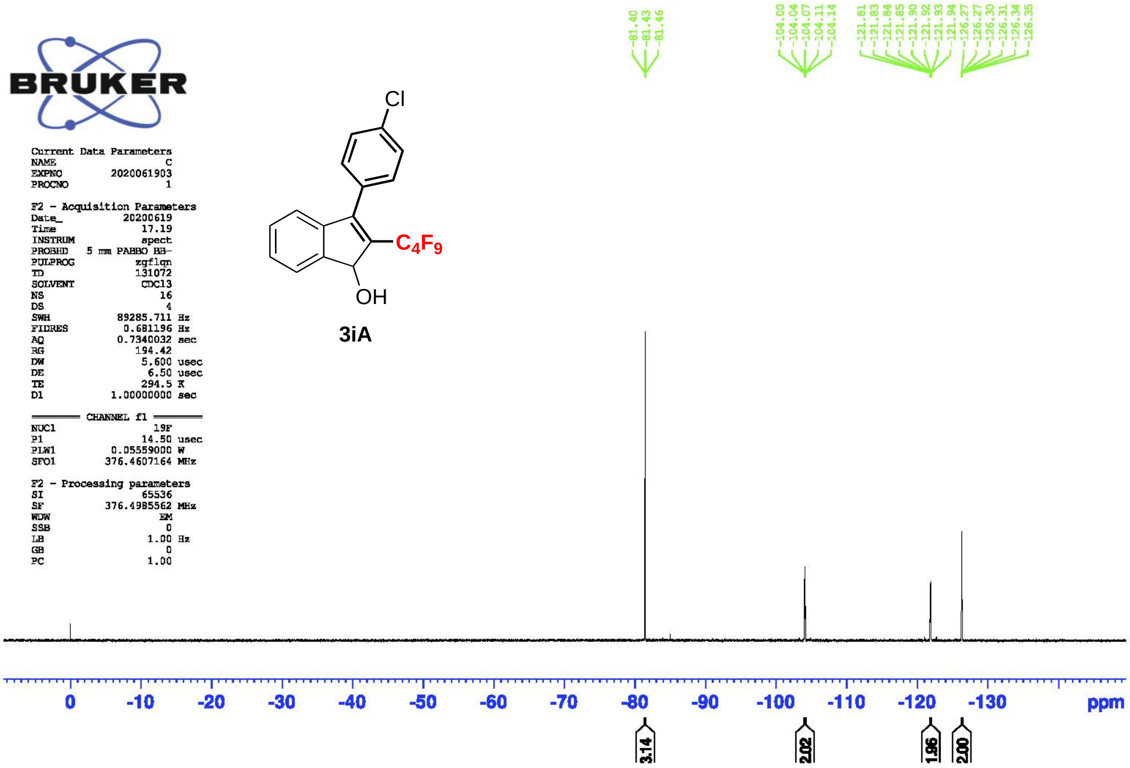

^{1}H NMR spectrum of **3-(4-Chlorophenyl)-6-fluoro-2-trifluoromethyl-1*H*-inden-1-ol (3aB)**

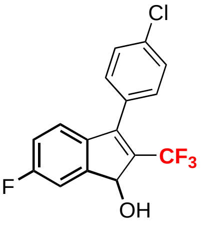

3aB

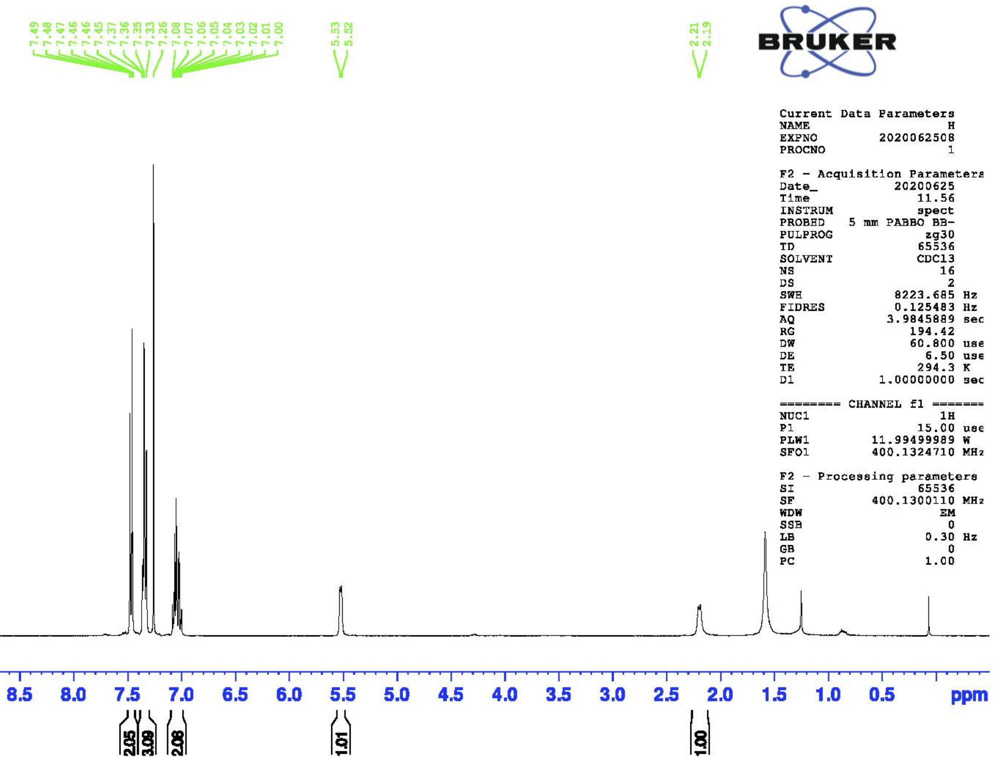

^{13}C NMR spectrum of **3-(4-Chlorophenyl)-6-fluoro-2-trifluoromethyl-1*H*-inden-1-ol (3aB)**

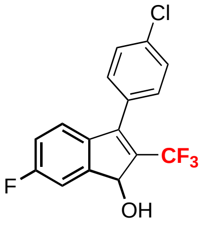

3aB

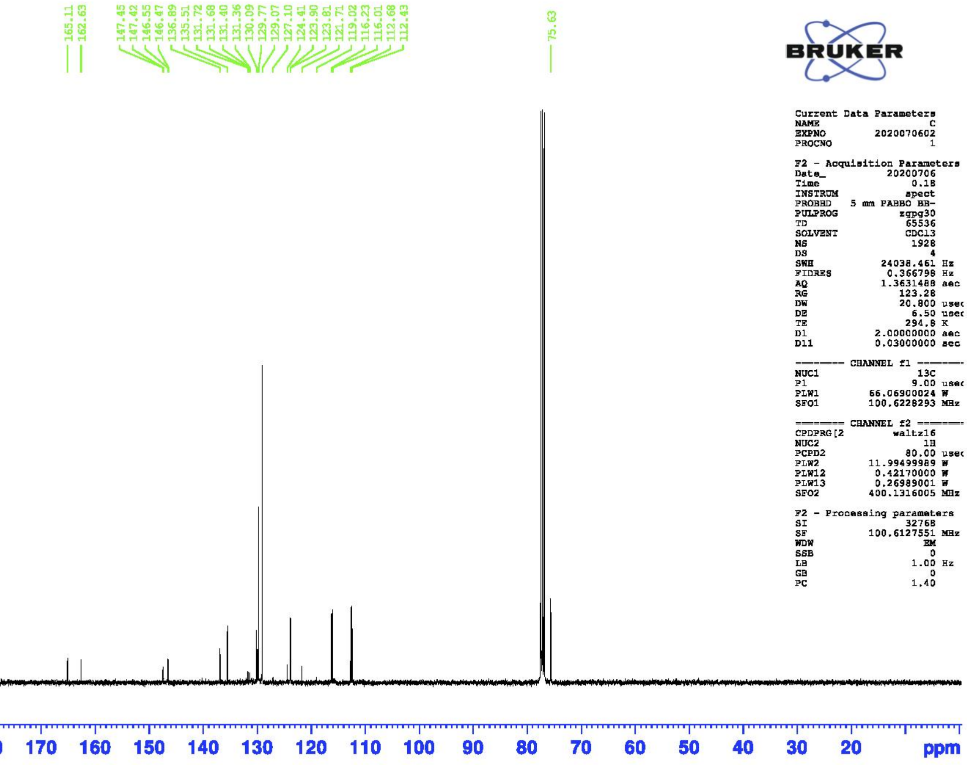

^{19}F NMR spectrum of **3-(4-Chlorophenyl)-6-fluoro-2-trifluoromethyl-1*H*-inden-1-ol (3aB)**

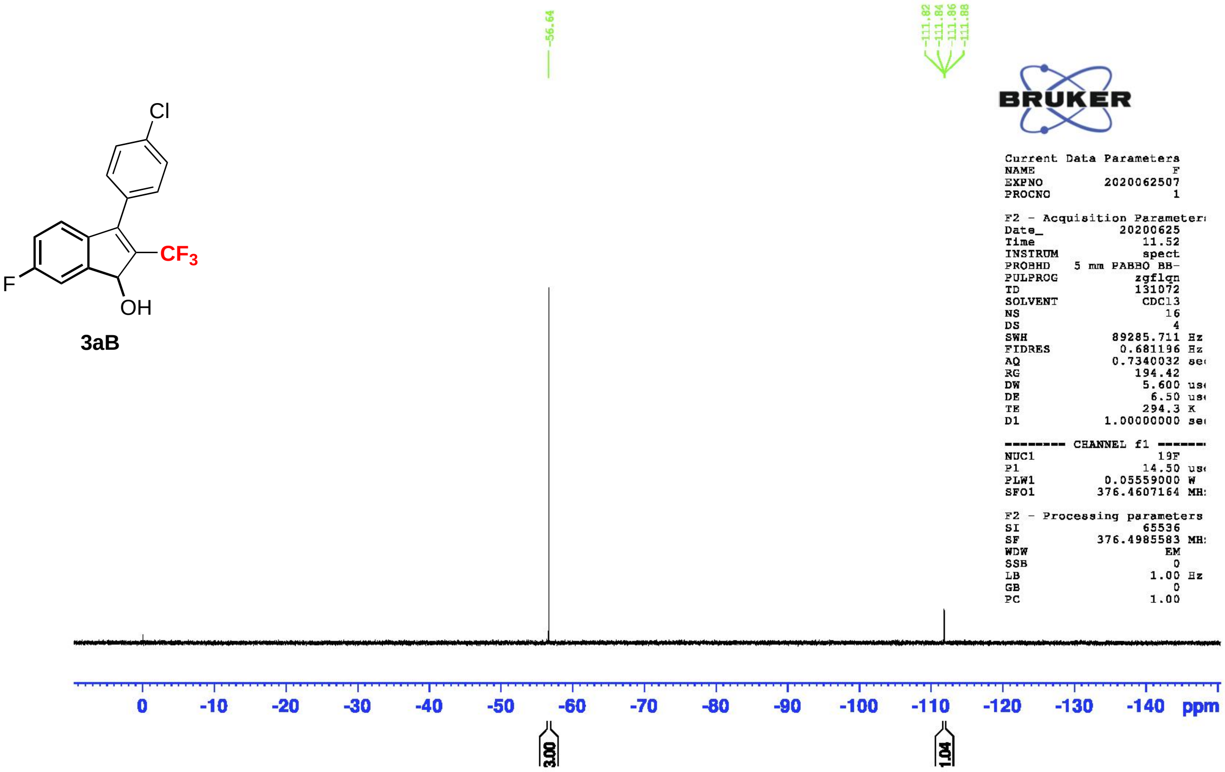

3aB

^{1}H NMR spectrum of **6-Chloro-3-(4-chlorophenyl)-2-trifluoromethyl-1*H*-inden-1-ol (3aC)**

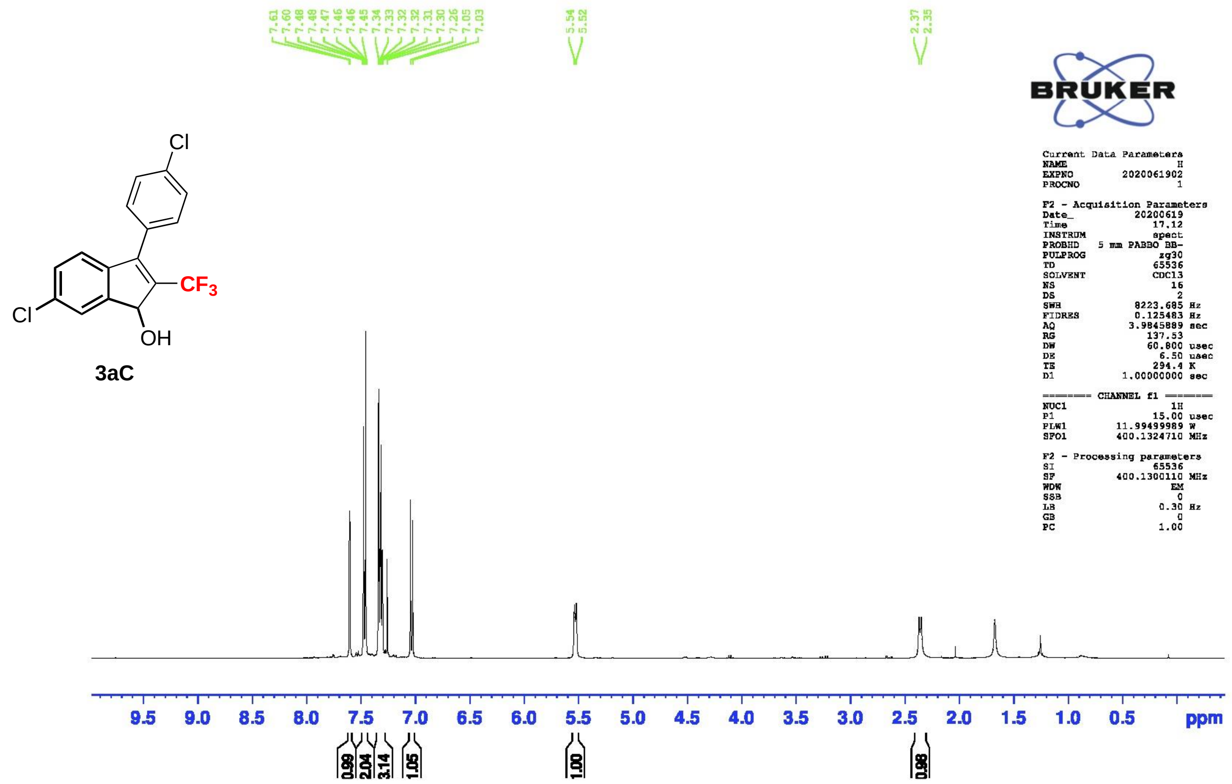

^{13}C NMR spectrum of **6-Chloro-3-(4-chlorophenyl)-2-trifluoromethyl-1*H*-inden-1-ol (3aC)**

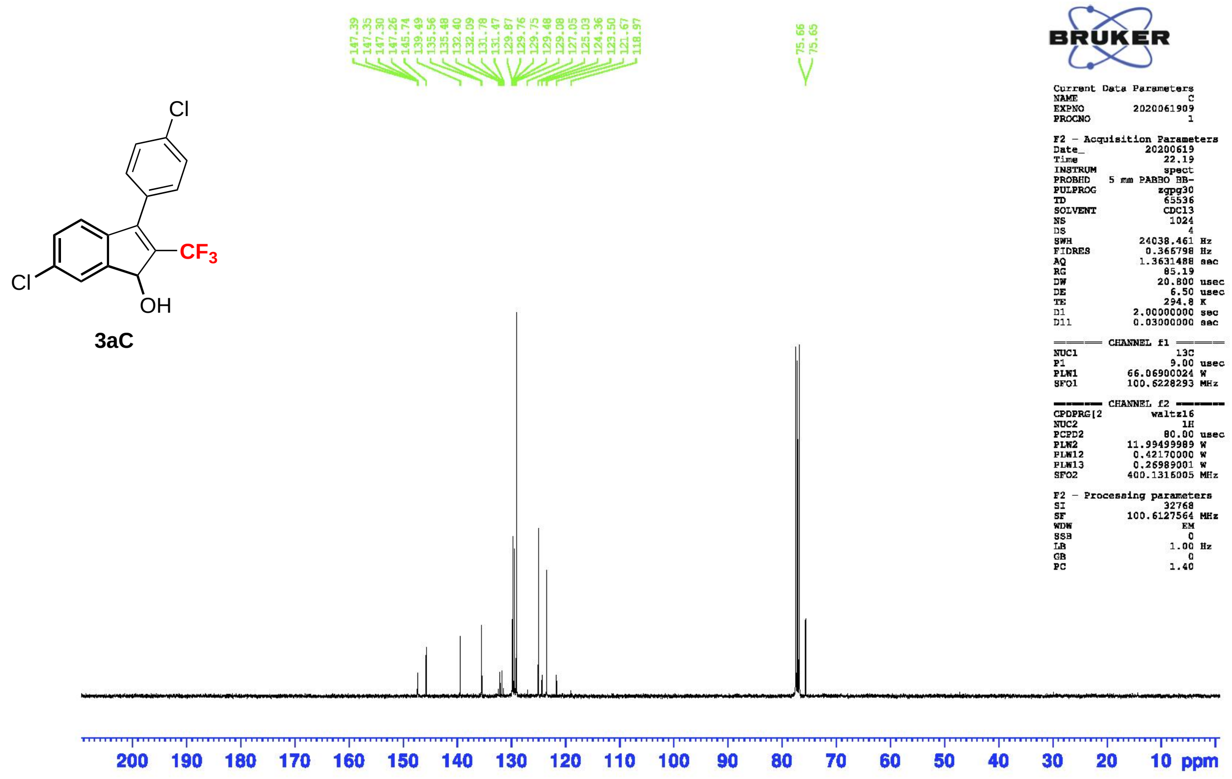

^{19}F NMR spectrum of **6-Chloro-3-(4-chlorophenyl)-2-trifluoromethyl-1*H*-inden-1-ol (3aC)**

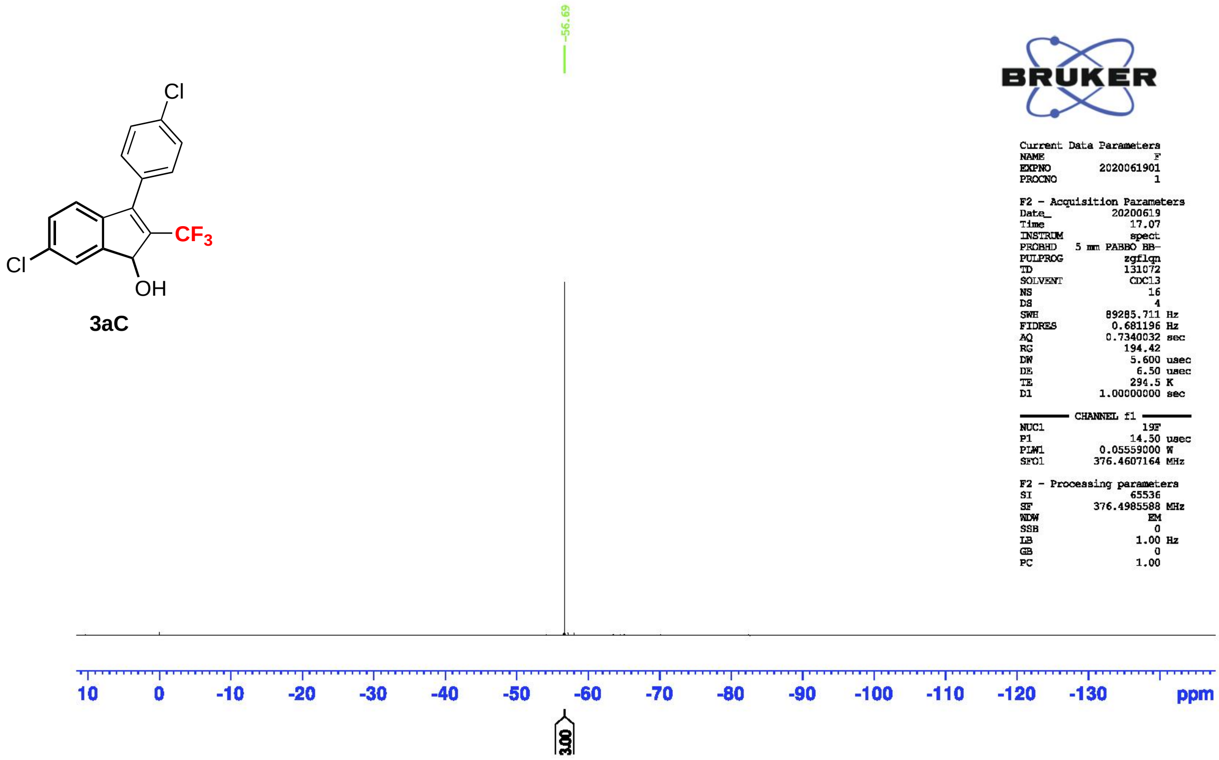

^{1}H NMR spectrum of **3-(4-Chlorophenyl)-6-methoxy-2-trifluoromethyl-1*H*-inden-1-ol (3aD)**

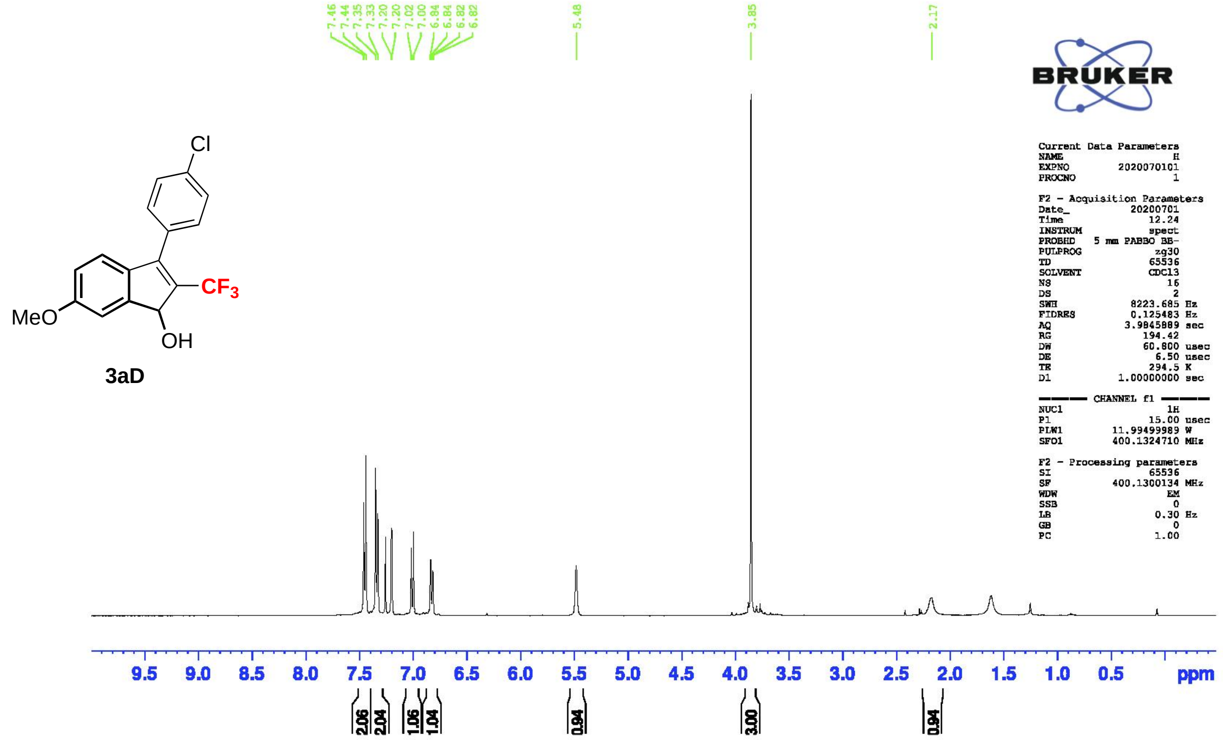

^{13}C NMR spectrum of **3-(4-Chlorophenyl)-6-methoxy-2-trifluoromethyl-1*H*-inden-1-ol (3aD)**

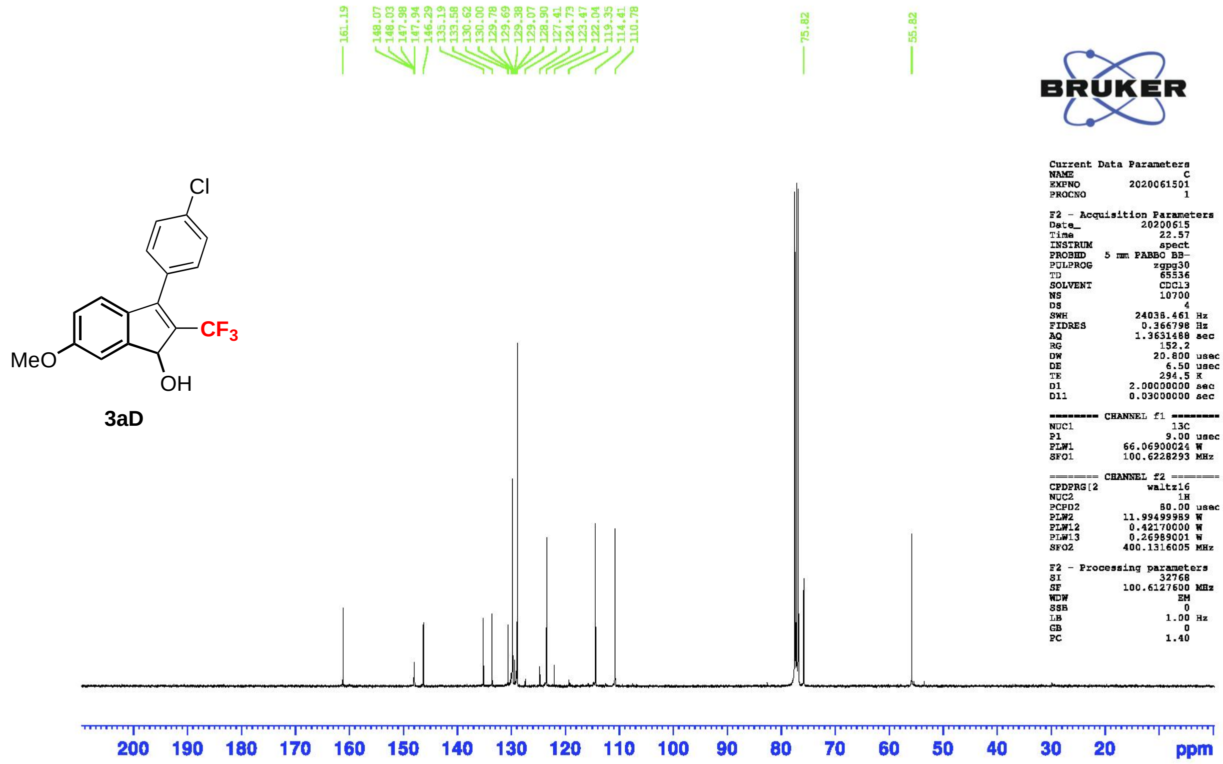

^{19}F NMR spectrum of **3-(4-Chlorophenyl)-6-methoxy-2-trifluoromethyl-1*H*-inden-1-ol (3aD)**

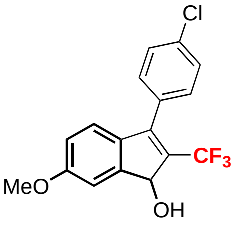

3aD

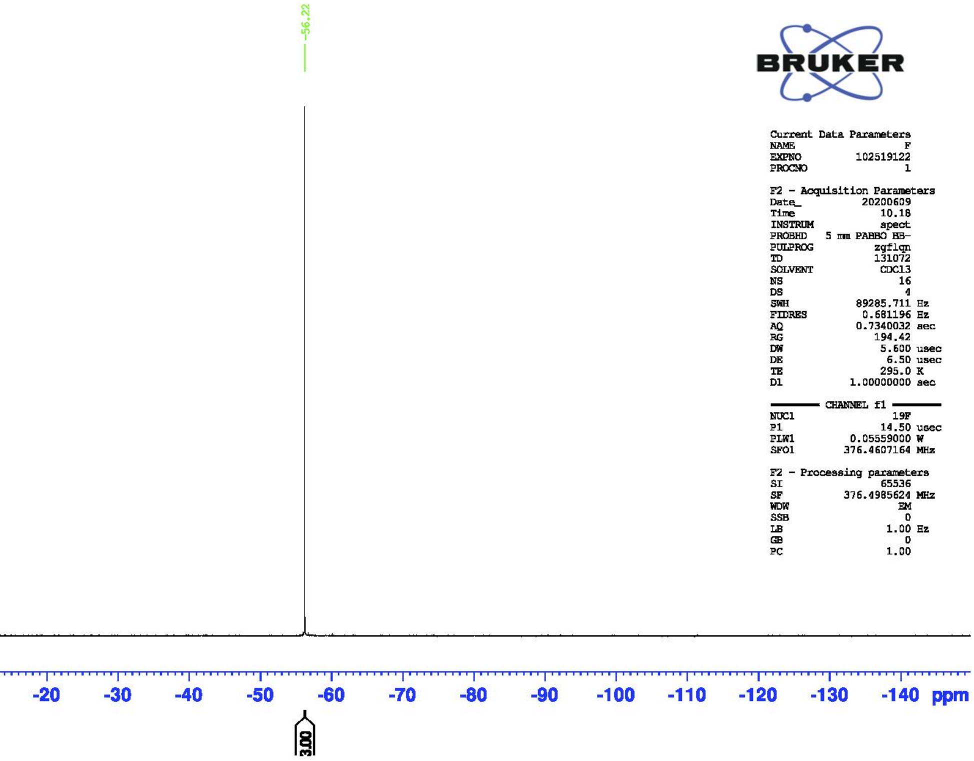

^{1}H NMR spectrum of **6-Benzyloxy-3-(4-chlorophenyl)-2-trifluoromethyl-1*H*-inden-1-ol (3aE)**

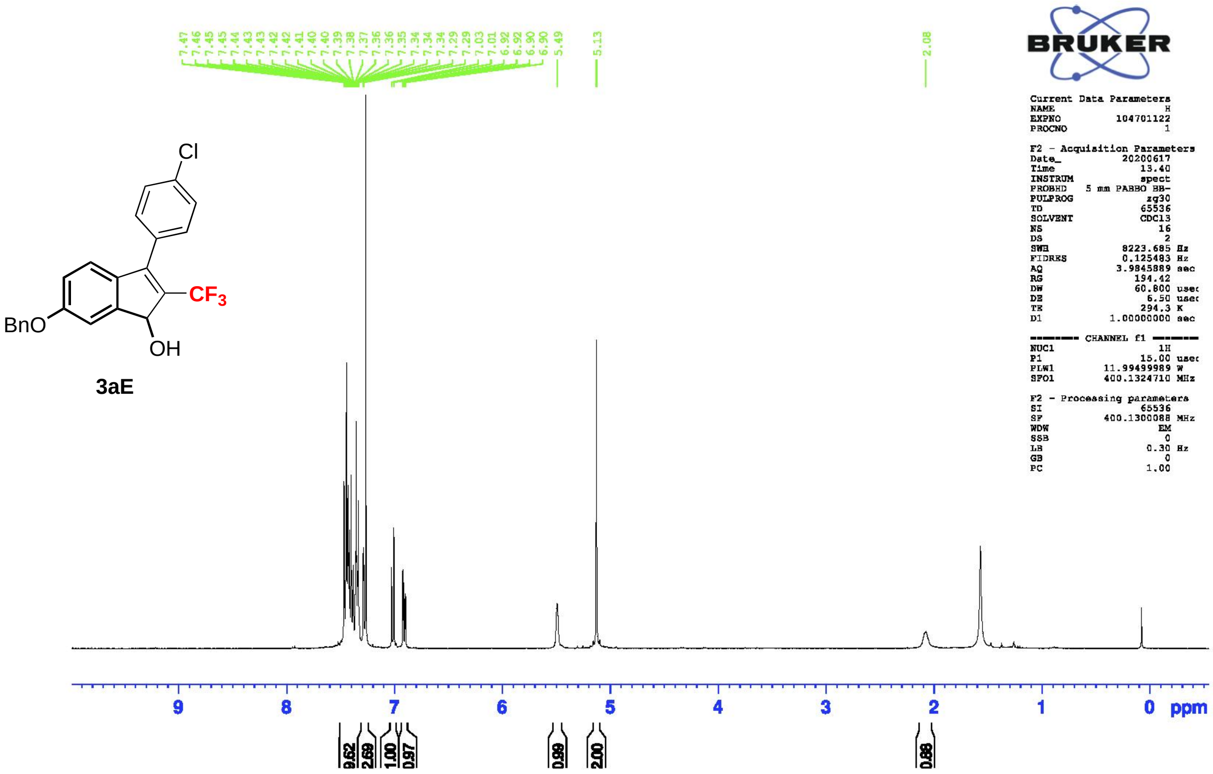

^{13}C NMR spectrum of **6-Benzyloxy-3-(4-chlorophenyl)-2-trifluoromethyl-1*H*-inden-1-ol (3aE)**

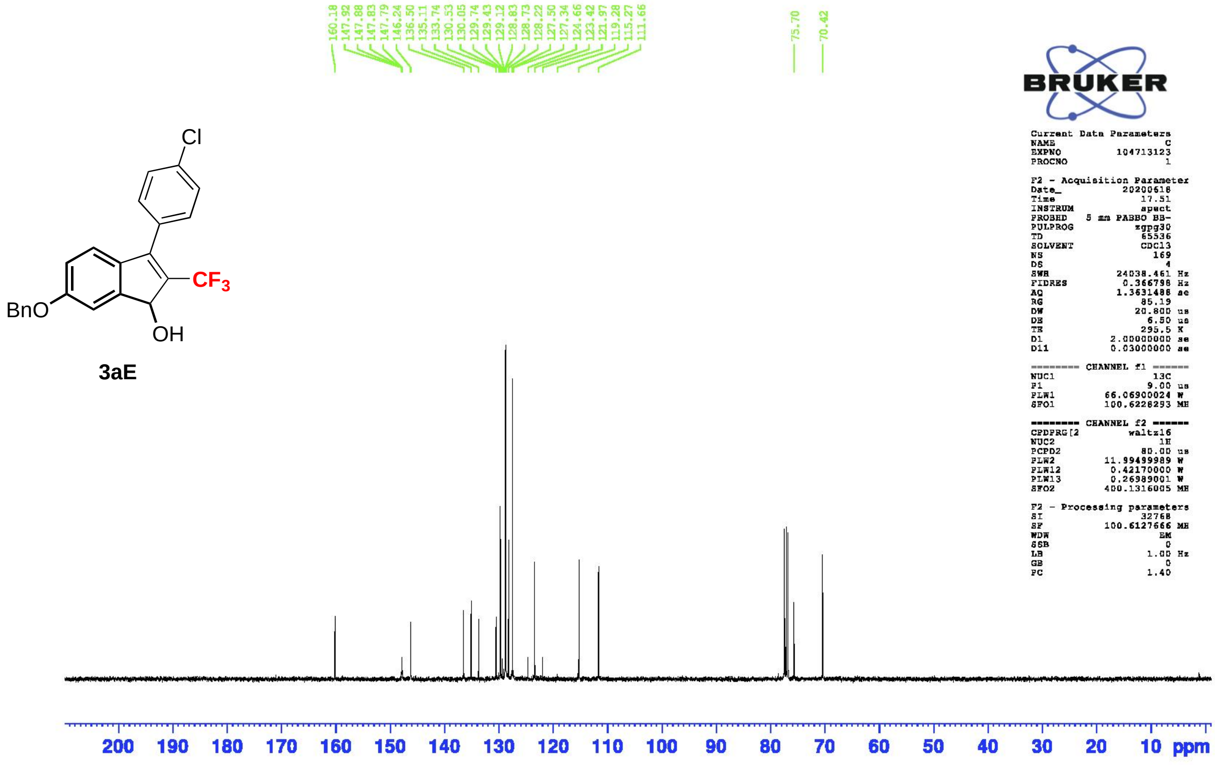

^{19}F NMR spectrum of **6-Benzyloxy-3-(4-chlorophenyl)-2-trifluoromethyl-1*H*-inden-1-ol (3aE)**

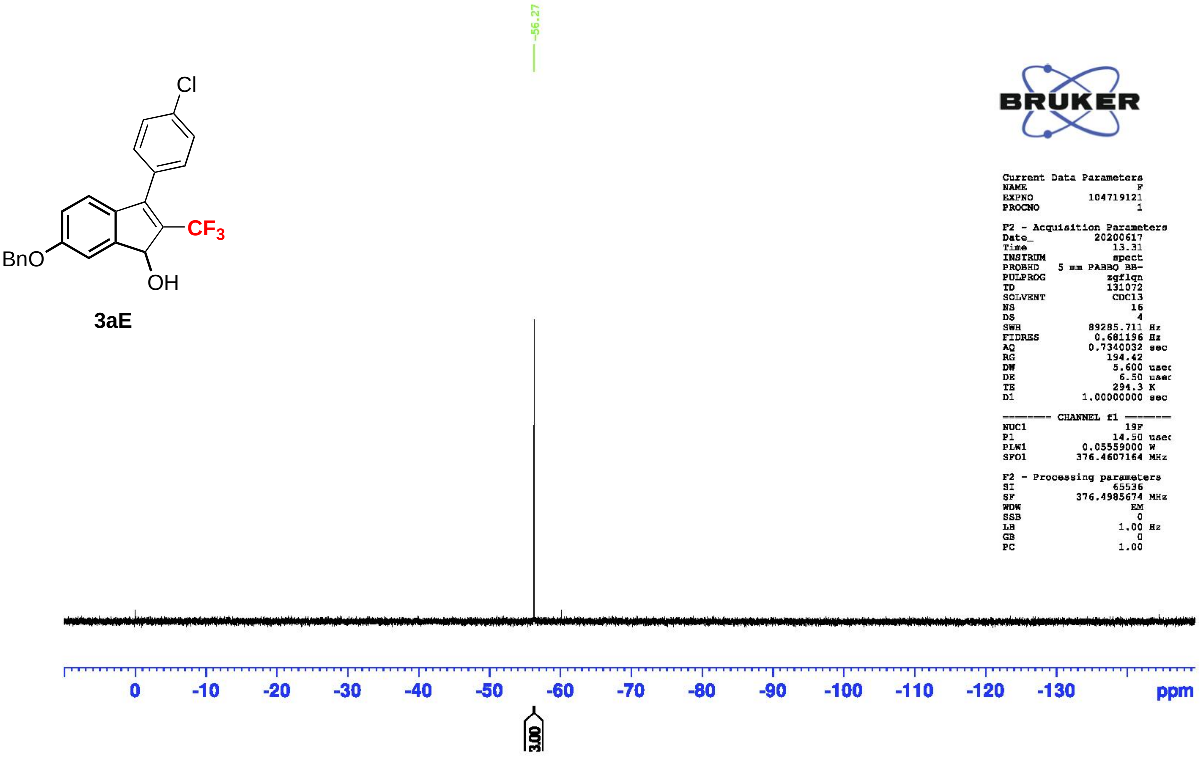

^{1}H NMR spectrum of **3-(4-Chlorophenyl)-5-fluoro-2-trifluoromethyl-1*H*-inden-1-ol (3aF)**

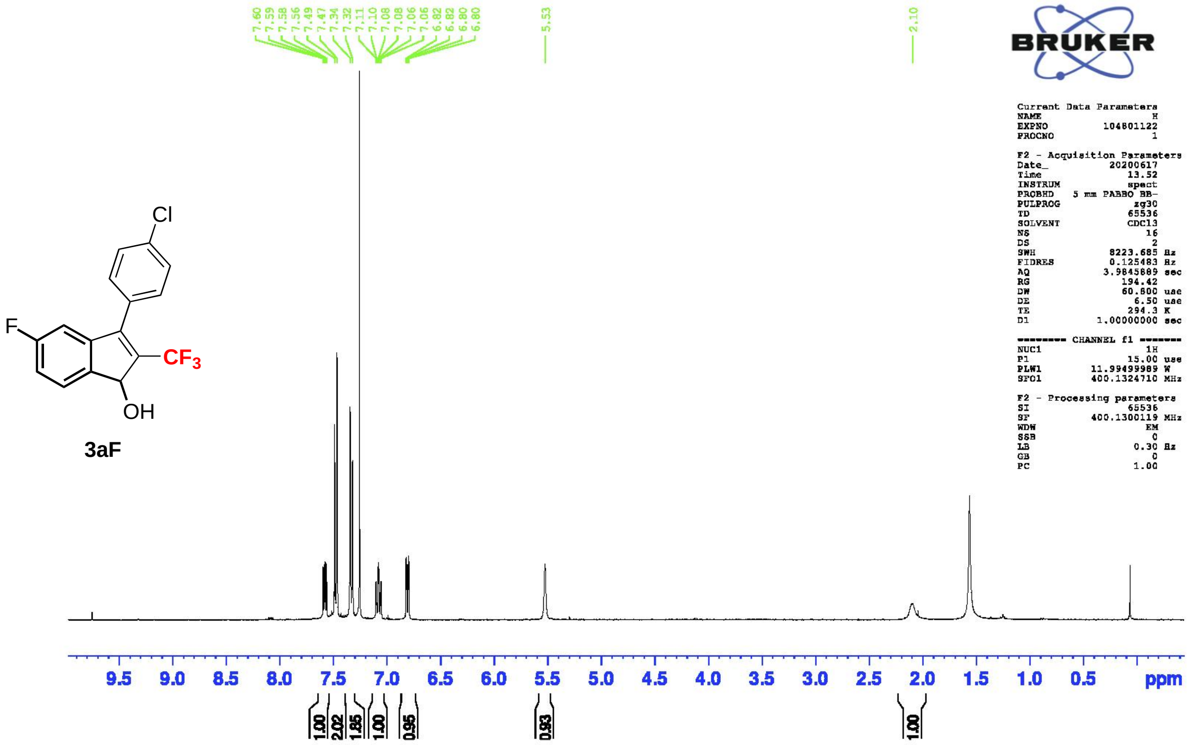

^{13}C NMR spectrum of **3-(4-Chlorophenyl)-5-fluoro-2-trifluoromethyl-1*H*-inden-1-ol (3aF)**

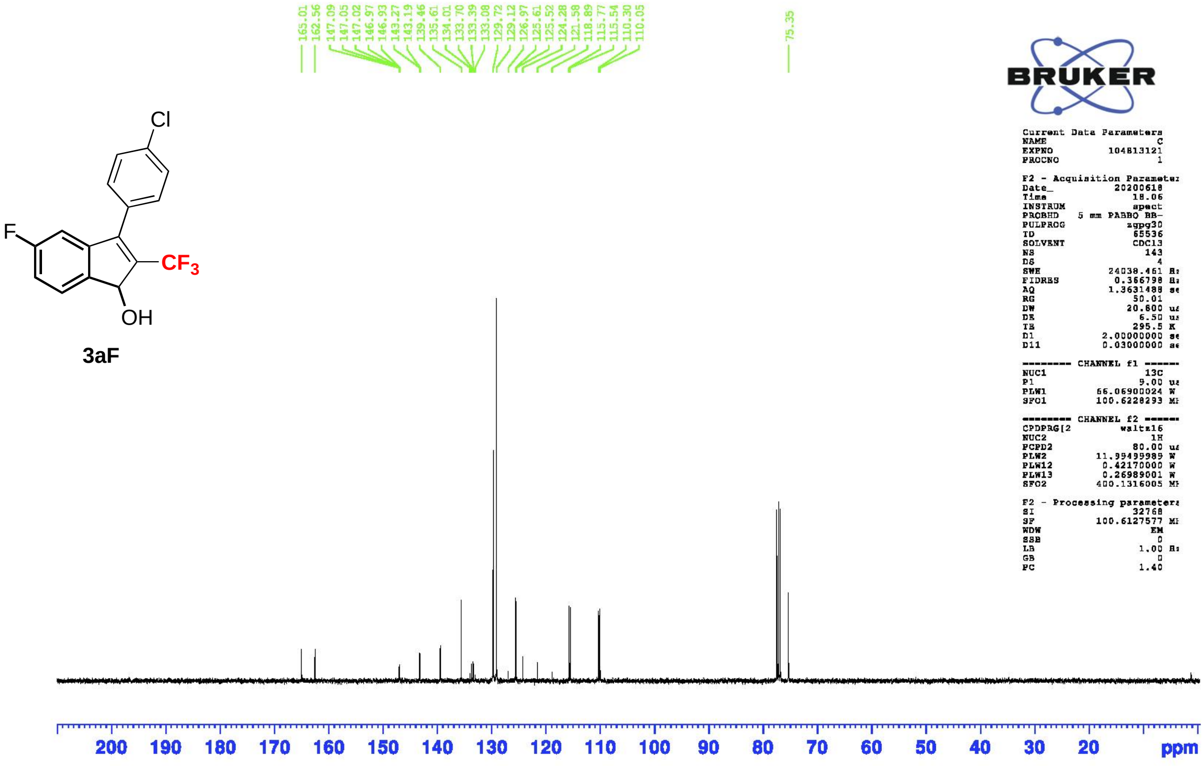

^{19}F NMR spectrum of **3-(4-Chlorophenyl)-5-fluoro-2-trifluoromethyl-1*H*-inden-1-ol (3aF)**

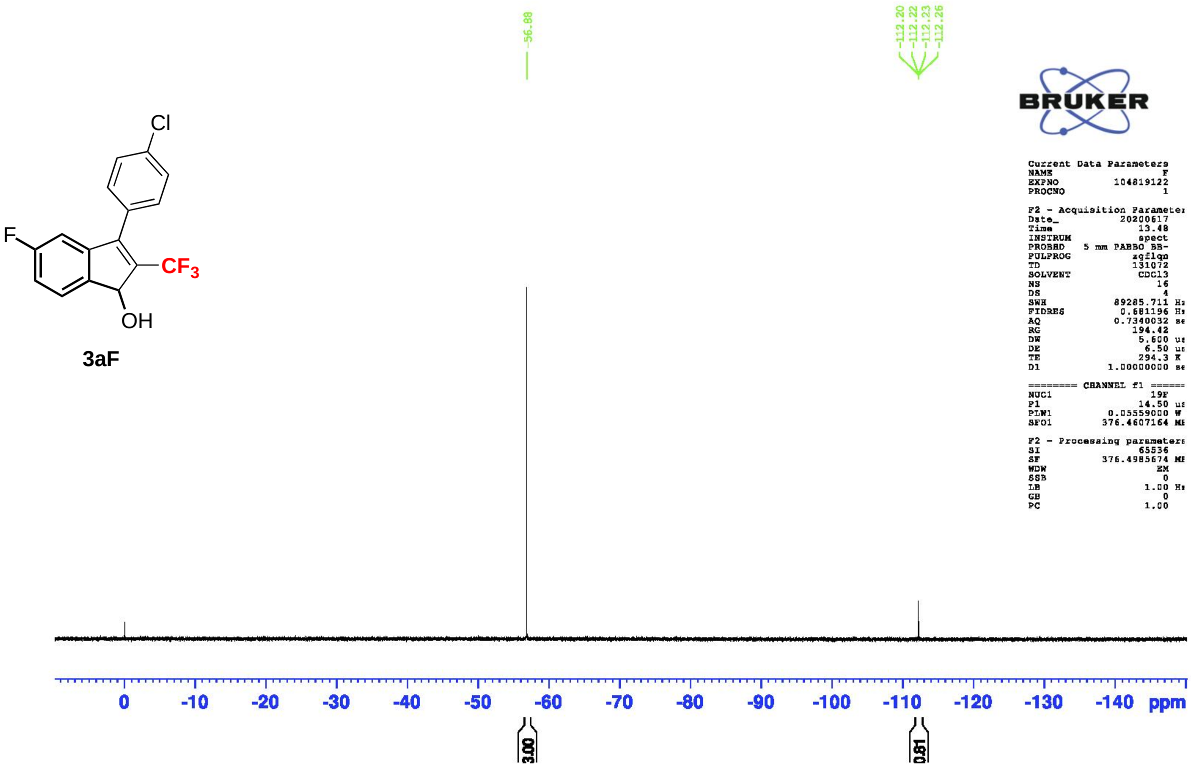

^{1}H NMR spectrum of **3-(4-Chlorophenyl)-7-fluoro-2-trifluoromethyl-1*H*-inden-1-ol (3aG)**

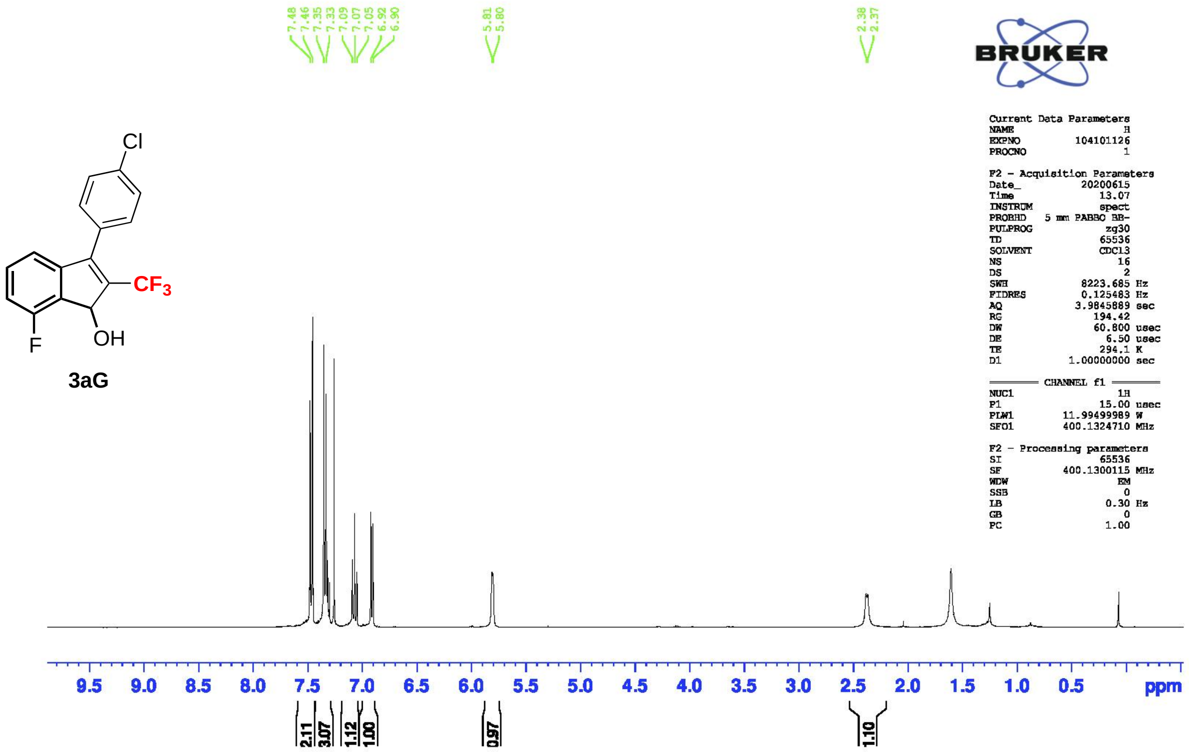

^{13}C NMR spectrum of **3-(4-Chlorophenyl)-7-fluoro-2-trifluoromethyl-1*H*-inden-1-ol (3aG)**

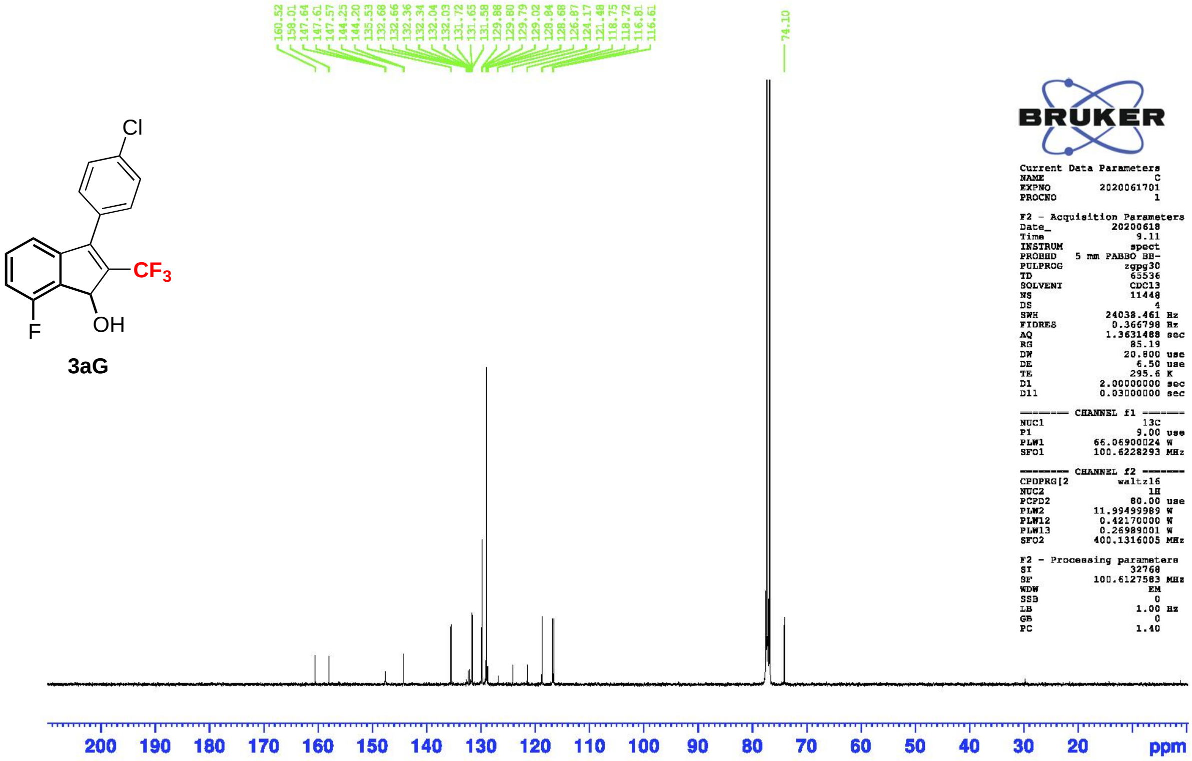

^{19}F NMR spectrum of **3-(4-Chlorophenyl)-7-fluoro-2-trifluoromethyl-1*H*-inden-1-ol (3aG)**

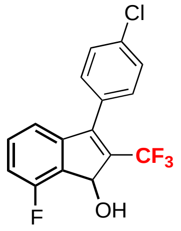

3aG

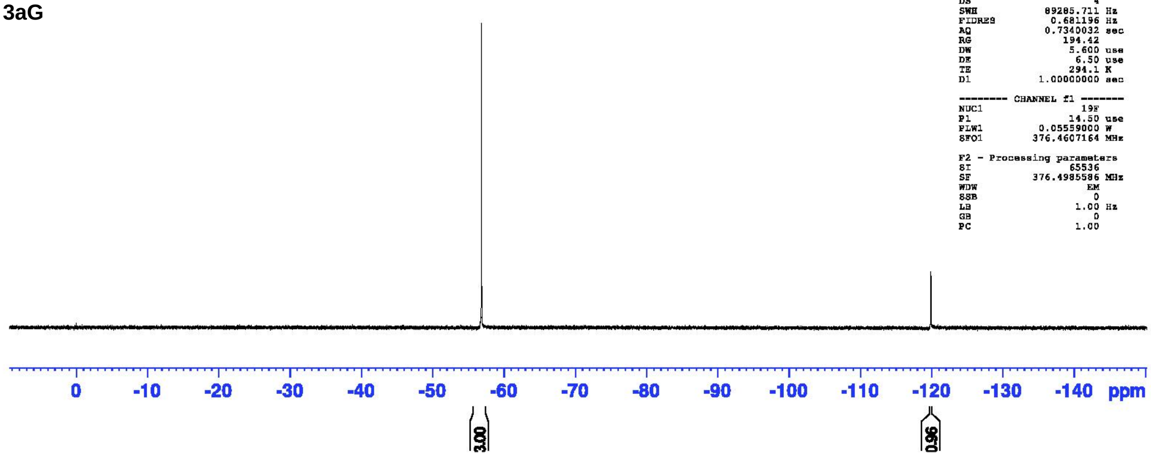

^{1}H NMR spectrum of **2-(4-Chlorophenyl)-3-trifluoromethyl-2,3-dihydro-1*H*-indan-1-one (5aA)**

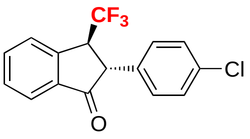

5aA

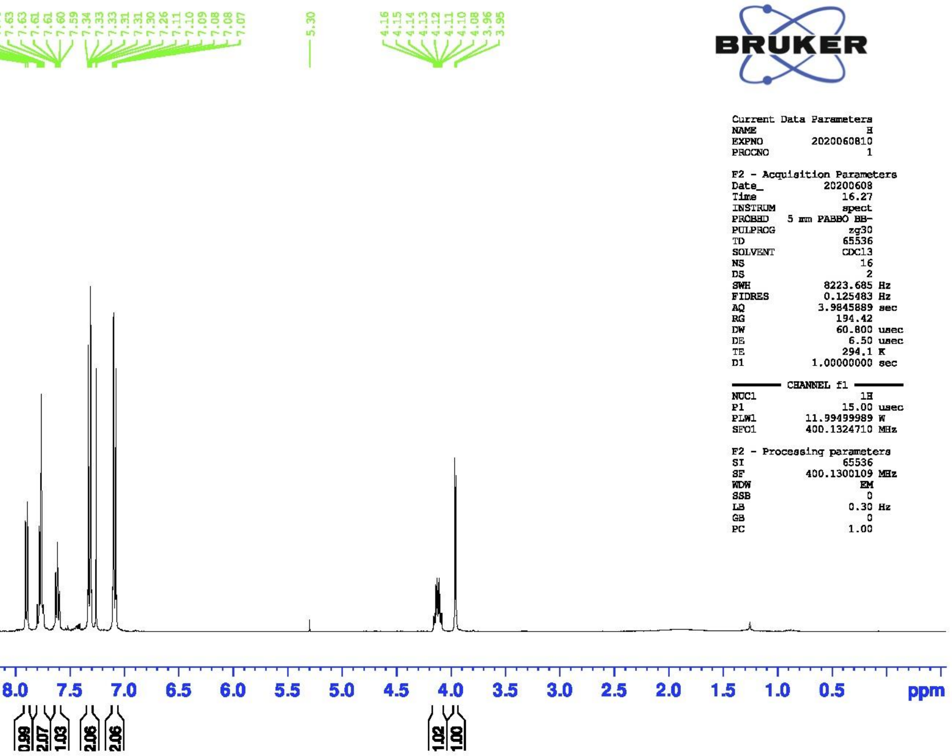

^{13}C NMR spectrum of **2-(4-Chlorophenyl)-3-trifluoromethyl-2,3-dihydro-1*H*-indan-1-one (5aA)**

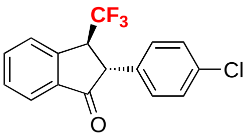

5aA

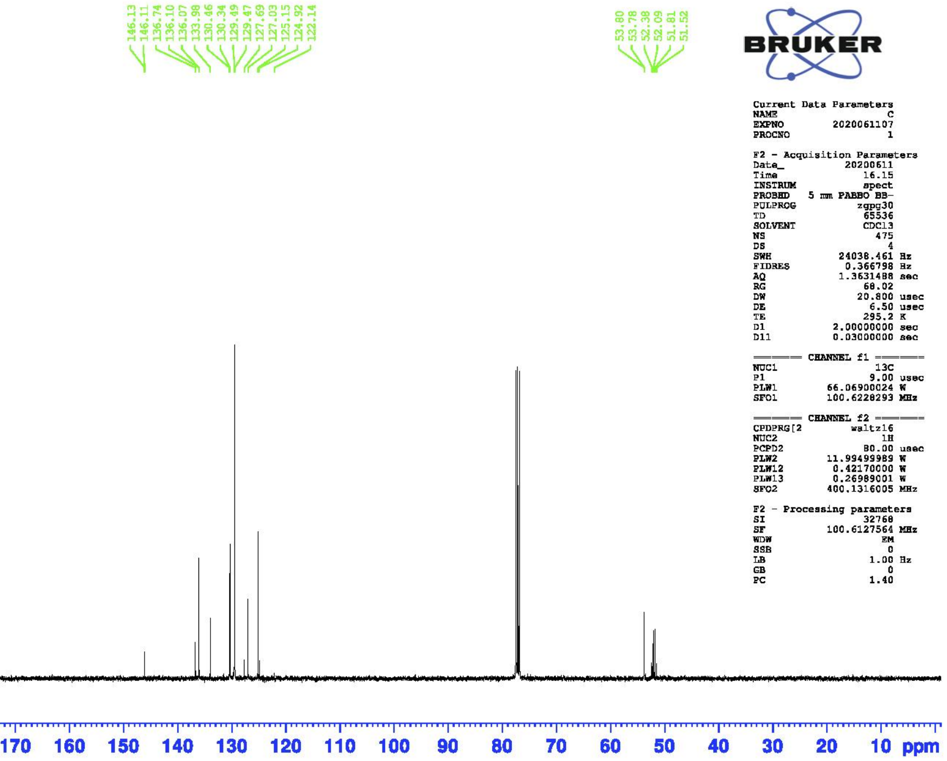

^{19}F NMR spectrum of **2-(4-Chlorophenyl)-3-trifluoromethyl-2,3-dihydro-1*H*-indan-1-one (5aA)**

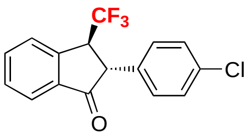

5aA

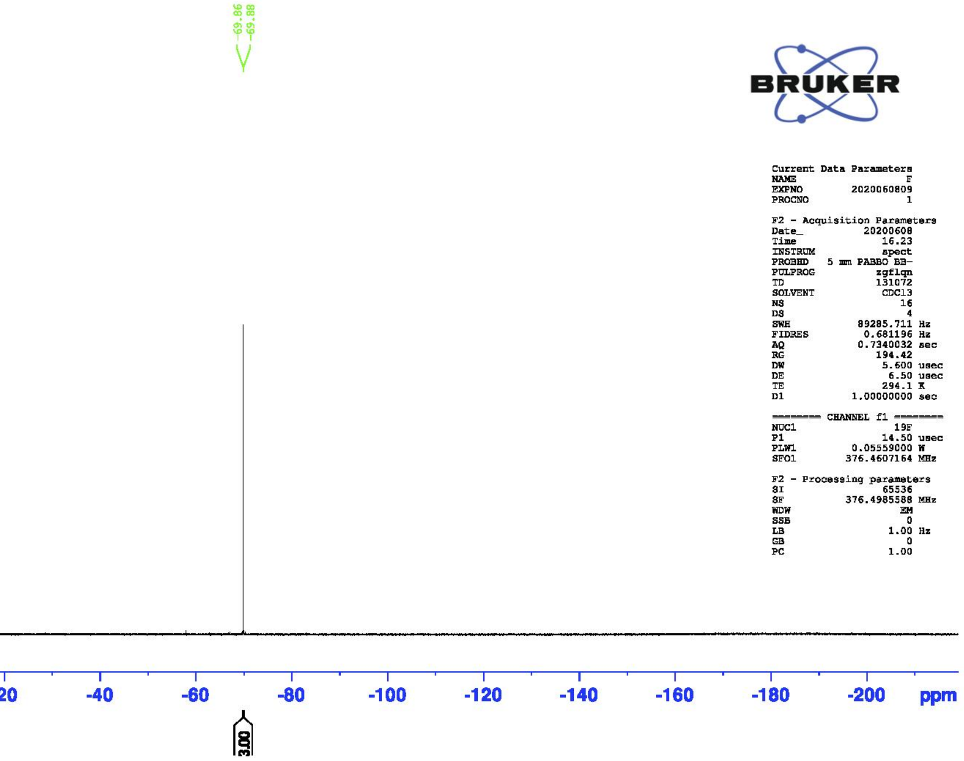

^{1}H NMR spectrum of **3-(4-Chlorophenyl)-2-trifluoromethyl-inden-1-one (6)**

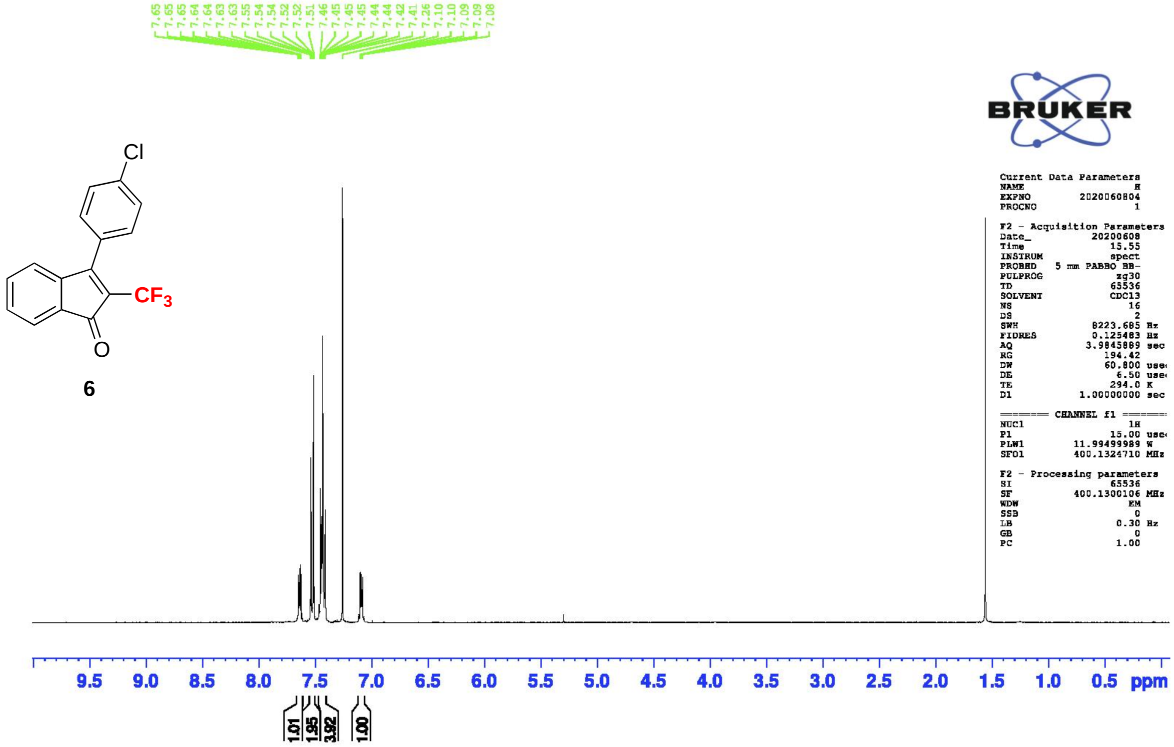

^{13}C NMR spectrum of **3-(4-Chlorophenyl)-2-trifluoromethyl-inden-1-one (6)**

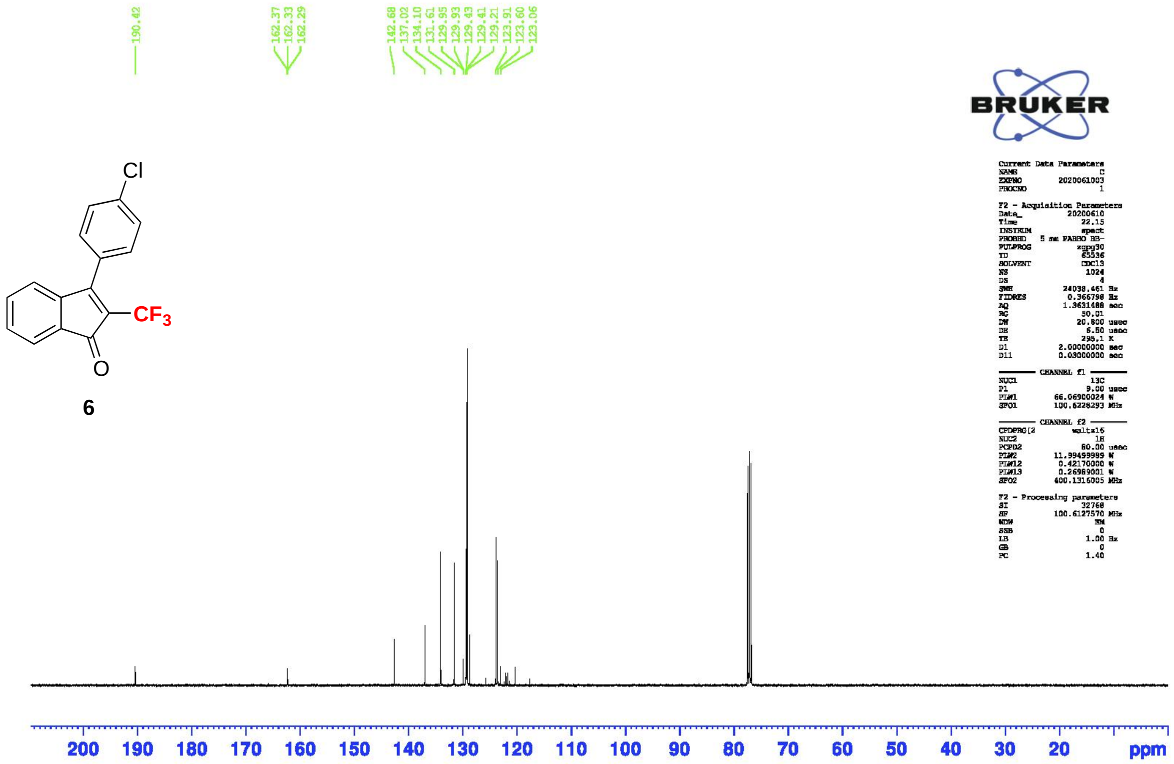

^{19}F NMR spectrum of **3-(4-Chlorophenyl)-2-trifluoromethyl-inden-1-one (6)**

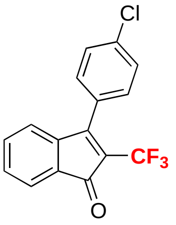

6

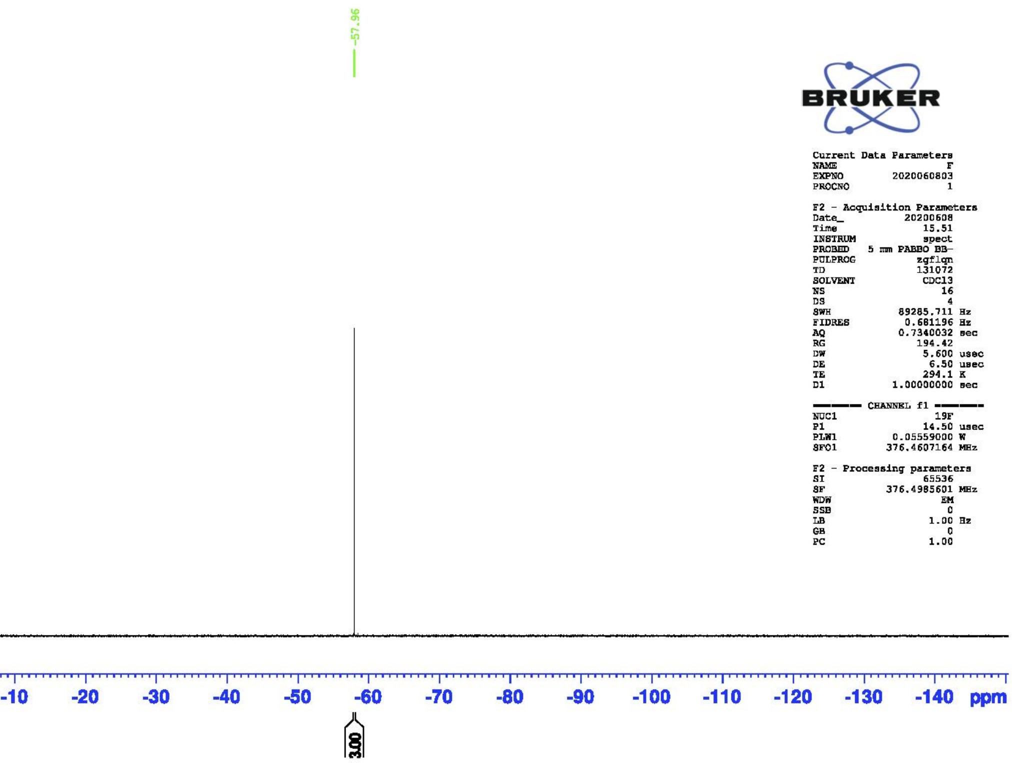

^{1}H NMR spectrum of **3-(4-Chlorophenyl)-2-trifluoromethyl-2,3-dihydro-1*H*-indan-1-one (7)**

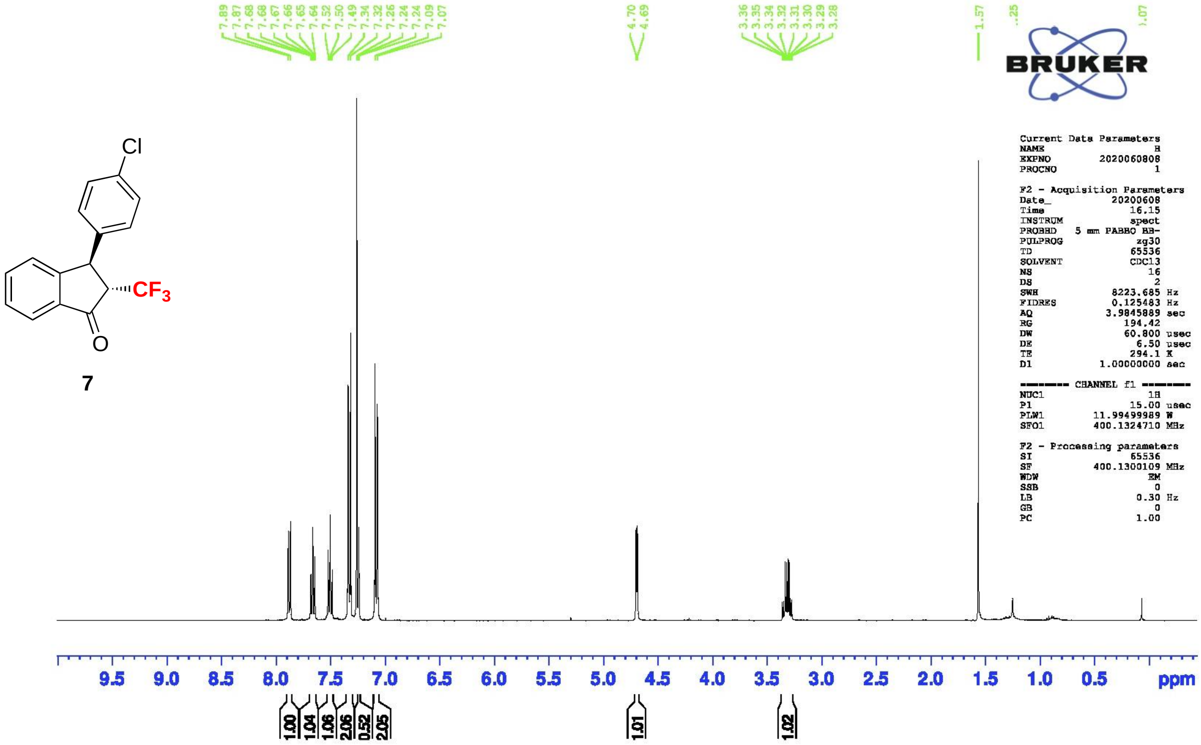

^{13}C NMR spectrum of **3-(4-Chlorophenyl)-2-trifluoromethyl-2,3-dihydro-1*H*-indan-1-one (7)**

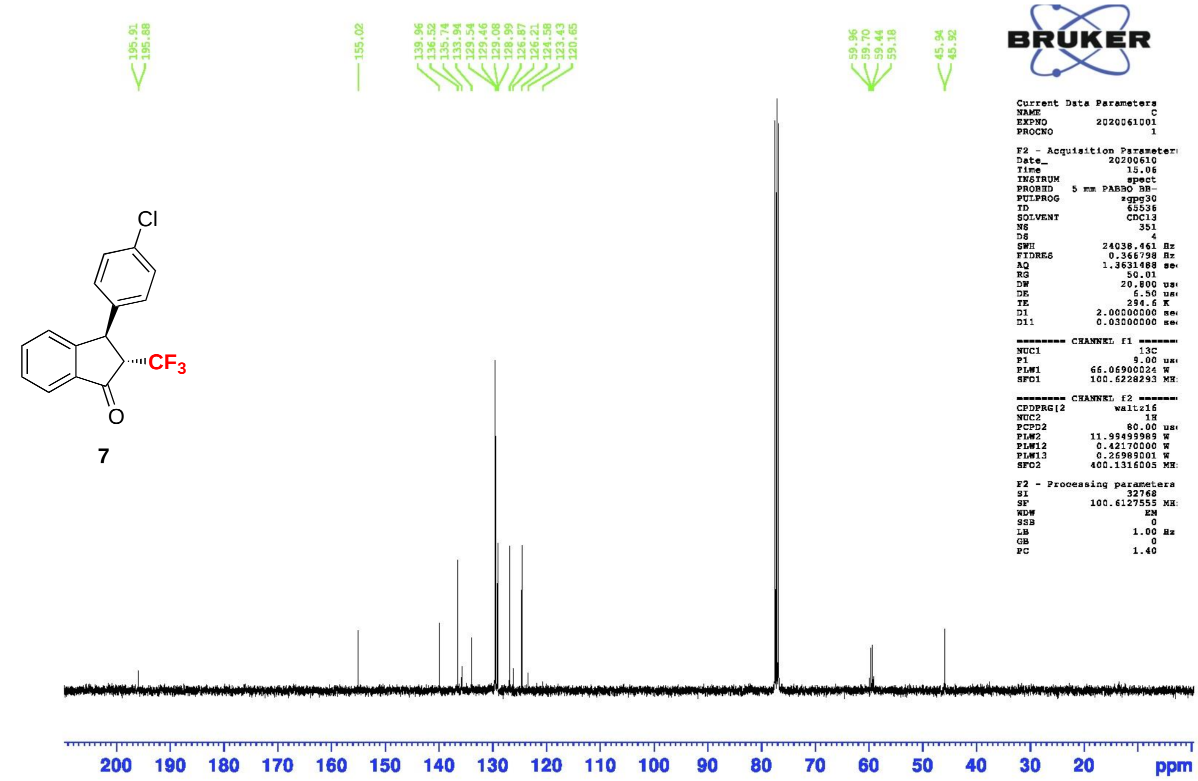

^{19}F NMR spectrum of **3-(4-Chlorophenyl)-2-trifluoromethyl-2,3-dihydro-1*H*-indan-1-one (7)**

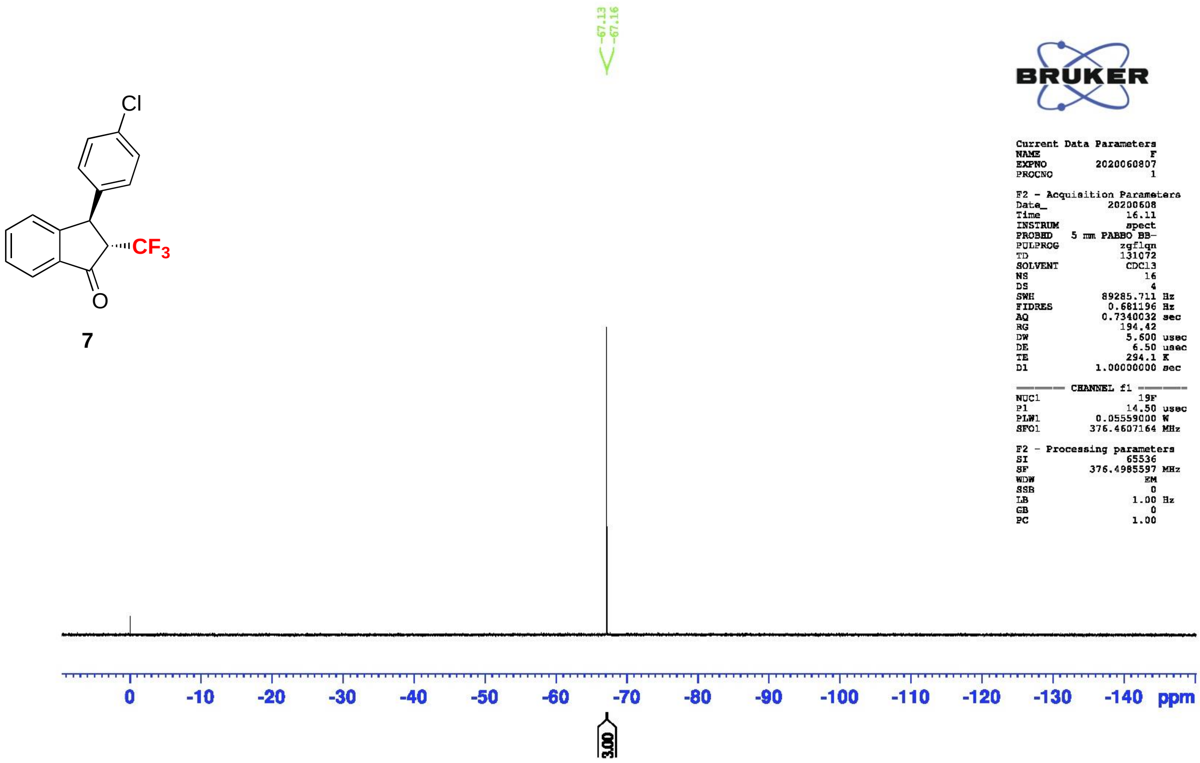

7

HMBC spectrum of **2-(4-Chlorophenyl)-3-trifluoromethyl-2,3-dihydro-1*H*-indan-1-one (5aA)**

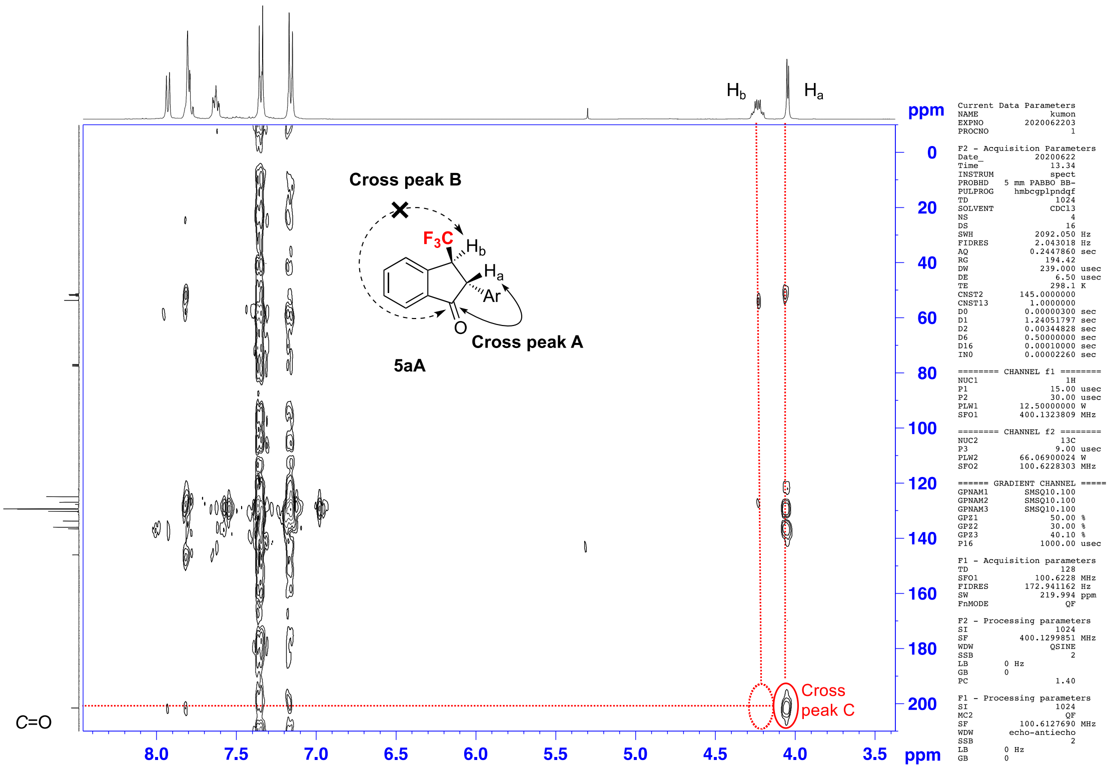

HMBC spectrum of **3-(4-Chlorophenyl)-2-trifluoromethyl-2,3-dihydro-1*H*-indan-1-one (7)**

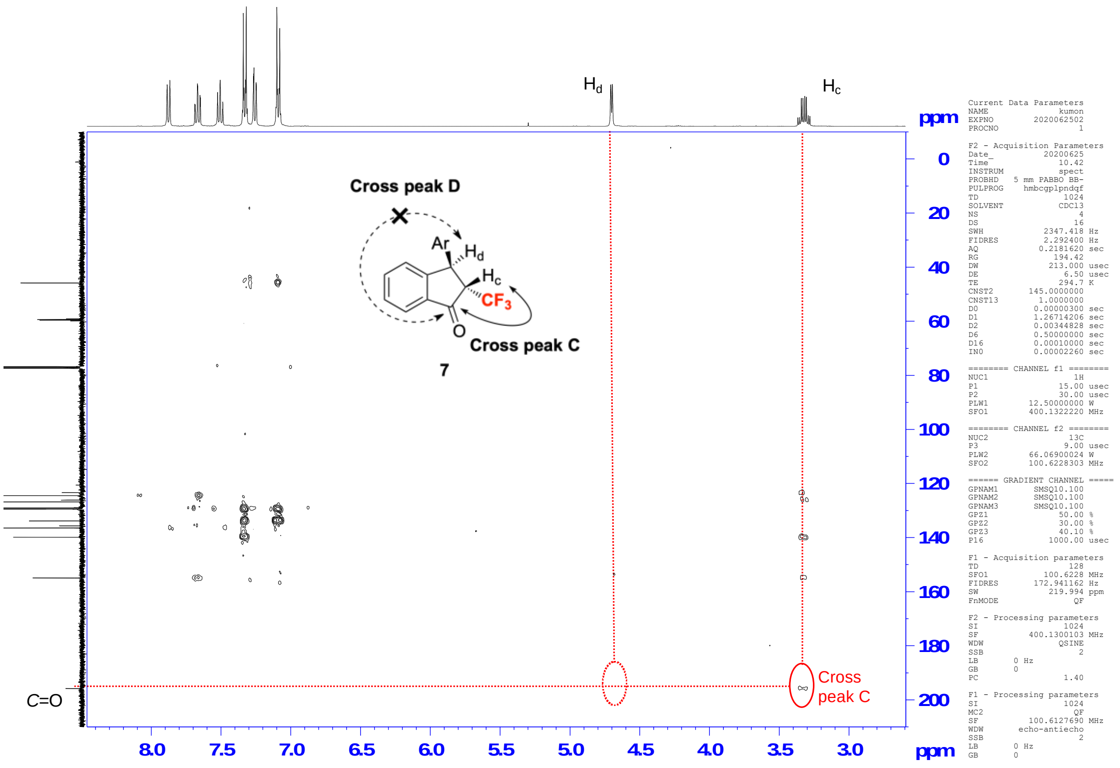

5. References

1. Hiyama, T.; Sato, K.; Fujita, M. *Bull. Chem. Soc. Jpn.* **1989**, *62*, 1352–1354.

2. Konno, T.; Chae, J.; Kanda, M.; Nagai, G.; Tamura, K.; Ishihara, T.; Yamanaka, H. *Tetrahedron* **2003**, *59*, 7571–7580.
